# Supplementary material for: The economic value of Canada’s National Capital Green Network
Source: PLoS One. 2021 Jan 19;16(1):e0245045. doi: 10.1371/journal.pone.0245045 (PMC7815161; doi:10.1371/journal.pone.0245045)
Supplement: S1 Table — (DOCX) [file pone.0245045.s001.docx]

**S1 Table. List of studies used as part of the ES valuation with benefit transfer (Values in $2015 CAD)**

| **ES** | **Source** | **Country** | **Value ($/ha/yr)** | **Method** |
| --- | --- | --- | --- | --- |
| **Urban Forests and woodlands** | | | | |
| Habitat for biodiversity | [65] Kniivila et al. (2002) | Finland | 449.50 | CV |
|  | [66] Loomis et Ekstrand (1998) | United States | 7159.70 | CV |
|  | [67] Siiakmäki et Layton (2007) | Finland | 444.10 | CV |
| Disturbance prevention | [62] Morri et al. (2014) | Italy | 4974.60 | AC |
|  | [62] Morri et al. (2014) | Italy | 5085.00 | AC |
| Water provisioning | [61] Hein (2011) | Netherlands | 609.08 | RC |
|  | [62] Morri et al. (2014) | Italy | 206.51 | MP |
|  | [62] Morri et al. (2014) | Italy | 202.56 | MP |
| Waste treatment | [62] Morri et al. (2014) | Italy | 110.49 | RC |
| Erosion control | [62] Morri et al. (2014) | Europe | 139.87 | RC |
|  | [63] Croitoru (2007) | Italy | 126.27 | RC |
|  | [64] Yoo et al. (2014) | United States | 396.14 | AC, HP |
| Pest management | [9] Dupras et Alam 2014 | Canada | 45.00 | BT |
| Air quality | [60] Hirabayashi 2010 | United States | 554.00 | BT |
| **Rural forest** | | | | |
| Habitat for biodiversity | [65] Kniivila et al. (2002) | Finland | 460.60 | CV |
|  | [66] Loomis et Ekstrand (1998) | United States | 7159.70 | CV |
|  | [67] Siiakmäki et Layton (2007) | Finland | 444.10 | CV |
|  | [72] Garrod et Willis (1997) | United Kingdom | 11349.00 | CE |
|  | [72] Garrod et Willis (1997) | United Kingdom | 4115.60 | CE |
|  | [72] Garrod et Willis (1997) | United Kingdom | 6687.90 | CE |
|  | [73] Knowler et al. (2003) | Canada | 2.25 | PF |
|  | [74] Ovando et al. (2016) | Spain | 23.29 | DC |
|  | [75] Roesch-McNally et al. (2016) | United States | 0.05 | CV |
|  | [75] Roesch-McNally et al. (2016) | United States | 2.54 | CV |
|  | [75] Roesch-McNally et al. (2016) | United States | 0.07 | CV |
|  | [75] Roesch-McNally et al. (2016) | United States | 6.81 | CV |
|  | [76] Scarpa et al. (2000) | Ireland | 218.26 | CV |
|  | [77] Walsh et al. (1984) | United States | 125.61 | CV |
| Air quality | [68] Remme et al. (2015) | Netherlands | 9.70 | AC |
| Water provisioning | [61] Hein (2011) | Netherlands | 609.08 | RC |
|  | [62] Morri et al. (2014) | Italy | 206.51 | MP |
|  | [62] Morri et al. (2014) | Italy | 202.56 | MP |
|  | [69] Häyhä et al. (2015) | Italy | 122.52 | MP |
|  | [71] Xue and Tisdell (2001) | China | 3053.00 | RC |
| Nutrient cycling | [70] Ninan et Inoue (2013) | Japan | 0.13 | MP |
|  | [70] Ninan et Inoue (2013) | Japan | 107.29 | MP |
|  | [71] Xue et Tisdell (2001) | China | 848.39 | RC |
| Waste treatment | [63] Croitoru (2007) | Europe | 139.87 | RC |
|  | [70] Ninan et Inoue (2013) | Japan | 26.03 | MP |
|  | [70] Ninan et Inoue (2013) | Japan | 805.98 | MP |
|  | [71] Xue and Tisdell (2001) | China | 298.18 | RC |
| Erosion control | [62] Morri et al. (2014) | Italy | 126.27 | RC |
|  | [62] Morri et al. (2014) | Italy | 110.49 | RC |
|  | [69] Häyhä et al. (2015) | Italy | 535.90 | RC |
|  | [70] Ninan et Inoue (2013) | Japan | 5.35 | HP |
|  | [70] Ninan et Inoue (2013) | Japan | 0.54 | OC |
|  | [71] Xue and Tisdell (2001) | China | 43.02 | OC |
| Pest management | [9] Dupras et Alam 2014 | Canada | 45.00 | BT |
|  | [71] Xue and Tisdell (2001) | China | 13.97 | RC |
| **Wetlands** | | | | |
| Water provisioning | [78] Folke (1991) | Sweden | 53.76 | RC |
|  | [79] Farber (1996) | United States | 7.99 | RC |
| **Croplands** | | | | |
| Erosion control | [80] Fox et Dickson (1990) | United States | 60.87 | AC |
|  | [81] Pimentel et al. (1995) | United States | 72.65 | RC |
|  | [81] Pimentel et al. (1995) | United States | 193.16 | RC |
| Nutrient cycling | [9] Dupras et Alam (2014) | Canada | 184.00 | BT |
| Aesthetics | [82] Dupras et Revéret (2013) | Canada | 136.49 | CE |
|  | [83] Alvarez-Farizo et al. (1999) | United Kingdom | 36.88 | CV |
|  | [84] Bastian et al. (2002) | United States | 21.10 | HP |
|  | [85] Bowker et Diychuck (1994) | Canada | 191.20 | CV |
|  | [86] Sandhu et al. (2008) | New Zealand | 30.02 | CE |
| Recreation | [9] Dupras et Alam (2014) | Canada | 94.00 | MP |
| **Prairie and grasslands** | | | | |
| Erosion control | [80] Fox et Dickson (1990) | United States | 60.87 | AC |
|  | [81] Pimentel et al. (1995) | United States | 72.65 | RC |
|  | [81] Pimentel et al. (1995) | United States | 193.16 | RC |
| Habitat for biodiversity | [9] Dupras et Alam (2014) | Canada | 2467.00 | BT |
| Pest management | [9] Dupras et Alam (2014) | Canada | 45.00 | BT |
| Nutrient cycling | [81] Pimentel et al. (1995) | United States | 146.74 | RC |
| Aesthetics | [82] Dupras et Revéret (2013) | Canada | 136.49 | CE |
|  | [83] Alvarez-Farizo et al. (1999) | United Kingdom | 36.88 | CV |
|  | [84] Bastian et al. (2002) | United States | 21.10 | HP |
|  | [85] Bowker et Diychuck (1994) | Canada | 191.20 | CV |
|  | [86] Sandhu et al. (2008) | New Zealand | 30.02 | CE |
| **Freshwater** | | | | |
| Habitat for biodiversity | [87] Poder et al. (2015) | Canada | 10.00 | CR |
| Waste treatment | [87] Poder et al. (2015) | Canada | 48.00 | CR |
| Aesthetics | [87] Poder et al. (2015) | Canada | 4.00 | CR |
| AC (Avoided cost), BT (Benefit transfer), CE (Choice experiment), CV (Contingent valuation), CR: Contingent ranking, DC (Damage cost), HP (Hedonic pricing), MP (Market price), OC (Opportunity cost); RC (Replacement cost). | | | | |
