# Supplementary material for: The economic value of Canada’s National Capital Green Network
Source: PLoS One. 2021 Jan 19;16(1):e0245045. doi: 10.1371/journal.pone.0245045 (PMC7815161; doi:10.1371/journal.pone.0245045)
Supplement: S2 Table — (DOCX) [file pone.0245045.s002.docx]

**S2 Table. Conversion of the WTP values from Poder et al. [87] to values for the NCC Green Network.**

| **Ecosystem Services** | **WTP for the Montreal Blue Network ($/hh)** | **Nb of Households in the Ottawa/Gatineau Region** | **Freshwater Systems area in the Ottawa/Gatineau Region (ha)** | **Value for the NCCGN services ($/ha/y)** |
| --- | --- | --- | --- | --- |
| Biodiversity Habitat | 25 | 340,515 | 24,897 | 10 |
| Waste Treatment | 218 | 340,515 | 24,897 | 48 |
| Aesthetics | 10 | 340,515 | 24,897 | 4 |

*Adapted from Dupras et al. [S1 File]*
