# Supplementary material for: The economic value of Canada’s National Capital Green Network
Source: PLoS One. 2021 Jan 19;16(1):e0245045. doi: 10.1371/journal.pone.0245045 (PMC7815161; doi:10.1371/journal.pone.0245045)
Supplement: S1 File — The Economic Value of the National Capital Commission’s Green Network. Dupras J, L’Ecuyer-Sauvageau C, Auclair J, He J, Poder T. Natural Capital. The Economic Value of the National Capital Commission’s Green Network. David Suzuki Foundation & National Capital Commission. 2016. 51 pages. (PDF) [file pone.0245045.s003.pdf]

NATURAL CAPITAL

THE ECONOMIC VALUE OF THE NATIONAL CAPITAL COMMISSION'S GREEN NETWORK

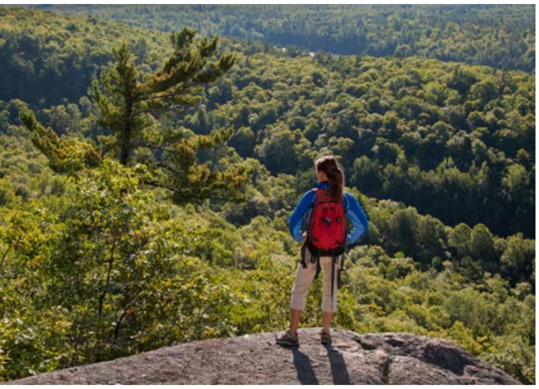

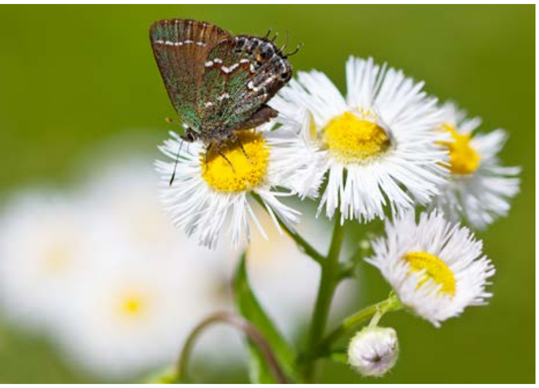

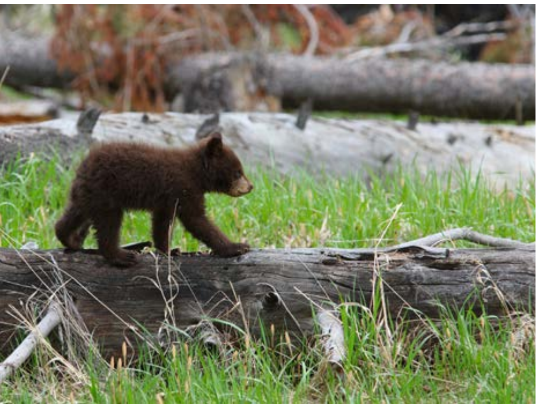

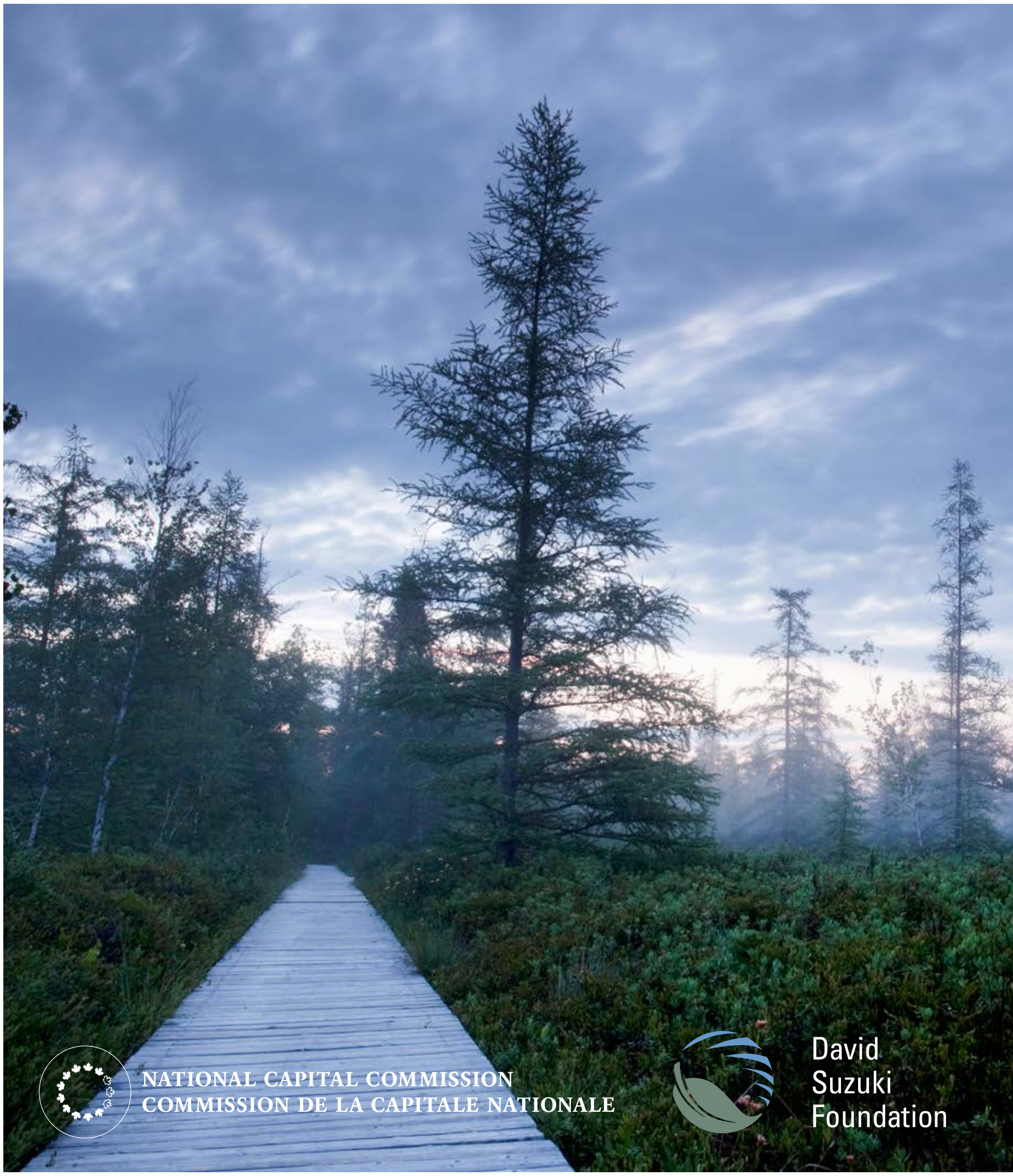

NATIONAL CAPITAL COMMISSION
COMMISSION DE LA CAPITALE NATIONALE

David
Suzuki
Foundation

NATURAL CAPITAL

THE ECONOMIC VALUE OF THE NATIONAL CAPITAL COMMISSION'S GREEN NETWORK

December 2016

Jérôme Dupras*, Chloé L'Ecuyer-Sauvageau*, Jeoffrey Auclair*, Jie He**, Thomas Poder**

* Ecological Economics Laboratory, Université du Québec en Outaouais (dupraslab.weebly.com)
** Département d'économique, Université de Sherbrooke

The content of this study is the responsibility of the authors.

Photos: National Capital Commission (except where noted)

Cover photos: NCC; Epantha/iStock (butterfly); and Nathan Hobbs/iStock (bear cub)

Graphic design: Nadene Rehnby, Hands On Publications

ISBN: 978-0-9949543-5-0

Disponible en français à : davidsuzuki.org/fr/publications
and ccn-ncc.gc.ca/a-propos-de-la-ccn/rapports-publications

Contact: Catherine Verreault, Senior Manager,
Natural Resources and Land Management, Gatineau Park, National Capital Commission

NATIONAL CAPITAL COMMISSION
COMMISSION DE LA CAPITALE NATIONALE

202 – 40 Elgin Street, Ottawa, Ontario, K1P 1C7
Telephone: 613-239-5000 or 1-800-465-1867
Email: info@ncc-ccn.ca
ncc-ccn.gc.ca

David Suzuki Foundation

Montreal Office
540 – 50, rue Sainte-Catherine Ouest, Montreal QC, H2X 3V4
Telephone: 514-871-4932 Email: contact@davidsuzuki.org
davidsuzuki.org

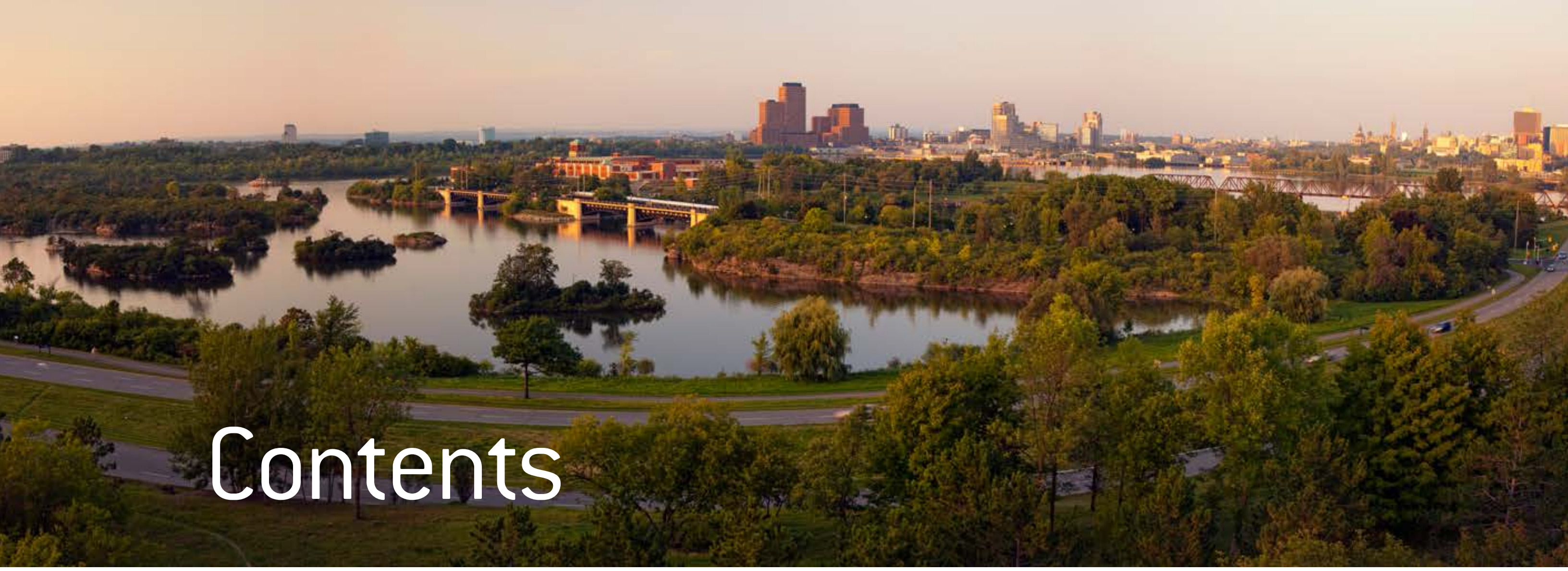

Contents

EXECUTIVE SUMMARY 5

SECTION 1: INTRODUCTION 7

SECTION 2: NATIONAL CAPITAL COMMISSION'S GREEN NETWORK IN CANADA'S CAPITAL REGION 10

2.1 The National Capital Region Portrait 10
2.2 Gatineau Park 11
2.3 Greenbelt 12
2.4 Urban Lands 13

SECTION 3: ECOSYSTEM SERVICES VALUATION 14

3.1 Historical and Conceptual Perspectives 14
3.2 Tools and Methods of Economic Valuation 16
3.3 Examples of Ecosystem Services Valuation of Urban Natural Capital in Canada .. 17
3.4 Ecosystem Services Valuation in Decision-Making 18

SECTION 4: METHODOLOGY 19

4.1 Spatial Designation of the Study Area 19
4.2 Classification and Mapping of Land Use Cover 20
4.3 Ecosystem Services Valuation 22

SECTION 5: RESULTS 25

5.1 Spatial Analysis 25
5.2 Ecosystem Services Valuation 27
5.3 Forests and Woodlands 28
5.4 Wetlands 33
5.5 Croplands 37
5.6 Prairies, Pastures and Grasslands 40
5.7 Freshwater Systems 42
5.8 Total Economic Value 44

SECTION 6: CONCLUSION 47

REFERENCES 48

Download other publications in this series at www.davidsuzuki.org/publications/

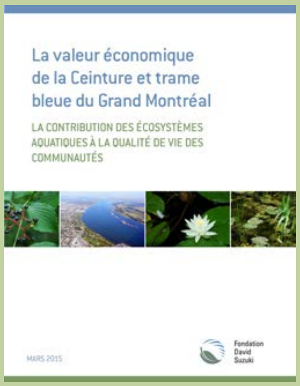

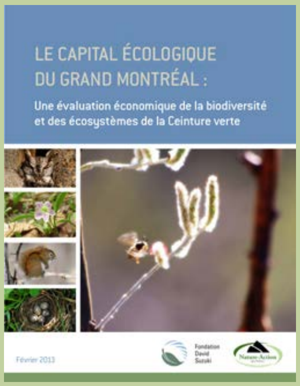

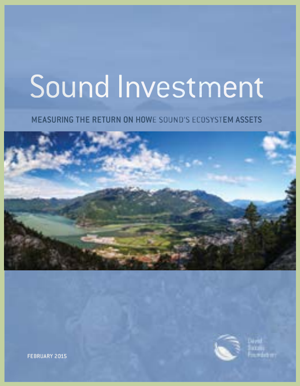

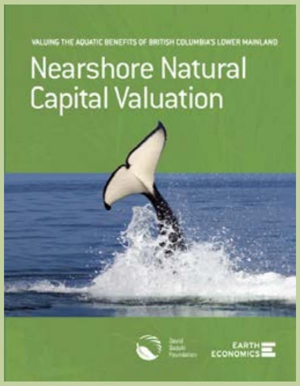

List of acronyms

AAFC	Agriculture and Agri-Food Canada
CICES	Common International Classification of Ecosystem Goods and Services
CO_2	Carbon Dioxide
CRAAQ	*Centre de référence en agriculture et agroalimentaire du Québec*
ECCC	Environment and Climate Change Canada
ES	Ecosystem service
EVRI	Environmental Valuation Reference Inventory
FADQ	*Financière agricole du Québec*
GDP	Gross Domestic Product
GIS	Geographic Information System
MDDELCC	*Ministère du Développement durable, de l'Environnement et de la Lutte contre les changements climatiques*
MEA	Millennium Ecosystem Assessment
NCC	National Capital Commission
NCCGN	National Capital Commission's Green Network
NCR	National Capital Region
OMAFRA	Ontario Ministry of Agriculture, Food and Rural Affairs
PPP	Purchasing Power Parity
SCC	Social Cost of Carbon
TEEB	The Economics of Ecosystems and Biodiversity

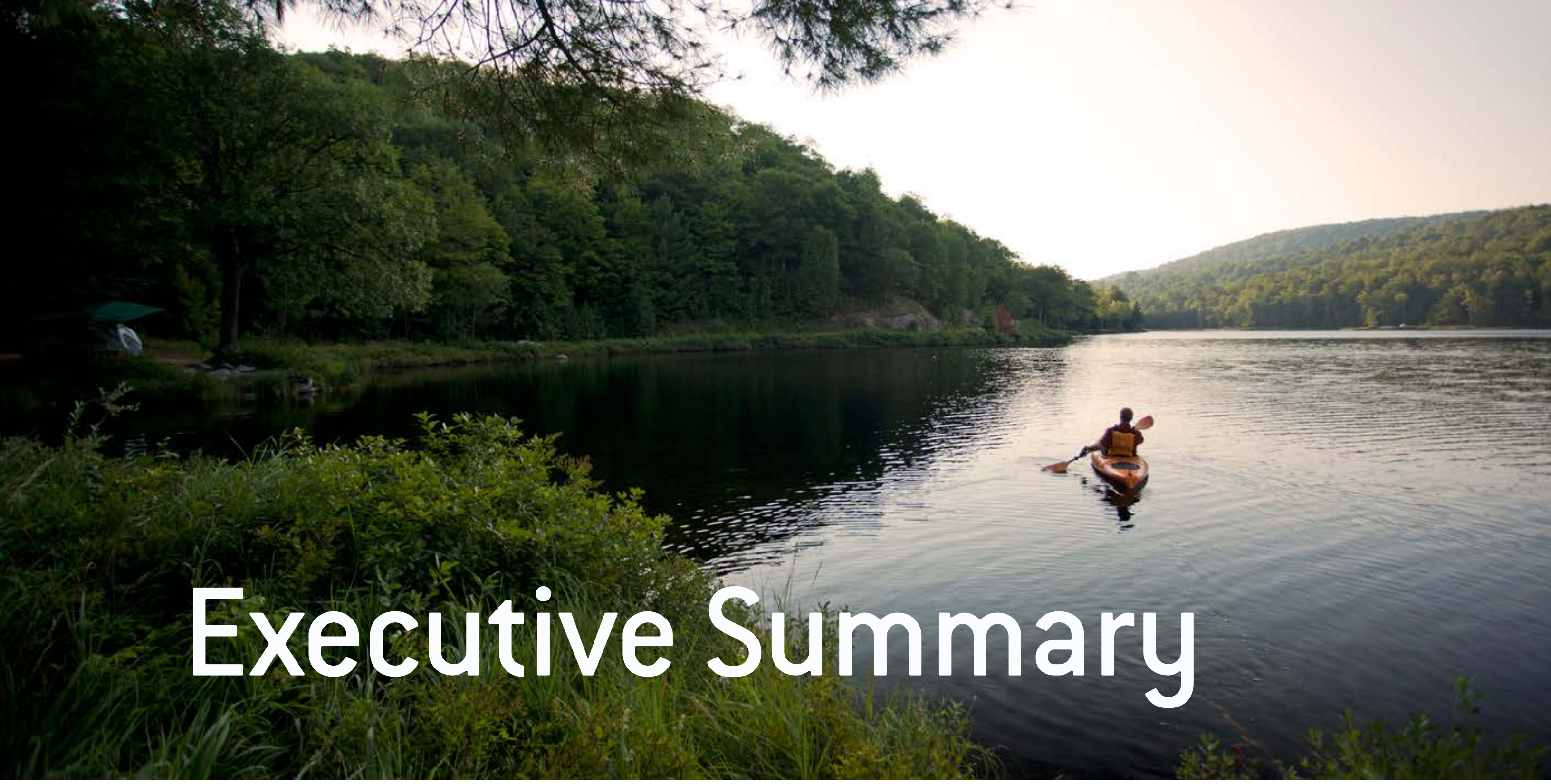

Executive Summary

THE CAPITAL'S URBAN CORE is dappled with parks, and encircled on three sides by the Greenbelt. To the north of the core, only minutes away, lies the National Capital Region's gateway to Canada's storied north: Gatineau Park. Tasked with the stewardship of this remarkable natural heritage is the National Capital Commission, which protects and conserves more than 55,000 hectares of federal lands within the NCR.

Much of the land in the NCC's care is green space, much of it in its natural state, consisting of forest, wetlands, and freshwater, as well as agricultural lands and much of the urban parkland.

For thousands of years this region has been defined by its rivers, and its modern history was determined by the unshakeable resolve of the builders of its iconic canal. Today, the NCC owns, maintains, and develops as appropriate the Capital's historic waterfronts: 35 kilometres along the Ottawa River, 16 kilometres along the Rideau River, and 4 kilometres along the Gatineau River, as well as 20 kilometres along the Rideau Canal.

Everyone recognizes the importance of these green spaces to the enviable quality of life enjoyed by the Capital's residents, but that is only part of their value. This study captures the total economic vitality of the NCC's green spaces natural capital.

The NCC's green spaces provide direct monetary benefits, such as from wood and agricultural products. But they also provide great value in benefits not measured according to traditional market metrics, such as:

- Air quality control;
- Water filtration;
- Climate regulation;
- Carbon storage;
- Wildlife habitat; and
- And erosion control.

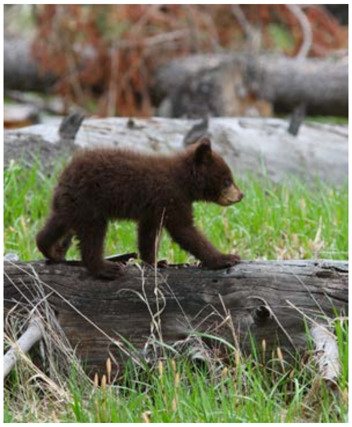

Everyone recognizes the importance of these green spaces to the enviable quality of life enjoyed by the Capital's residents, but that is only part of their value. This study captures the total economic vitality of the NCC's green spaces natural capital.

TOP PHOTO: NATIONAL CAPITAL COMMISSION

BOTTOM PHOTO: NATHAN HOBBS/ISTOCK

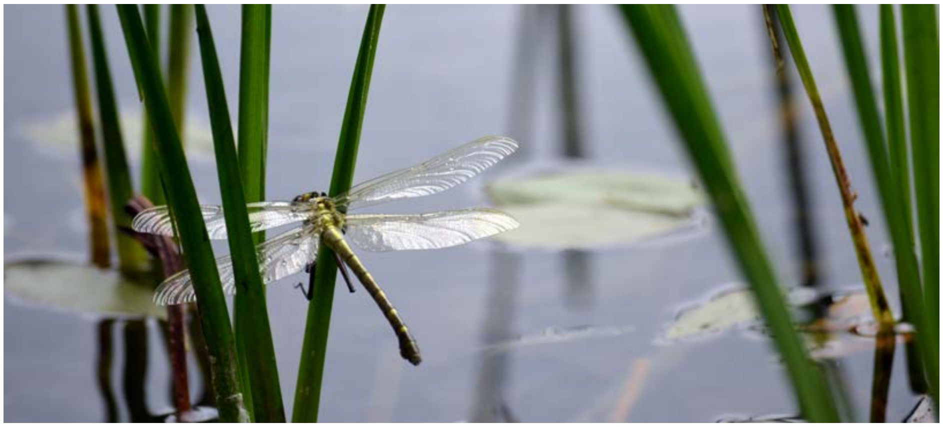

There are well-established methods of calculating the value of such "ecosystem services," and this study makes use of some of them to estimate the total economic value of 13 identified ecosystem services provided by the NCC's green spaces. Similar studies have been carried out in other major urban Canadian areas, including the Ontario Greenbelt, Montreal, the Rouge National Urban Park, and Vancouver's Greater Mainland Area.

The majority of the average monetary values for these ecosystems and the services they provide were derived from 78 peer-reviewed studies published between 1990 and 2016.

An analysis of the NCC's lands identifies five different land cover types:

- Forests (72%);
- Agricultural lands (10%);
- Urban areas (8%);
- Wetlands (5%); and
- And Freshwater (5%).

The majority of the average monetary values for these ecosystems and the services they provide were derived from 78 peer-reviewed studies published between 1990 and 2016. The most valuable, on a per-hectare basis ($59,394/ha/year) are the wetlands — due to the variety and importance of the ecosystem services they provide, as well as their relative scarcity.

The other ecosystems are valued as follows:

- Urban forests ($9,352/ha/year);
- Rural forests ($4,183/ha/year);
- Prairies and grasslands ($3,338/ha/year);
- Croplands ($1,363/ha/year); and
- Freshwater systems ($137/ha/year).

In total, the economic value of all natural and cultivated NCC green space averages $332 million per year, with a range extending from a low of $188 million to a high of $829 million. Considering the net present value of ecosystem services from year to year over a 20-year period, the estimated value of services provided by NCC Green Network works out to $5 billion. Most of this value derives from non-market ecosystem services such as wildlife habitat, waste treatment, disturbance prevention, and global climate regulation.

The NCC's stewardship of these lands and the ecosystem services they provide is invaluable to the Capital Region — and to the country as a whole. The area is also within the Algonquin Nation's ancestral territory.

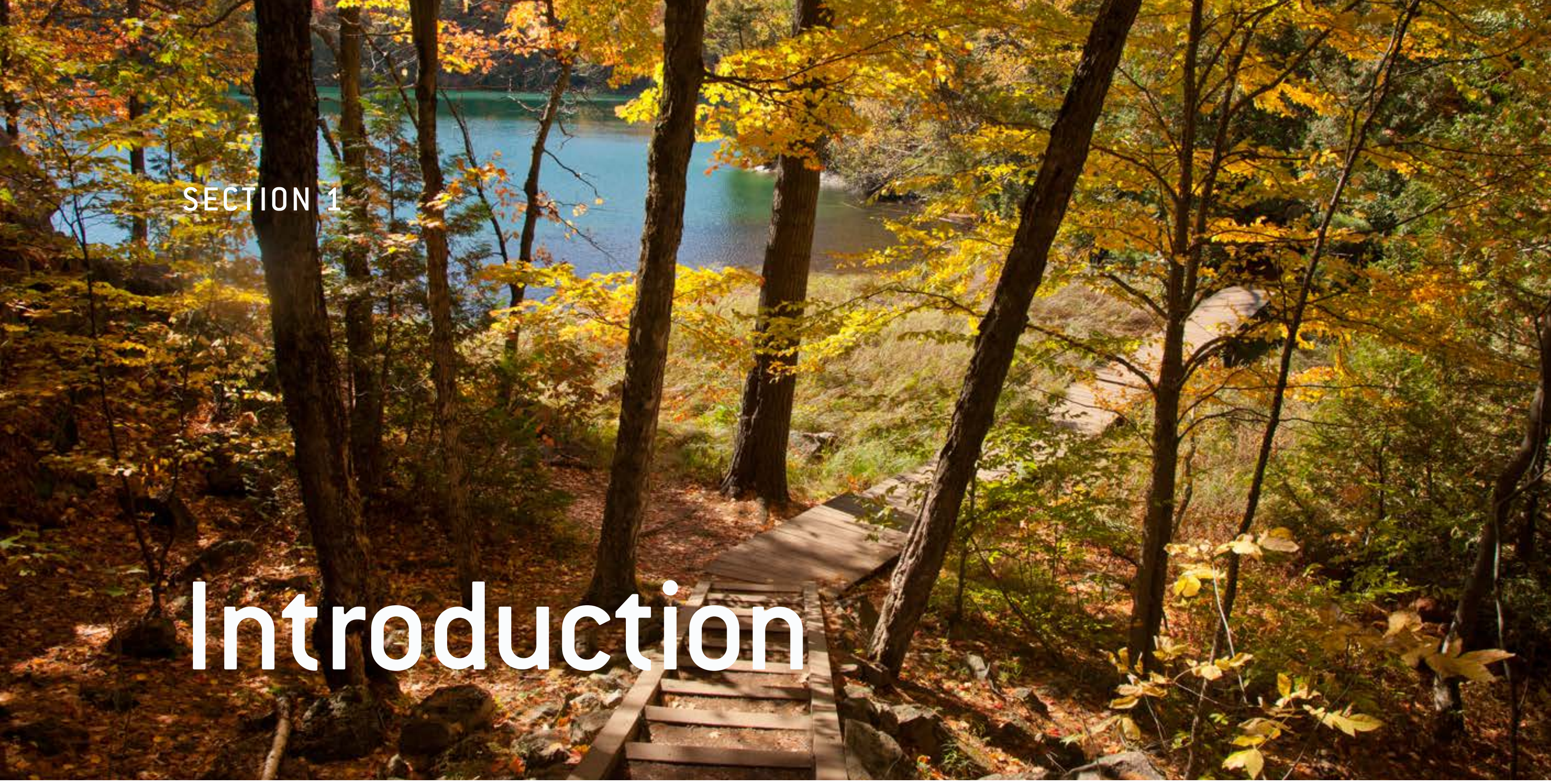

SECTION 1

Introduction

What is natural capital?

Natural capital refers to natural resource stocks, land and ecosystems, which provide an array of resources and flows of ecosystem services necessary for life on earth (Wilson 2010; Statistics Canada 2015). In Canada, our vast landscapes, composed of forests, wetlands, prairies, grasslands, lakes and rivers, provide raw materials and ecosystem services that are necessary for the economic and social well-being of Canadians, but also for humans globally.

Ecosystems provide services that can be benefited from locally or globally. These services include water capture and filtration, climate regulation through carbon storage and sequestration in trees, plants and soils, and improvement of air quality resulting from the absorption of pollutants by trees. Even though ecosystem services are crucial in our lives, since we do not pay directly for their provision, their true value is not accounted for in the market economy. It is estimated that they are worth trillions of dollars per year, contributing to market and non market economies (Costanza et al., 2014). In addition, despite the consensus on the importance of the environment for human well-being, measures of natural capital are not integrated into important economic indicators such as the Gross Domestic Product (GDP), have not been fully evaluated through national natural capital accounts, and are usually not included to land use planning.

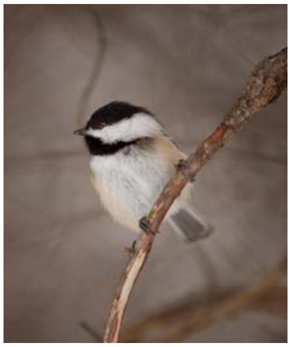

In Canada, our vast landscapes, composed of forests, wetlands, prairies, grasslands, lakes and rivers, provide raw materials and ecosystem services that are necessary for the economic and social well-being of Canadians, but also for humans globally.

ECOSYSTEM GOODS AND SERVICES

Ecosystem services (ES) consist of direct and indirect functions and processes that people and society benefit from economically or that contribute to their quality of life (Breuste et al. 2013). This is a new way of considering ecosystems and natural habitats, i.e., breaking them down into a number of attributes and wellness factors, allowing human life to take form (Boyd and Banzhaf 2007) (See Box 1).

THE IMPORTANCE OF VALUING ECOSYSTEM SERVICES

The degradation and destruction of natural assets and ecosystems are especially noticeable in the urban areas where most people live (Breuste et al. 2013). Despite this degradation, however, urban and peri-urban green spaces (e.g., forests, parks, allotments, and cemeteries) do provide ES at different scales within the urban landscape, including recreation, climate regulation, biodiversity and carbon mitigation (Breuste et al. 2013; Gomez-Baggetun and Barton 2013; Gomez-Baggetun et al. 2013).

These ES have often been neglected in decision-making, land-use planning and management because of a lack of understanding of the processes and mechanisms involved in their creation, and because of the challenges of including them in land-use planning and management (Sandhu and Wratten 2013).

Since urban systems are generally net consumers of ES, surrounding ecosystems in peri-urban, rural or even remote areas, including Greenbelts, contribute significantly to fulfill the cities' daily needs (MEA 2005; Sandhu and Wratten 2013).

We can explain the general trend in ES degradation through large proportions of natural amenities with no economic value in standard markets, which makes their inclusion in the economic system and in many decision-making processes complicated (TEEB 2010; Bateman et al. 2011).

Since urban systems are generally net consumers of ecosystem services, surrounding ecosystems in peri-urban, rural or even remote areas, including Greenbelts, contribute significantly to fulfill the cities' daily needs.

THE MILLENNIUM ECOSYSTEM ASSESSMENT (MEA) is a United Nations initiative that started in 2000, publishing its results in 2005. The objective of this essential scientific work, involving 1,360 scientists from 95 countries, was to analyze the reliance of humans on nature and observe trends in the evolution of natural capital.

To achieve this objective, the MEA proposed an evaluation grid based on the benefits to humans generated by biodiversity and ecosystems. The MEA classifies ecosystem services into four categories:

1. **PROVISIONING SERVICES:** Services that provide consumable natural resources such as food, raw materials, fresh water, medicinal resources, fuel or fibres.
2. **REGULATING SERVICES:** Services that include processes generated from the interaction of living an non-living organisms that create a suitable environment for humans, such as local and global climate regulation, air quality, prevention of extreme events, waste treatment, erosion control, pollination, or biological control.
3. **CULTURAL SERVICES:** Services that provide intangible values that are appropriated by human beings, such as recreation and tourism, mental and physical health, aesthetic appreciation and inspiration for culture, art and design, and spiritual experience.
4. **SUPPORTING SERVICES:** Services that support the basic functions of ecosystems, such as habitat biodiversity, maintenance of genetic diversity, biomass production and nutrient cycling.

According to the UN report's conclusion, our use of natural resources has been unsustainable since the middle of the 20th century. Fifteen of the 24 ecosystem services under study are currently deteriorating or overexploited, including freshwater provisioning, fisheries, water and air purification, global and local climate regulation, and regulation of invasive species.

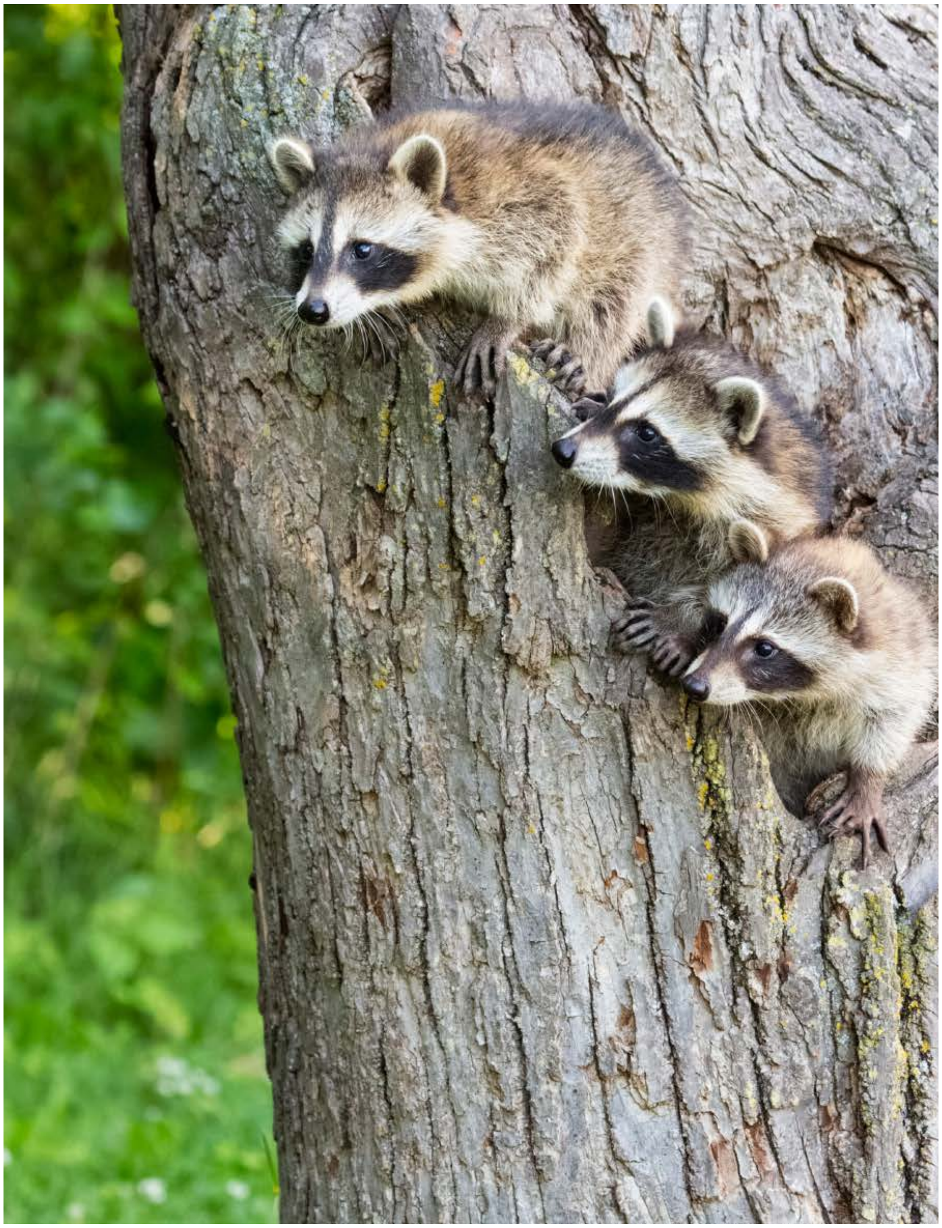

This study measures the market and non-market economic value provided by the National Capital Commission's lands in the National Capital Region. The NCC manages Gatineau Park, the Greenbelt and numerous green spaces referred to as urban lands, collectively referred to as the National Capital Commission's Green Network.

PHOTO: KEN CANNING/ISTOCK

This lack of consideration is particularly reflected in adopting incentives and coping strategies that do not take into account the true value of natural capital. The economic analysis of ES attempts to curb this problem by demonstrating the real contribution of natural capital to the communities' well-being.

This study measures the market and non-market economic value provided by the National Capital Commission's (NCC) lands in the National Capital Region (NCR). The NCC manages Gatineau Park, the Greenbelt and numerous green spaces referred to as urban lands, collectively referred to as the National Capital Commission's Green Network (NCCGN).

It will estimate the value of the natural capital (i.e., NCC lands, forests, wetlands, water systems and agricultural lands) in terms of ecosystem services and the benefits provided by nature to communities.

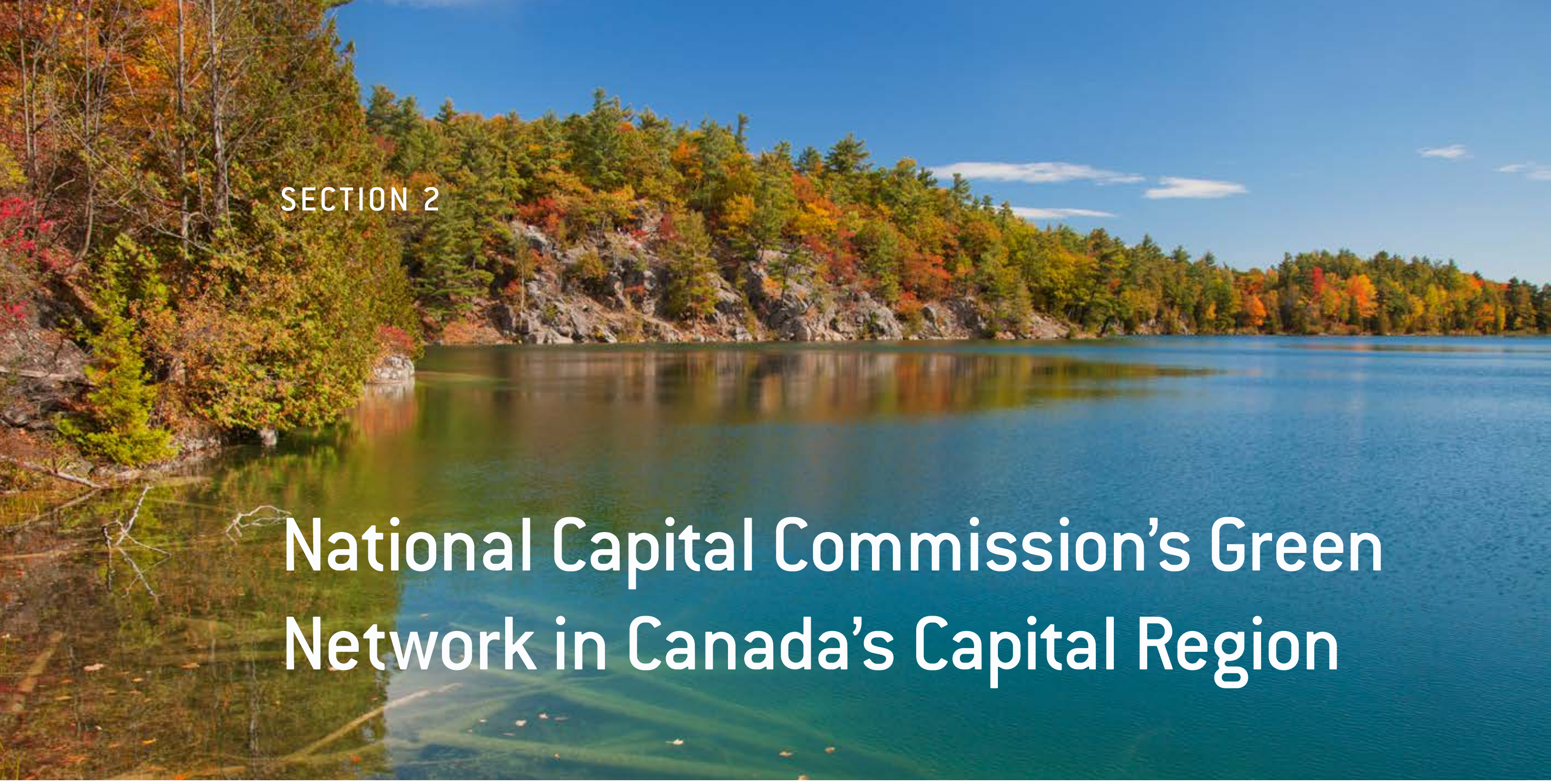

SECTION 2

National Capital Commission's Green Network in Canada's Capital Region

With a mandate reaching back over a century, the National Capital Commission is responsible for preparing plans and for assisting in the development, conservation, and improvement of federal lands in the National Capital Region.

THE NATIONAL CAPITAL COMMISSION is dedicated to building a dynamic, sustainable, inspiring capital that is a source of pride for all Canadians and a legacy for generations to come. With a mandate reaching back over a century, the NCC is responsible for preparing plans and for assisting in the development, conservation, and improvement of federal lands in the National Capital Region. It fulfills this role through the following areas of activity:

- Setting the long-term urban planning direction for federal lands in the NCR;
- Guiding and controlling the use and development of federal lands in the NCR;
- Managing, conserving and protecting NCC assets (including Gatineau Park, the Greenbelt, real property, and other assets such as bridges, pathways and parkways); and
- Maintaining heritage sites within the NCR, such as the official residences and commemorative sites.

The NCC owns over 10% of the lands in the NCR, totalling 473 km^2, and 20% of the lands in the Capital's core. This makes the NCC the region's largest landowner. Gatineau Park, the Greenbelt and numerous green spaces referred to as urban lands form the National Capital Commission's Green Network (NCCGN).

Figure 1 presents the National Capital Commission's Green Network (NCCGN), constituted by Gatineau Park, the Greenbelt and urban lands managed by the NCC.

2.1 The National Capital Region Portrait

The National Capital Region is located at the confluence of the Ottawa, Gatineau, and Rideau Rivers. It includes the cities of Ottawa and Gatineau, as well as Cantley, Chelsea, Clarence-Rockland, Denholm, L'Ange-Gardien, La Pêche, Mayo, Notre-Dame-de-la-Salette, Pontiac, Russell, Val-des-Bois, and Val-des-Monts. According to the 2011 census the Ottawa-Gatineau metropolitan region is home to 1,236,324 people, covers an area of 6,287 km^2, and has a population density of 197 people per km^2 (Statistics Canada 2012).

FIGURE 1. NATIONAL CAPITAL COMMISSION'S GREEN NETWORK

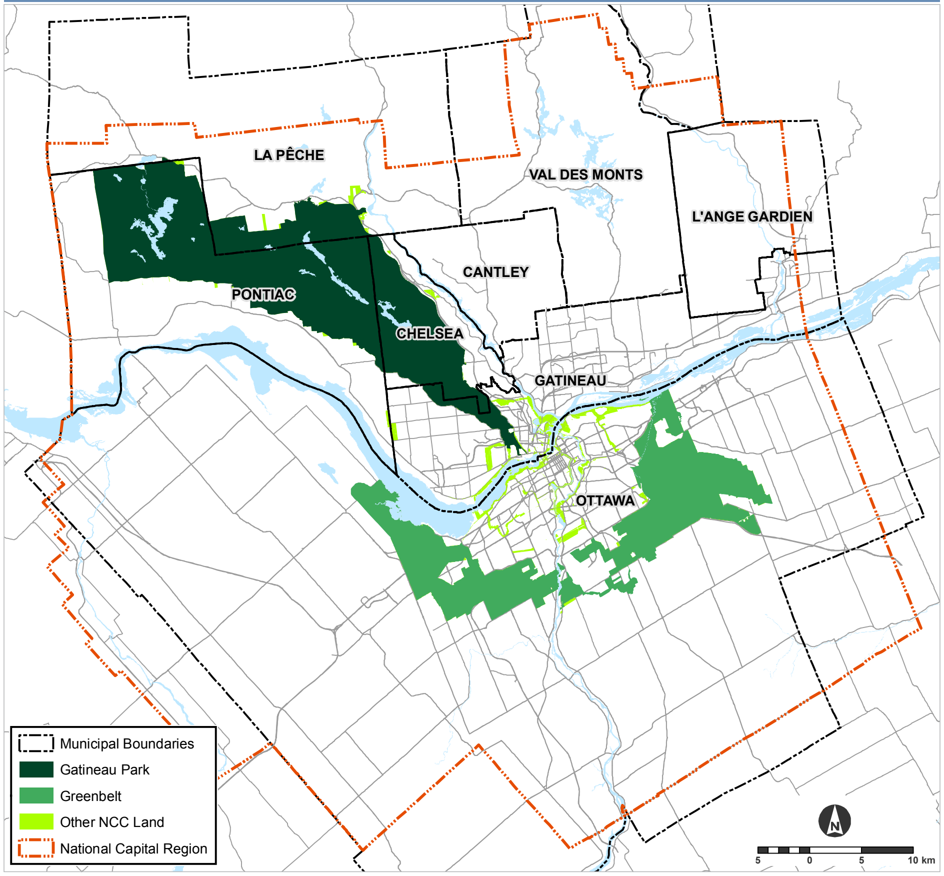

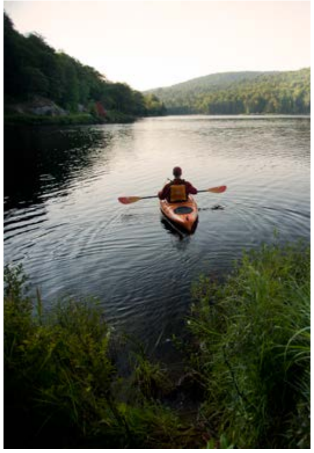

Just 15 minutes from Parliament Hill, Gatineau Park is a prime destination for visitors and residents alike, with more than 2.7 million visits each year. Activities such as hiking, camping, swimming, skiing, and cycling are very popular.

2.2 Gatineau Park

Gatineau Park, located where the Canadian Shield meets the St. Lawrence Lowlands and the Ottawa River meets the Gatineau River in the province of Québec, is home to many unique and diversified ecosystems and heritage features. It covers 36,131 hectares (361 square kilometres) and represents 7.7% of the total NCR area.

Just 15 minutes from Parliament Hill, the Park is a prime destination for visitors and residents alike, with more than 2.7 million visits each year. Activities such as hiking, camping, swimming, skiing, and cycling are very popular.

Cultural and heritage landscapes also make Gatineau Park significant. The Mackenzie King Estate, located in the heart of Gatineau Park, pays tribute to Canada's 10th and longest-serving prime minister, William Lyon Mackenzie King. Mackenzie King bequeathed this 231-hectare estate to Canadians after his death.

Gatineau Park contains approximately 50 lakes and hundreds of ponds. Three-quarters of the neighbouring lands are agricultural.

Gatineau Park's forests are composed of mixed and deciduous forest stands (Del Degan, Massé 2010). These ecosystems and habitats are home to a number of rare plant communities, as well as forests valuable for their local or regional rarity.

The Park is also home to approximately 90 plant species and 60 animal species at risk, including the Wild Leek, the Least Bittern, the Blanding's Turtle, the Four-toed Salamander,

and the Juniper Hairstreak, a butterfly. Overall, the Park is home to more than 1,600 floral species, 54 mammal species, 232 species of birds, 17 amphibian and 11 reptile species, and more than 50 fish species (Del Degan, Massé 2010).

As the National Capital Region's conservation park (Gatineau Park Master Plan 2005), it is a Park priority to encourage discovery and access while minimizing the impact of recreation activities on the natural environment and fragile ecosystems. As such, the NCC's planning activities support the development of sustainable recreational experiences that are respectful of the protected natural environment

2.3 Greenbelt

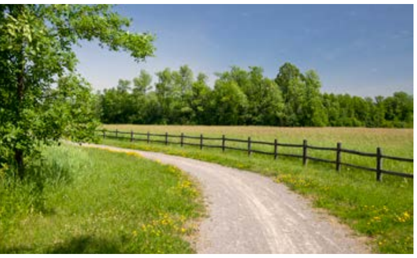

Some valleys in the Greenbelt are sensitive to disturbances and erosion, but they are vital because they support natural systems and agricultural activities.

The Greenbelt, located in the St. Lawrence Lowlands, has been situated entirely within the City of Ottawa since 2001 and the amalgamation of 12 local municipalities. It covers an area of approximately 20,600 hectares, which represents about 5% of the NCR land area (NCC 2013). Almost 900,000 people were living within its boundaries in 2011, and 300,000 people living outside its boundaries in the NCR. There are 12,000 people working within the boundaries of the Greenbelt, and 1,000 people living in it. In addition, 3.5 million visits occur in the Greenbelt annually (NCC 2013).

Within the Greenbelt:

- 75% of the land comprises natural areas, agricultural lands and forests;
- 5% of the land is taken up by roads and is managed by provincial or local governments; and
- the remainder serves recreational, residential, commercial, and institutional uses.

The most frequent special habitats identified throughout the Greenbelt are wetlands and significant forests. Provincially designated wetlands are found in Shirley's Bay, Mer Bleue Bog, Lester Wetland and Stony Swamp. Mer Bleue is especially important as it represents a northern bog ecosystem generally found in arctic climates, and it has been designated as an internationally significant wetland under the Ramsar Convention (NCC 2013).

Overall, the Greenbelt is divided into seven sectors: Shirley's Bay, Stony Swamp, Southern Farm & Pinhey Forest, International Airport, Pine Grove, Mer Bleue, and Green's Creek.

The Greenbelt's underground resources are composed of sand plains, clay plains, organic soils, sand dunes, and limestone bedrock located on the surface (NCC 2013). The main discontinuity in the landscape is caused by creek systems, the Rideau River and the rocky regions to the west of the Greenbelt. Some valleys in the Greenbelt are sensitive to disturbances and erosion, but they are vital because they support natural systems and agricultural activities.

Old-growth and mature forests are rare in the region due to agriculture and urbanization in and around Canada's Capital. Consequently, the small pockets of residual old-growth forests that remain are of significant value, since they can serve as seed source for late-succession tree species. Natural forests and plantations account for approximately 18% of the Greenbelt. The plantations, which represent 800 hectares out of 3,500, have been established on old agricultural lands characterized by mixed forests with maples and oaks. More than 60 species at risk live in the Greenbelt, including the Butternut, the Bobolink and the Snapping Turtle.

The second and most recent Greenbelt Master Plan was released in 2013. It looks forward to 2067 (NCC 2013) and highlights the four objectives for the Greenbelt:

- Improve biodiversity, health and resilience by adding significant natural areas adjacent to the Greenbelt boundaries and enhancing connectivity;
- Re-designate former rural lands for sustainable agriculture;
- Enhance visual resources, recreational opportunities, and connectivity between the region's various pathway systems; and
- Retain facilities requiring isolation, but encourage status quo in size and, where possible, encourage the reduction of land footprint that supports built infrastructures.

2.4 Urban Lands

The NCC and the federal government own or manage nearly 4,500 hectares of lands of capital importance in Ottawa and Gatineau. The Capital's urban lands are divided in two parts:

- Downtown Ottawa and Gatineau (known as the core area lands);
- And urban lands surrounding the heart of the Capital (known as the Urban Lands).

The Urban Lands are located in Ottawa and Gatineau and cover an area of 22.5 km^2. About 20% of Urban Lands are conservation areas and 22% are natural areas. The remaining 56% of Urban Lands areas are comprised of agricultural land, recreational areas and various other types of sites such as parkways, parking lots, picnic areas and trails. Urban Lands are managed to meet recreo-touristic development as well as to provide access to natural areas for all Canadians and visitors who want to enjoy nature in the NCR.

The main natural areas in the Urban Lands are the shorelines and islands of the Ottawa, Rideau and Gatineau Rivers, Mud Lake, Leamy Lake Park, Rockcliffe Park, Moore Farm, and the natural corridors (e.g., Champlain and Philemon-Wright Corridors, McCarthy Woods, and Pinecrest Creek). Recreation and tourism activities are very popular in the Urban Lands.

Wetlands are important in an urban landscape because they provide valuable ecosystem services related to water quality, flood control, and water retention. They are also important habitats for wildlife. A Provincially Significant Wetland is found in Mud Lake. Smaller wetlands were also present in Leamy Lake, Carlington Woods, and Voyageurs Corridor. Other special habitats that have been identified include a heron nesting site and a fish spawning area in Leamy Lake and islands that serve as bird refuges in Champlain Bridge and Lemieux Islands and Voyageurs Corridor. More than 70 species at risk inhabit the Urban Lands, including the Butternut, the Western Chorus Frog, and the Snapping Turtle.

The Capital Urban Lands Plan (2015) is a land-use plan to guide the planning, protection and development of federal urban lands in the Capital. The plan applies to urban lands inside the Greenbelt on the Ontario side of the Ottawa River, and within the urban perimeter on the Quebec side.

The Capital Urban Lands Plan supports the NCC's vision for an inspiring and dynamic capital. It focuses on highlighting the natural and picturesque qualities of the Capital, offering a welcoming and memorable experience for visitors, and contributing to the character and livability of the Capital for those who reside here.

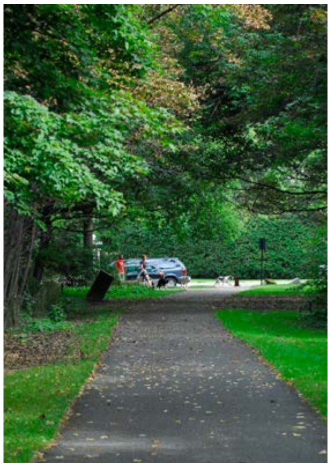

The Capital Urban Lands Plan focuses on highlighting the natural and picturesque qualities of the Capital, offering a welcoming and memorable experience for visitors, and contributing to the character and livability of the Capital for those who reside here.

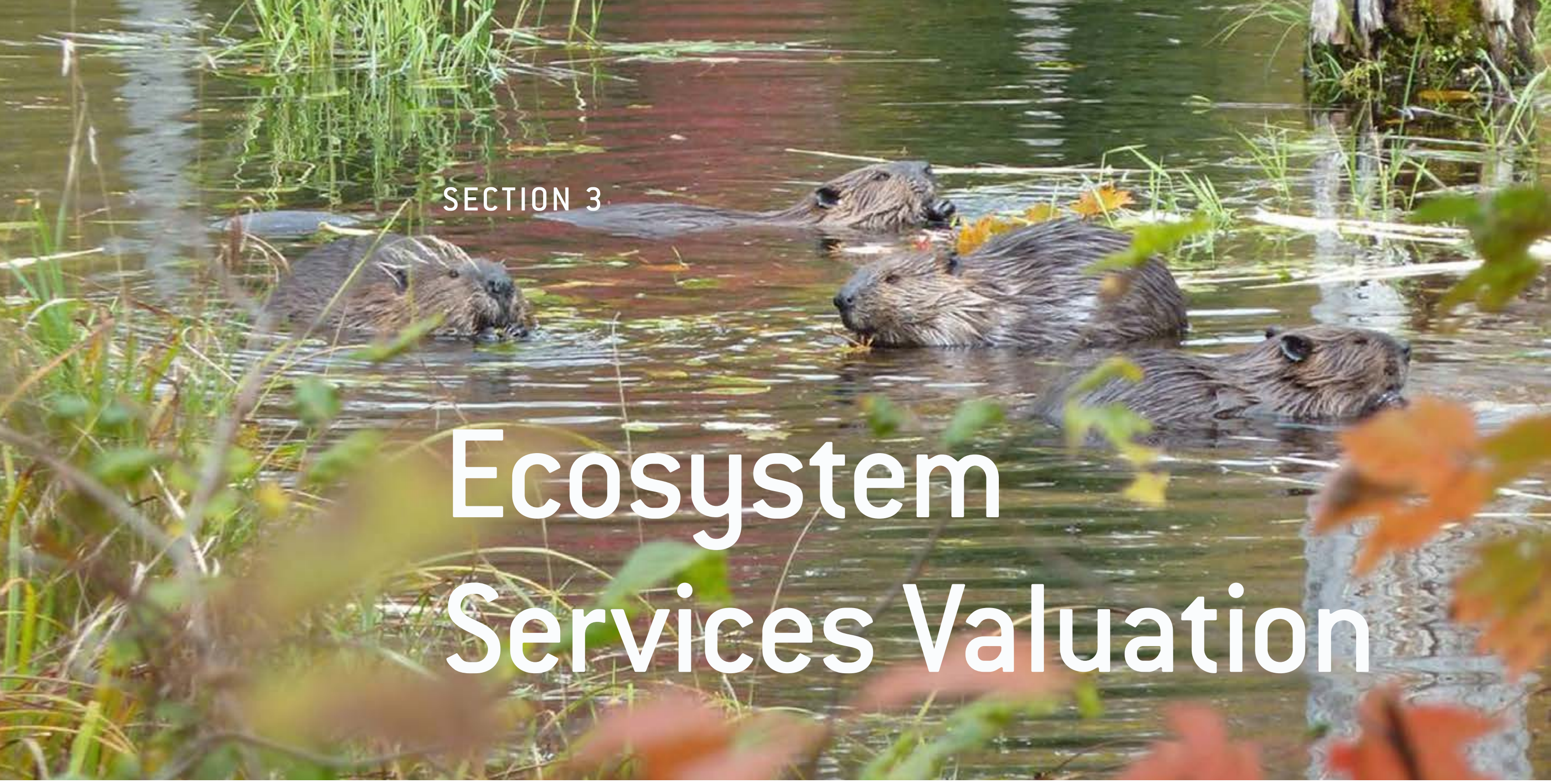

SECTION 3

Ecosystem Services Valuation

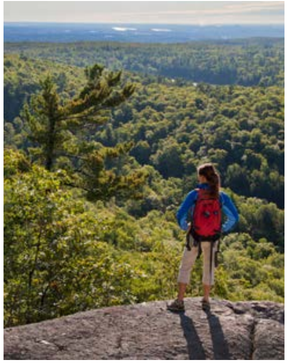

Despite an increasing interest in the economic valuation of biodiversity and ecosystems, this approach remains complex and raises a number of technical and ethical issues.

TOP PHOTO COURTESY ANNE-MARIE THÉRRIEN

BOTTOM PHOTO: NCC

3.1 Historical and Conceptual Perspectives

THE METHODS USED for the economic valuation of natural amenities were developed for the first time in the 1950s, in particular with the work of Ciriacy-Wantrup (1952) on American forests, but their inclusion in the political and scientific agenda has mainly taken place since the 1980s. A number of events have made this approach unavoidable in conservation initiatives. For example, these techniques have been used to estimate the compensation to residents following the Exxon Valdez spill in 1989. They have also been used in Costanza et al.'s flagship publication (1997) on the global economic value of ecosystems, and in the Millennium Ecosystem Assessment (2005). More recently, the economic valuation of biodiversity has been recognized as an important conservation strategy by the Secretariat of the Convention on Biological Diversity, a United Nations entity, through the Nagoya Protocol of 2010.

Despite an increasing interest in the economic valuation of biodiversity and ecosystems, this approach remains complex and raises a number of technical and ethical issues. One of the main reasons that explain the difficulty of including ES economic valuation in economic processes is essentially related to the nature of goods and services. Standard goods and services exchanged on the market — such as direct consumption goods like food, or services provided to individuals like financial services — are generally of a private nature, meaning that they are owned by individuals

In contrast, some goods or services are more difficult to conceptualize from this perspective of individual ownership, and these are generally public goods such as water, air, or access to landscapes. In general, these public goods can be used by more than one individual at a time, and no one can be prevented from using them. In economic theory, this refers to the principles of rivalry and exclusion (Samuelson 1954). This results in the incapacity of traditional markets to include public goods.

In 1968, Garret Hardin stated that free access to natural resources would result in their overexploitation, in what he called the "tragedy of the commons." Even though significant research — in particular that of Elinor Ostrom, winner of the Nobel Prize of Economics in 2009 — has demonstrated that this is not always the case, over-exploitation of natural resources remains a real trend. A number of public goods available on a global scale demonstrate the effects of this overexploitation. For example, the free access to the atmosphere for the release of greenhouse gases is causing climate change, and a lack of regulation in the fishing industry and the high seas has led to the collapse of a large part of the fish stocks (Pauly and Zeller 2016).

The problem with private appropriation of public goods makes it difficult to include them in economic systems, leading to their non-representation in these settings. Even though a public good or service is essential to life, it usually has no economic value. In this context, many authors and institutions suggest that evaluating the economic value of public goods can reflect their contribution to economic activities and promote decision-making that is coherent with the interdependence of humans and nature (MEA 2005; TEEB 2010).

This intention to give an economic value to nature raises a number of questions and ethical issues: Given that human life is dependent on nature, how realistic is it to affix a monetary value to nature? Can living species have an economic value? If so, why would some species have a greater value than others? Would the monetization of nature result in a commodification of living organisms by economic systems and lead to environmental and social justice issues?

To answer these fundamental questions, the scientific literature suggests that a clear distinction should be made between "ordinary biodiversity" and "extraordinary biodiversity" (Centre d'analyse stratégique 2009). The latter refers to elements of nature, genes, species or ecosystems that are collectively represented. Here we can think of emblematic species such as the polar bear or the snowy owl, or to natural sites valued by communities such as national parks. As part of economic valuation efforts, it is considered futile and even simplistic to assign an economic value to elements of our natural heritage that are already valued for reasons other than economic, such as reasons of culture, heritage, education, or spirituality.

"Ordinary biodiversity" refers to elements of natural capital that are not valued socially. We can think of species or ecosystems that are unknown or fundamental processes of ecosystems such as primary production of biomass or nutrient cycling, which are more intangible. These elements are essential to supporting life on Earth, but they suffer from an inadequate valorisation by human systems, which leads to their deterioration and overexploitation. We generally refer to this "ordinary biodiversity" when talking about ecosystem services that we want to value economically, with the intent to promote better environmental management practices for the benefit of communities.

To better represent the economic value of ES, we refer to the notion of total economic value. This notion includes all the values from nature, market or not. Figure 2 highlights the different types of values that form total economic value. Direct use value represents the direct appropriation value of a good or service, often found on economic markets. One could think of the value of wood or food, for which it is possible to spend a sum of money in exchange for ownership.

Indirect-use value represents the value that affects human well-being but is not found on traditional markets. Here, we can think of regulating services such as the prevention of extreme events, pollination or climate regulation. Option values represent future-use values of natural resources. These possess a value, market or nonmarket, which reflects their potential use in

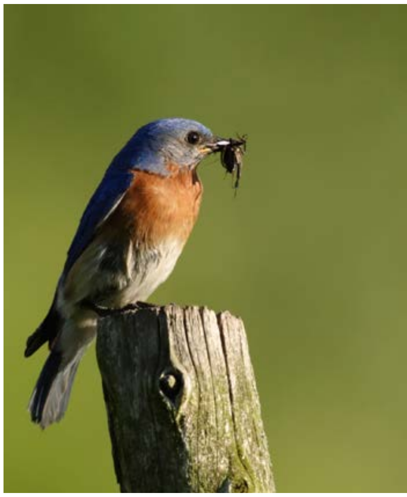

To better represent the economic value of ecosystem services, we refer to the notion of total economic value. This notion includes all the values from nature, market or not.

PHOTO: PAUL TESSIER/ISTOCK

the future. Non-use values (heritage values and existence value) are difficult to quantify, but have a real impact on human well-being. These subscribe to the notion of transgenerational respect and equity.

FIGURE 2. BREAKDOWN OF THE CONCEPT OF TOTAL ECONOMIC VALUE

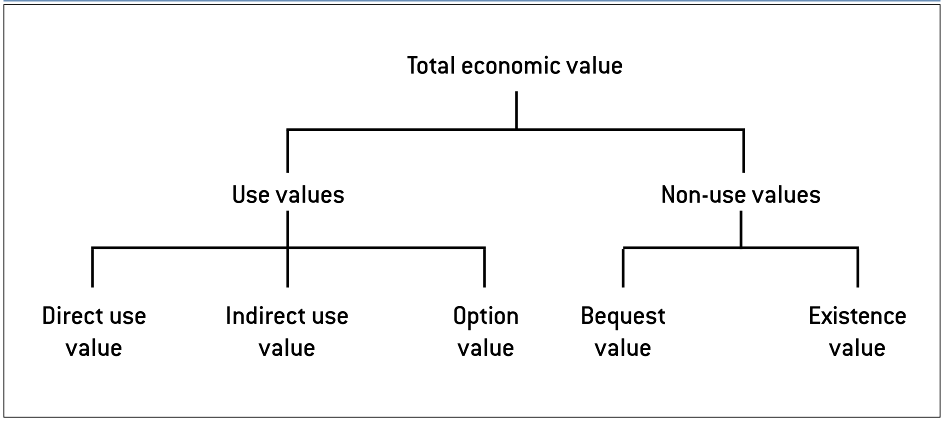

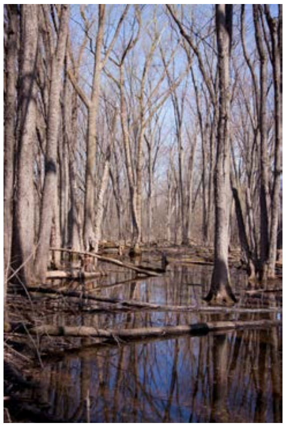

3.2 Tools and Methods of Economic Valuation

Many tools and methods of ES economic valuation have been developed over the past decades. These methods can be grouped into five categories: market pricing, cost-based approach, indirect markets, simulated markets, and benefit transfer. All of these methods have their strengths and weaknesses, and their use depends on the characteristics of the ecosystems under study, the beneficiaries of ES, and the geographic scale of the study.

In the case of methods based on market prices, we can determine the value of tradeable ES, such as food, by looking at their value on the market. This valuation method is especially suitable for provisioning ES and it estimates the value of products or ES that are sold or bought on the market. In this study, we are using this approach to determine the value of agricultural products and recreational activities.

Cost-based approaches estimate the value of ES based on the cost of replacing ES generated by an ecosystem or the damage cost that would be induced by their loss. Let's think about the role of wetlands in flood control. It is possible to evaluate the value of this service by calculating damages induced by floods if the services weren't provided, or even how much it would cost to replace these services by grey infrastructure. In this study, we use this approach to estimate the value of the carbon sequestration and storage.

Methods related to indirect markets, such as transport costs and hedonic pricing, refer to existing markets to allow a secondary economic analysis of ES. This principle is based on the idea that the value of some services can be internalized in marketed goods. The two examples most often used are real estate markets and expenses related to tourism. In the first case, the value of houses can be influenced by elements rooted in natural capital, the aesthetics of a landscape or water quality, for example. In the second case, we use money spent by individuals to benefit from nature through multiple tourist activities to determine the value of ES generated by a site, like a natural park.

Let's think about the role of wetlands in flood control. It is possible to evaluate the value of this service by calculating damages induced by floods if the services weren't provided, or even how much it would cost to replace these services by grey infrastructure.

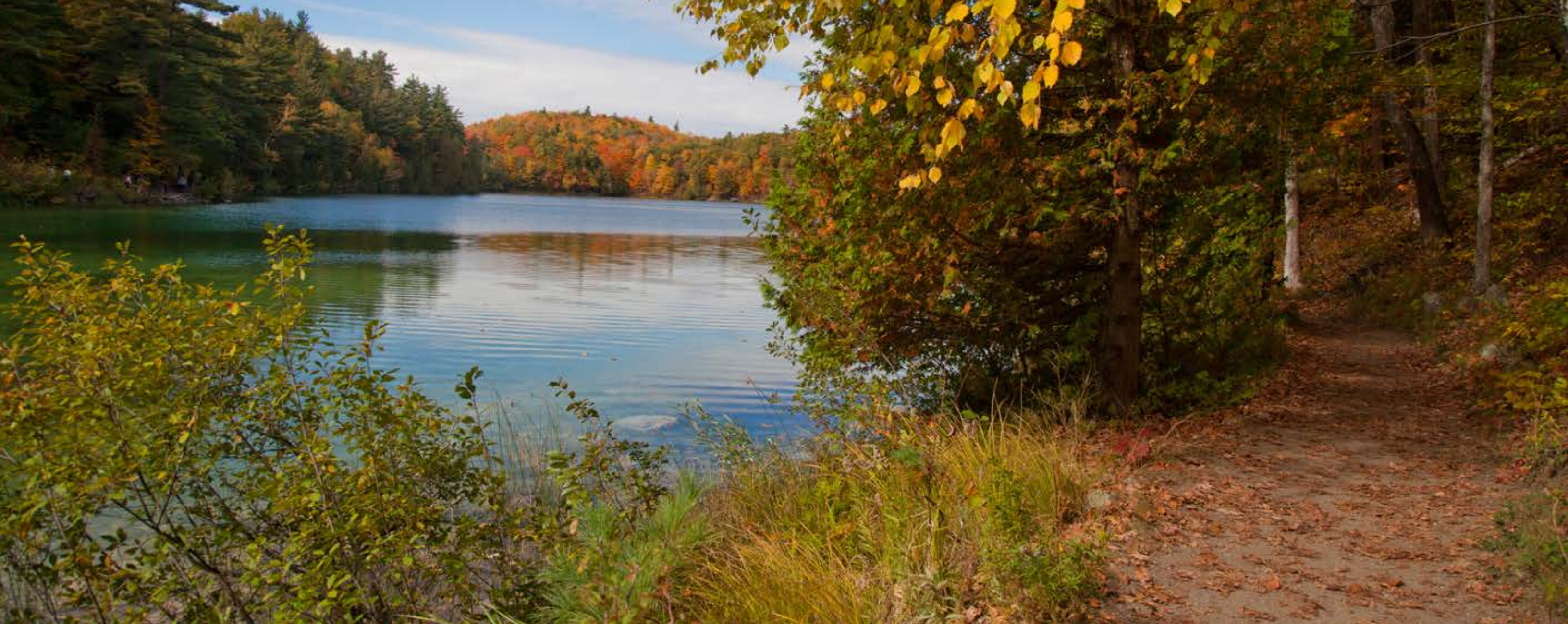

Simulated market-based methods have been developed to evaluate ES for which there are no markets, direct or indirect. In these cases, we create a hypothetical market and, through surveying techniques, measure how much individuals would be willing to pay for specific ES. For example, He et al. (2016) asked respondents how much they would be willing to pay to double the size of wetlands in Quebec to increase water quality, protection against floods, and preserve biodiversity.

Finally, the benefit transfer method is a collection of methods based on an analysis of secondary data. For technical or economic reasons, it is often impossible to perform an analysis for specific ES or specific sites. In these cases, we can use studies that have been conducted elsewhere and transfer the results to another site. For the study on NCCGN, we have used two of these methods to evaluate a bundle of ES (See Methodology section for details).

Many tools and methods of ES economic valuation have been developed over the past decades. These methods can be grouped into five categories: market pricing, cost-based approach, indirect markets, simulated markets, and benefit transfer.

3.3 Examples of Ecosystem Services Valuation of Urban Natural Capital in Canada

For many years, ES economic valuation has been applied to many natural and semi-natural ecosystems, using various methods. In Canada, many studies have been undertaken on the value of ES generated by a variety of ecosystems, from wetlands (Pattison et al. 2011; Lantz et al. 2013; He et al. 2016) to forests (Anielsky and Wilson 2005; Wilson 2008; Dupras et al. 2015) and aquatic environments (Poder et al. 2015).

Among these exercises, some have looked at the value of green infrastructure in urban environments. The TD Canada Trust recently measured the contribution of trees on urban residents' quality of life. TD Canada Trust specialists estimated that each tree in Toronto has a value of $700, and a cumulative value of $7 billion for the entire urban forest (TD Economics 2014a). This value is based on the impact of trees on the control of stormwater runoff, air quality, carbon sequestration and energy savings. A similar approach was used to estimate the value of urban forests in Vancouver ($35 billion), Montreal ($4.5 billion) and Halifax ($11.5 billion) (TD Economics 2014b).

In 2008, Wilson demonstrated, by evaluating 16 ES, that the Ontario Greenbelt, surrounding Toronto, has a non-market value of $2.7 billion, i.e. a mean value of $3,500 per hectare per

year. The main ES, in economic terms, are habitat for wildlife, climate regulation, pollination and the treatment of pollutants.

Using a similar approach, two studies conducted on the Greenbelt and green infrastructure in the Greater Montreal area have determined that non-market ES have an annual value between $2.2 billion and $4.3 billion (Dupras et al. 2013; Dupras et al. 2015). The ES with the highest value are habitat for biodiversity, control of air quality, recreational activities and tourism, and water provisioning. Dupras and Alam (2015) have demonstrated, by looking at the evolution of land use cover of Montreal's metropolitan region since the 1960s, that urban sprawl had an annual cost of more than $235 million in term of ES lost.

3.4 Ecosystem Services Valuation in Decision-Making

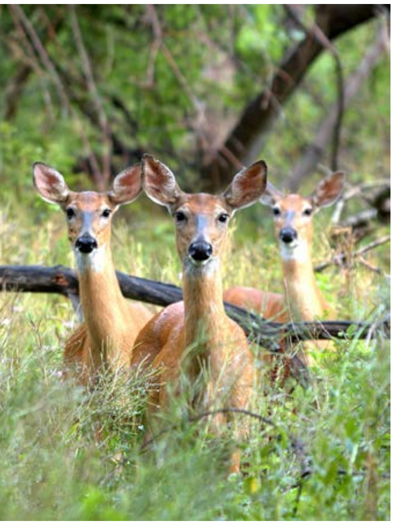

In Canada, initiatives abound to increase knowledge of natural capital and ES values that has the potential to influence decisions.

PHOTO: LIGHTWRITER1949/ISTOCK

Despite the growing number of tools and studies undertaken on the value of ES, the use of natural capital and ES values in decision-making is still low (Daily et al. 2009; TEEB 2012; Laurans et al. 2013; Guerry et al. 2015). As stated in the article by Guerry et al. (2015), "Despite this progress, incorporation of natural capital and ecosystem services information into diverse decisions remains the exception, not the rule" (p. 7352). In their 2009 article, Daily et al. argued that the relatively recent understanding and utilisation of ES valuation is partly responsible of this situation. However, a recent research has highlighted that other factors might be involved, including the conflict over the short versus long-term horizon inherent in decision-making, valuation methods, and the relegation of natural capital and ES to the realm of Environment rather than Finance (Guerry et al. 2015).

A growing number of countries are incorporating measures of natural capital and ES into policy and management (Guerry et al. 2015). For example, China has announced that it will track natural capital and ES through the "gross ecosystem product" metric, and that it will be reported alongside GDP. South Africa is using ES planning to inform decisions in water management, allocation processes, poverty alleviation and land-use planning. Belize is using ES values to identify the appropriate balance between tourism, fisheries, and coastal protection in its coastal zone management. The United States is incorporating ES information in decision-making and natural damage assessment, and the InterAmerican Development Bank is now integrating ES into infrastructure investments.

In Canada, initiatives abound to increase knowledge of natural capital and ES values that have the potential to influence decisions. In this regard, we can note Statistics Canada's Measuring Ecosystems Goods and Services initiative, a project that propelled research on ecosystem accounting and the quantification of ecosystem goods and services, and the Environmental Valuation Reference Inventory (EVRI). EVRI is an extensive database of environmental valuation research that is maintained collaboratively by a number of countries. Some programs, namely Payments for ecosystems services, have also been implemented to use fiscal incentives and stimulate the production of ES in the agricultural, forest, and water sectors. At the turn of the 2010s, more than 40 programs promoting the production of ES were in effect in Canada (Kenny et al. 2011). Market-based instruments and new markets are also being developed for traditional non-market ES. The most significant example is the implementation of carbon markets in several Canadian provinces.

SECTION 4

Methodology

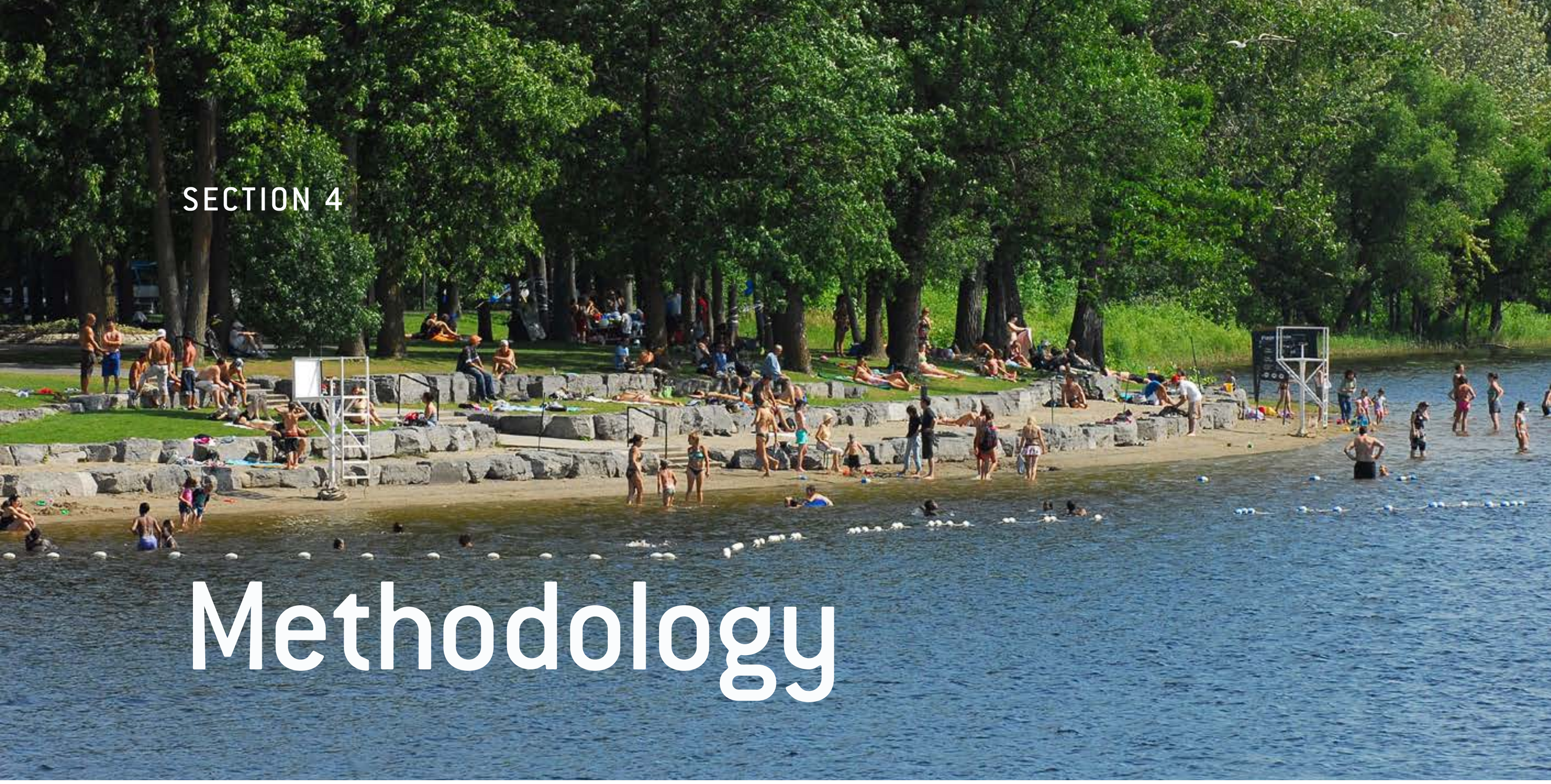

THE METHODOLOGY FOR THIS STUDY is a combination of economic valuation and spatial analysis. On the one hand, it serves to analyse the study area's territorial composition geographically and associate a certain amount of ES to each type of land cover classes and, on the other hand, it serves to identify economic indicators that are representative of ES associated with ecosystems under study.

This method is often used in this area of research. For example, 72 studies of this kind have been published in the scientific literature since the beginning of the 2000s. This methodological approach has been described in a scientific article by Troy and Wilson (2006). These authors suggest a method in five steps:

1. Spatial designation of the study area;
2. Classification and mapping of land use cover;
3. Economic valuation of ES;
4. Calculation of total ES and breakdown by land use cover classes; and
5. Spatial analysis of ES valuation by relevant management geographies.

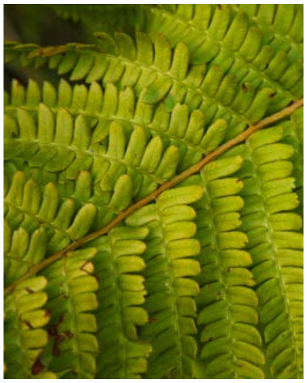

Seventy-two studies of this kind have been published in the scientific literature since the beginning of the 2000s.

4.1 Spatial Designation of the Study Area

As described earlier, the area under study are the green spaces owned by the National Capital Commission (NCCGN) including Gatineau Park, the Greenbelt, and the Urban Lands of the National Capital Commission. The objective of analysing this territory is to echo the planning and development strategic plans for these territories and of the region. This is done so that the concept of ES and their contribution to the well-being of communities in the region can reflect the economic, social and environmental importance of the green network in Canada's Capital and can be integrated into discussions on the current and future planning of the territory.

4.2 Classification and Mapping of Land Use Cover

After consulting many Geographic Information System (GIS) databases, we have selected Agriculture and Agri-Food Canada's (AAFC) 2014 land cover inventory to go forward with land-use analysis. We selected this database because it allows for a consistent coverage of the study area at a fine resolution (30 m^2), and it includes many categories of land use (28 categories for the study area). Like all analysis produced using satellite imagery, however, the AAFC land use cover analysis has a certain level of uncertainty. According to the metadata available for this analysis, this layer has a level of precision of about 85%. Despite the fact that the area under study is too large to carry out a manual correction to increase the accuracy of the GIS analysis, a randomized photo-interpretation approach has allowed to confirm that the layer was in general precise and that it is suitable for subsequent analysis.

Using ArcGIS software, we used the AAFC's land use cover analysis. We also made the distinction between urban and rural areas using Statistics Canada's classification. The distinction refers to demographic characteristics, such as population size, density, and distance to important agglomerations (Statistics Canada 2011). Generally, urban space is defined as an area (i.e. municipality) with a population of at least 1000 and a density of 400 or more people per square kilometre. All other areas are considered rural.

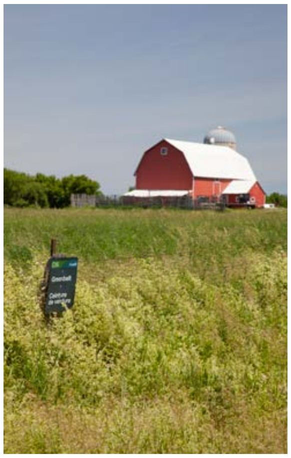

Generally, urban space is defined as an area (i.e. municipality) with a population of at least 1000 and a density of 400 or more people per square kilometre. All other areas are considered rural.

The second phase of this step consisted of selecting the ES produced by each of the ecosystems under study. Many classification systems exist and aim to identify and characterise ES. The first were proposed by Daily (1997), Costanza et al. (1997), and De Groot et al. (2002). It is, however, the Millennium Ecosystem Assessment (2005) that laid the foundation for a classification system of 17 ES that was largely adopted. Since then, other initiatives have made regional and global classifications. As such, it is worth mentioning the TEEB initiative that proposed a classification of 22 ecosystem services divided into four categories: provisioning, regulation, habitat, and cultural. Haines-Young and Potschin (2008) have suggested a more elaborate classification known as CICES (Common International Classification of Ecosystem Goods and Services). This classification suggests a partition of nine classes regrouped into three themes (provisioning, regulation and maintenance, cultural). It excludes the MEA's "supporting services" and the habitat services/functions from the TEEB (2010) and De Groot et al. (2002).

Based on the most standardized classifications (i.e. MEA, TEEB, CICES) on a literature review of the valuation exercises of ES undertaken in Quebec and in Ontario (e.g. Wilson, 2008; Dupras et al. 2015; Dupras et Alam 2015) and the workings of our own database, we have selected 13 ES that have appeared relevant for the NCCGN.

PROVISIONING SERVICES:

1. Agricultural products: Food and forage from annual and perennial crops

REGULATING SERVICES:

2. Global climate regulation: Carbon storage and sequestration in biomass from a greenhouse gas reduction perspective
3. Air quality: Capacity of an ecosystem to filter air pollutants

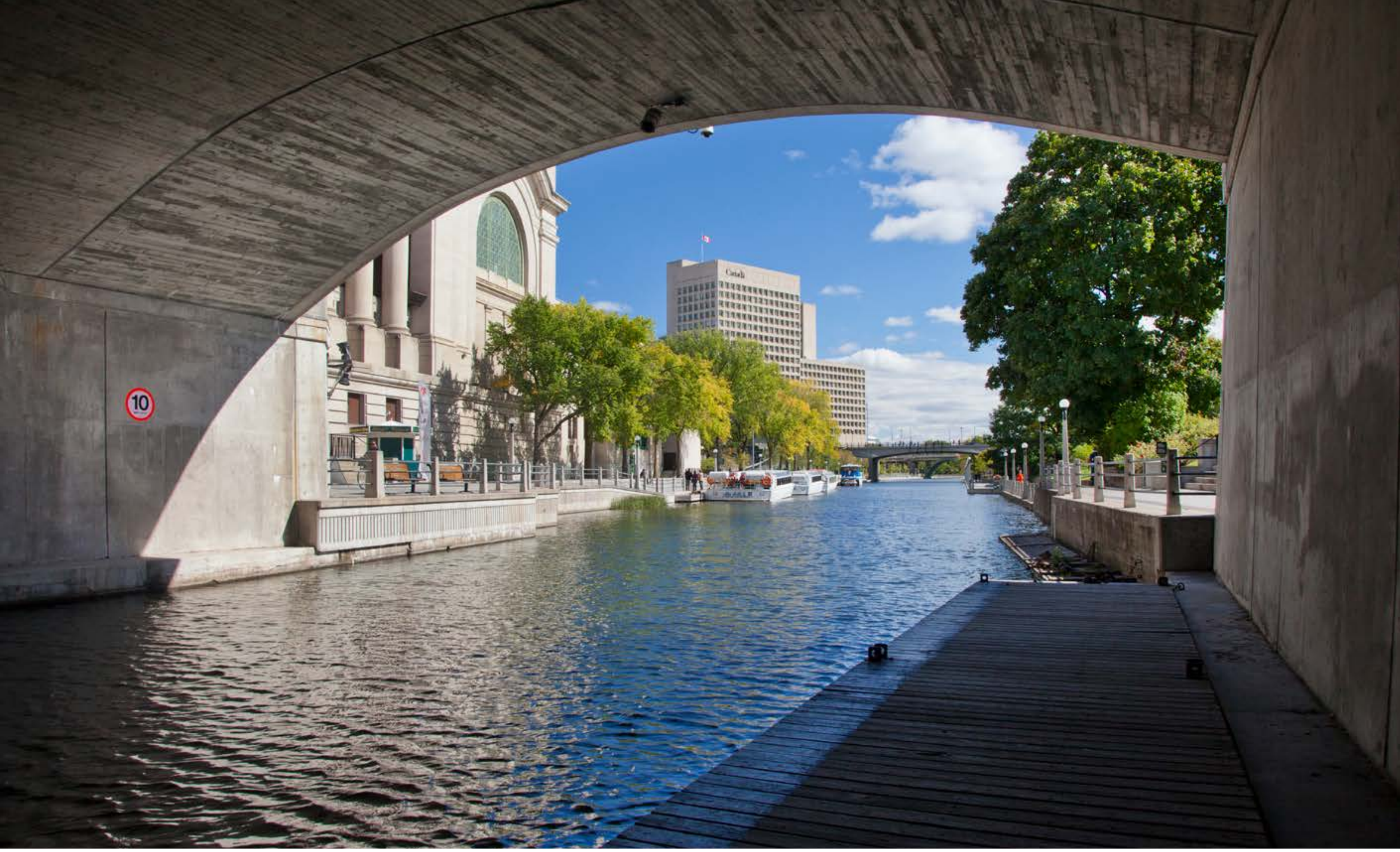

4. Water provisioning: Regulation and flood-control capacity in a perspective of water provisioning for communities
5. Waste treatment: Capacity of an ecosystem to filter and metabolize pollutants
6. Erosion control: Capacity of an ecosystem to preserve the soil's structure and to prevent erosion from water and wind
7. Pollination: Pollination of plants by insects and animals, allowing for the quantitative and qualitative production of food or other products
8. Habitat for biodiversity: Capacity of an ecosystem to offer a suitable habitat for biodiversity at large
9. Disturbance prevention: In this study, this service refers mainly to the prevention of floods
10. Pest management: Regulation of diseases and pests that have the ability to impact agricultural production or human health
11. Nutrient cycle: Soil formation through the interaction between biotic and abiotic matter.

We selected Agriculture and Agri-Food Canada's (AAFC) 2014 land cover inventory to go forward with land use analysis because it allows for a consistent coverage of the study area at a fine resolution (30 m^2), and it includes many categories of land use (28 categories for the study area).

CULTURAL SERVICES:

12. Aesthetics (of landscapes): Appreciation of the beauty of a natural asset, landscape, ecosystem
13. Recreational activities and tourism: Leisure and tourism related to ecosystems

4.3 Ecosystem Services Valuation

To perform the analysis of the ES mentioned above, we have selected three methodological approaches. In the case of provisioning services, i.e. agricultural products and recreation, we have used the market pricing method. For non-market ES, we've used the benefit transfer approach by using transfer methods with adjustment and a meta-analysis, and the replacement costs method.

4.3.1 MARKET PRICING METHOD

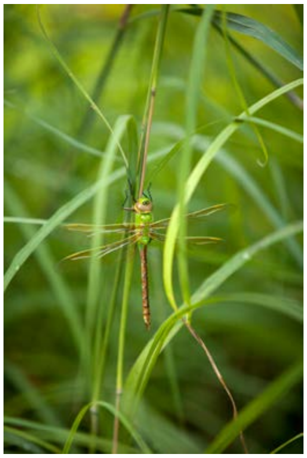

To analyse the economic contribution of agricultural systems in terms of food products, we have to compute the value of economic rents, which is equal to the revenues generated from the sale of agricultural products minus the total expenditures incurred to produce them. To do this, we have identified the crops grown in the NCCGN and searched for the value of these products and the related expenditures in a number of databases such as the Financière agricole du Québec, the Ontario Ministry of Agriculture, Food and Rural Affairs, and the Quebec Reference Center for Agriculture and Agri-food (*Centre de référence en agriculture et agroalimentaire du Québec* –CRAAQ). For the recreation service, we used the value of user fees charged by Gatineau Park for services like skiing, snowshoeing, beaches, and summer camping; otherwise admission to the Park is free.

For non-market ecosystem services, we've used the benefit transfer approach by using transfer methods with adjustment and a meta-analysis, and the replacement costs method.

4.3.2 REPLACEMENT COSTS METHOD

We used this method to assess the value of the climate regulation service. This service is evaluated in our study, as in most of the scientific literature, in terms of carbon sequestration and storage. To do this, we used the value of the social cost of carbon (SCC), as determined by Environment and Climate Change Canada. The SCC is a monetary measure of the expected damage of global climate change resulting from the issuance of an additional ton of carbon dioxide (CO_2) into the atmosphere during a given year.

4.3.3 BENEFIT TRANSFER WITH ADJUSTMENT

This approach consists of using economic valuations of ES undertaken at other sites and transferring the values to the target site while adjusting these values so that they represent the characteristics of the target site. To do this, we have created a database that represents the land classification found in the NCCGN and the ES that they produce. We first started from a database that was created for the analysis of the Montreal Greenbelt and then conducted an exhaustive analysis of the scientific literature to complete the database using recent and representative studies of the NCCGN. Existing databases such as the Environmental Valuation Reference Inventory (www.evri.ca) and the Ecosystem Services Valuation Database

(www.fsd.nl/esp/80763/5/0/50) were explored, but we also performed specific research in specialised search engines (e.g. EconLit, Francis).

The level of accuracy of this method depends on the degree of conformance between the sites used for the transfer and the target site. In this regard, it is important to ensure there is an equality of income between the sites and there are similarities between the biophysical environments. Two general criteria were used to select the studies whose results would be transferred. First, an ecological criterion ensured that we considered only the studies undertaken in temperate ecosystems where the ecosystems were similar to those found in the NCCGN. Second, a socio-economic criterion allowed for selecting studies undertaken in countries and regions with socio-economic and demographic conditions comparable to the NCCGN. Since the value of ES is often related to their contribution to the communities' well-being, the characteristics of these communities are key explanatory factors. In the end, the selected studies for the value transfer were mainly taken from projects conducted in North America and Western Europe.

To perform a correct transfer value, an adjustment was made. First, the values were converted in Canadian dollars using purchasing power parity conversion tables. This conversion method is more precise than using only exchange rates, because it reflects the purchasing power of each currency. Then, the values in Canadian dollars were corrected according to inflation rates so that they could be expressed in 2015 dollars.

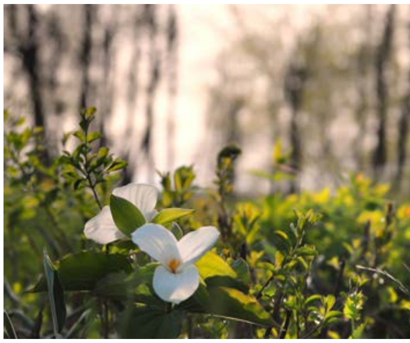

The social cost of carbon (SCC) is a monetary measure of the expected damage of global climate change resulting from the issuance of an additional ton of carbon dioxide (CO_2) into the atmosphere during a given year.

PHOTO COURTESY JANA KRIZ

4.3.4 BENEFIT TRANSFER WITH META-ANALYSIS

The meta-analysis is a statistical method that is used to summarize information coming from many independent studies to infer a value on a target site. By using a large number of studies in its statistical model, it reduces transfer errors which makes it more precise and rigorous than simple transfer values and adjusted transfer values.

In this approach, we don't directly transfer the value associated to an ES, but rather the explanatory factors associated to this value (i.e. by transferring the coefficients of the good or service's characteristics). Generally, these explanatory factors represent socio-economic and environmental characteristics of the ecosystems under study. For example, if the meta-analysis identified significant explanatory factors (i.e. coefficients) of the value, such as the average income of the population (a1), the size of the site (a2), the ES provided (a3), and the population size (a4), by integrating the values of the target site, we could find the value of the subject under study. Then, the equation could be written as:

$$V = a1W + a2X + a3Y + a4Z + ei$$

The value of the ecosystem under study could be measured by integrating equation data into the population's income (W), the size of the site under study (X), the ES provided by the ecosystems (Y), and the population size at the target site (Z).

This approach is more precise than the other benefit transfer methods, but it is also more complex to undertake and the meta-analysis models are few and/or privately owned. In this study, we have used a meta-analysis model developed by He et al. (2015) that allows us to determine the value of four ES (commercial products, disturbance protection, waste treatment, and habitat for biodiversity) generated by wetlands. In their model, the authors explain the value of these services based on a series of explanatory factors described in Table 1.

To include the NCCGN's data to this model, we have performed a documentary and spatial analysis. The documentary analysis has allowed the identification of values corresponding to categories of ES provided by wetlands, to wetland types, and to socio-economic characteristics. For the spatial analysis, essential to the identification of values for the categories of geographical characteristics, we have based our methodology on the one put together by He et al. (2015). Using ArcGIS's software, we first divided the NCR in sub-regions of 50 km^2. In each of these sub-regions, we have measured the total size of wetlands and the percentage of agricultural and urban lands surrounding each individual wetland.

TABLE 1. DESCRIPTION OF EXPLANATORY VARIABLES (ADAPTED FROM HE ET AL. 2015)

Category	Variable	Coefficient Summary Description	
**Wetlands' Ecosystem Services**	Biodiversity Habitat	1.584	The wetland holds a particular biodiversity and natural habitat
	Waste Treatment	0.893	The wetland removes pollutants and filtrates water
	Disturbance Prevention	1.485	The wetland provides its management role of flood control and retention
	Commercial Activities	1.899	The wetland allows commercial activities that are either commercial fishing, hunting or ducks breeding
**Type of Wetland**	Manmade	2.505	The wetland is not natural; it is built by man
	Isolate	-0.856	The wetland is isolate
	Complex	0.868	The wetland is in a complex
**Geographic Characteristics**	Agriculture	-0.019	The percentage (%) of the territory in agriculture within a radius of 10 km around the wetland
	Urban	0.007	The percentage (%) of the territory in urban area within a radius of 10 km around the wetland
	In wetlands' size	-0.560	Logarithm of the size of wetlands in hectares
**Socio-economic Characteristics**	In GDP per capita	1.291	Logarithm of GDP per capita in PPP 2003 USD
**Type of Value**	Marginal	1.484	Economic value of wetland was determined for a marginal change
	Median	3.004	The economic value of wetland reported in the primary study is a median
	Stated preferences	1.087	The study is either based on contingent valuation or choice experiment methods
**Constant**		-3.668	

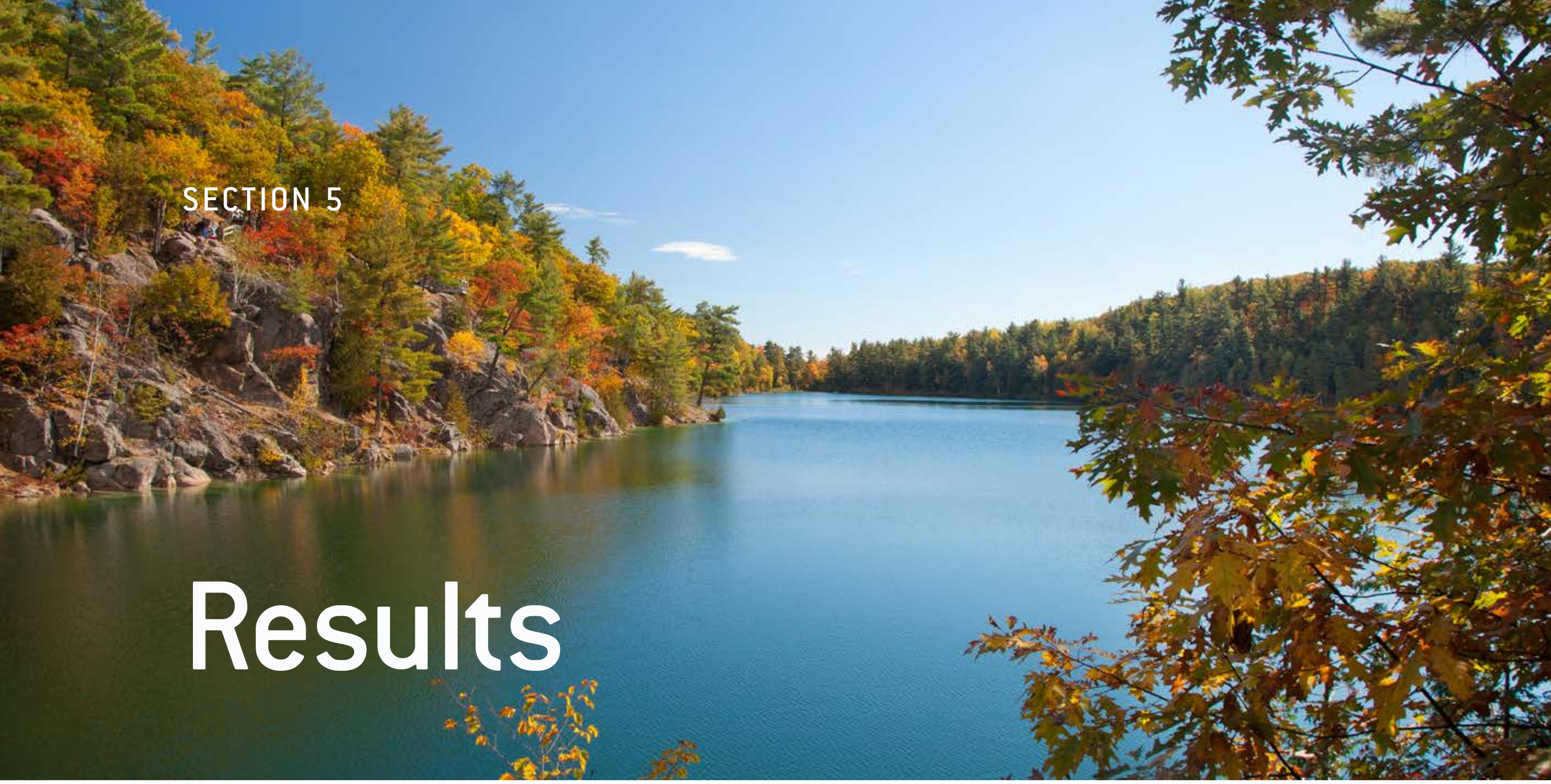

SECTION 5

Results

5.1 Spatial Analysis

Spatial analysis shows that the NCCGN, the total land area managed by the NCC in the National Capital Region, represents more than 55,000 hectares, shared between the Greenbelt, Gatineau Park, and Urban Lands. The two most important land uses are forests (72%) and agricultural lands (10%). Urbanized areas represent 8% of the territory, mostly in the Urban Lands, but some of which are found in the Greenbelt and Gatineau Park (See figure 3). Freshwater systems and wetlands combined represent close to 10% of the area. More details about specific spatial analyses are given in Table 2 and the following sections. Table 3 presents the land cover analysis of Canada's Capital region. The entire National Capital Region represents more than 516,000 ha of land. Forests represent the most important land use cover with 49% of the territory, followed by Agriculture (27%), and Urban areas (14%). Finally, freshwater systems and wetlands combined represent about 10% of the territory. The most important differences between land managed by the NCC and the entire National Capital Region is that forested areas are over represented in NCC lands, and that agricultural and urban areas are a less represented in NCC lands. This difference can be explained in part by the mandate of the NCC of assisting in the development, conservation, and improvement of federal lands in the NCR.

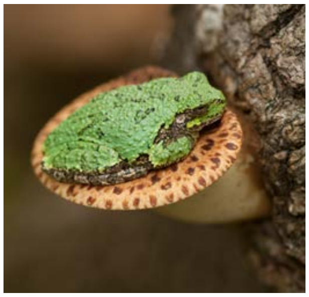

The database that we built contains 149 monetary estimates from 78 different studies. These studies were published between 1990 and 2016, and deal with sites mainly located in the United States, Canada, and European countries.

FIGURE 3. LAND USE COVER OF THE NATIONAL CAPITAL COMMISSION'S GREEN NETWORK

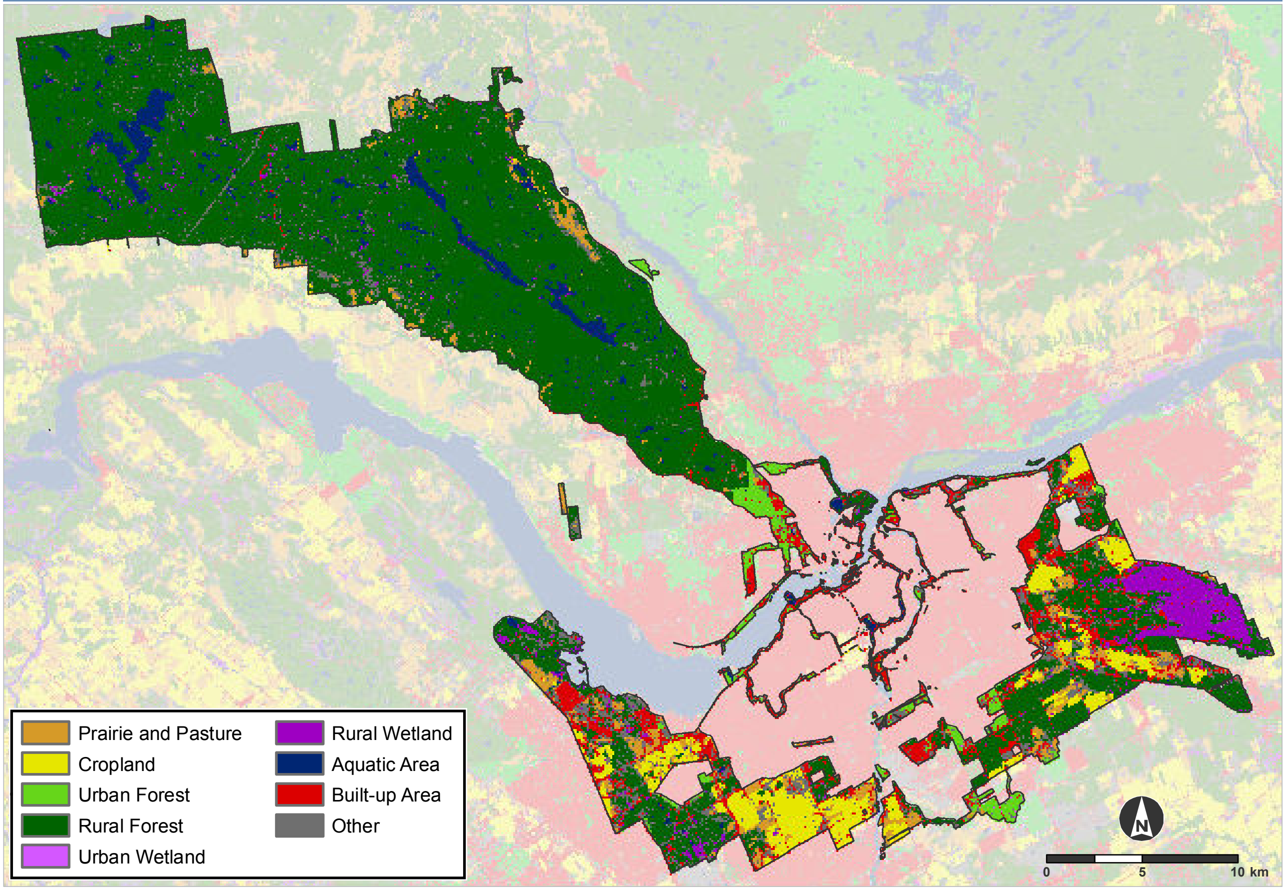

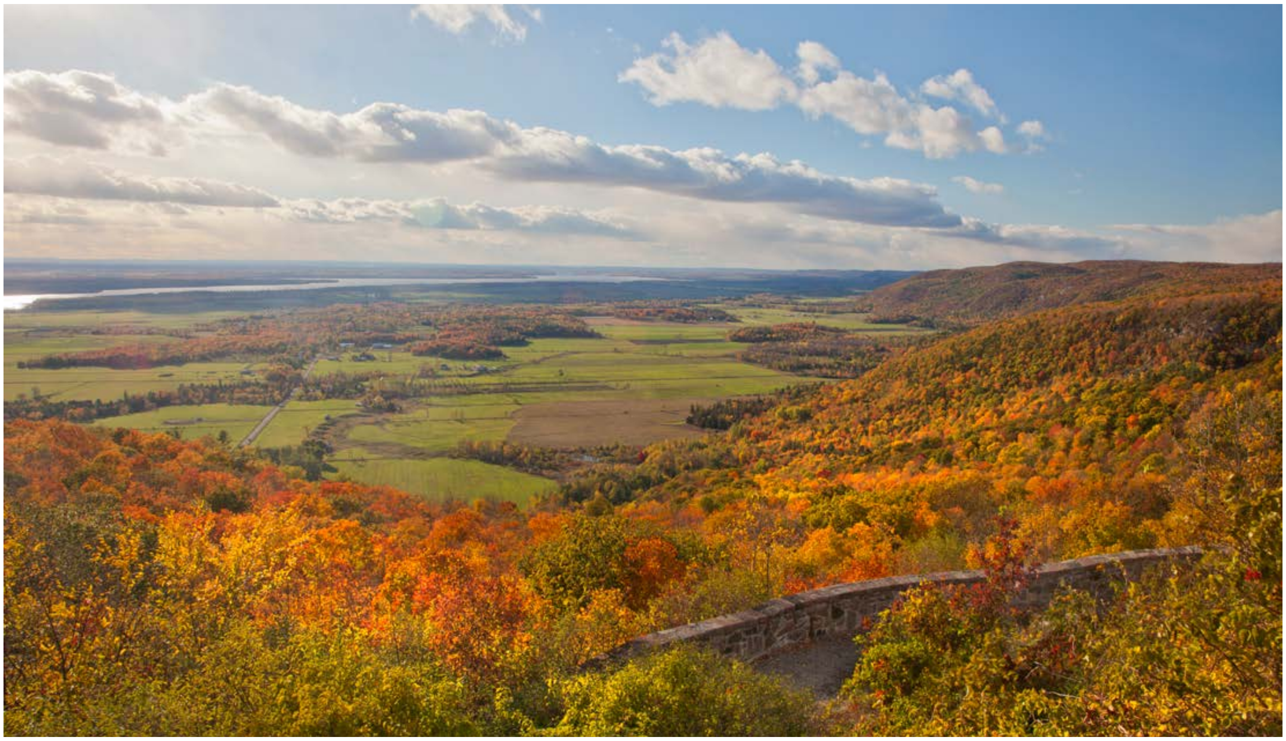

TABLE 2. LAND USE COVER OF THE NATIONAL CAPITAL COMMISSION'S GREEN NETWORK		
Land use cover class	Area (ha)	Area (%)
Rural Wetlands	2417	4.4
Urban Wetlands	36	0.1
Rural Forests (Broadleaf)	15,208	27.6
Rural Forests (Mixedwood)	21,660	39.3
Rural Forests (Coniferous)	1260	2.3
Urban Forests (Broadleaf)	511	0.9
Urban Forests (Mixedwood)	983	1.8
Urban Forets (Coniferous)	58	0.1
Agriculture (Barley)	104	0.2
Agriculture (Beans)	3	0.0
Agriculture (Berries)	17	0.0
Agriculture (Corn)	769	1.4
Agriculture (Fallow)	1	0.0
Agriculture (Oats)	3	0.0
Agriculture (Other grains)	27	0.0
Agriculture (Pasture/forages)	2320	4.2
Agriculture (Soybeans)	2362	4.3
Agriculture (Wheat)	20	0.0
Freshwater Systems	2722	4.9
Urban/developed	4642	8.4
**TOTAL**	**55,123**	**100.0**

TABLE 3. LAND USE COVER OF THE NATIONAL CAPITAL REGION		
Land use cover class	Area (ha)	Area (%)
Rural Wetlands	16,317	3.2
Urban Wetlands	1477	0.3
Rural Forests (Broadleaf)	80,207	15.5
Rural Forests (Mixedwood)	126,852	24.5
Rural Forests (Coniferous)	17,104	3.3
Urban Forests (Broadleaf)	11,417	2.2
Urban Forests (Mixedwood)	18,548	3.6
Urban Forets (Coniferous)	1242	0.2
Agriculture (Barley)	1200	0.2
Agriculture (Berries)	90	0.0
Agriculture (Corn)	32,416	6.3
Agriculture (Fallow)	96	0.0
Agriculture (Oats)	363	0.1
Agriculture (Other grains)	617	0.1
Agriculture (Pasture/forages)	60,972	11.8
Agriculture (Soybeans)	42,150	8.1
Agriculture (Wheat)	1156	0.2
Freshwater Systems	32,064	6.2
Urban/developed	73,051	14.1
**TOTAL**	517,339	100.0

5.2 Ecosystem Services Valuation

The database that we built contains 149 monetary estimates from 78 different studies. These studies were published between 1990 and 2016, and deal with sites mainly located in the United States, Canada, and European countries (i.e., Italy, France, Finland, Sweden, Austria, United Kingdom, Ireland). All reported studies were published in the scientific literature and were subject to a peer review process before publication. The publications considered were primary studies, that is to say not using secondary data as in the benefits transfer approaches. We used this database to assess most non-market ES.

5.3 Forests and Woodlands

Ecosystem services provided by forests and woodlands vary based on their location (rural, urban, or peri-urban) and composition (broadleaf forest, coniferous forest, or mixed forest). The NCR is located in the cool temperate forest (Wilson 2008). According to data on the forest cover, 4% of the forested land cover is located in urban area, and 96% is located in rural area. Additionally, 57% of the forest cover is mixed; 40% has a majority of broadleaves, and 3% has a majority of conifers. Figure 4 presents the distribution and the relative abundance of each type of forest in the NCCGN.

Climate change mitigation has taken a leading role in the political and scientific agendas over the past two decades. In general, solutions are targeting the reduction of greenhouse gases, specifically CO_2, which is the main gas involved in global warming. In terms of **climate regulation**, the scientific literature mostly refers to carbon storage and sequestration by ecosystems. Carbon storage refers to the total amount of carbon stored in an ecosystem at a given time, whereas carbon sequestration represents the annual quantity of carbon stored in an ecosystem minus the leakages into the atmosphere caused by respiration, disturbances, and decomposition. In other words, carbon storage is a reserve of carbon and carbon sequestration is an annual flux.

To assign an economic value to the carbon, we used the Social Cost of Carbon (SCC). The SCC is a monetary measure of the expected damage to the global climate change resulting

Forests, trees, and woodlands capture CO_2, but they also intercept other pollutants that impact local air quality, such as carbon monoxide, sulfur dioxide, and suspended particles.

FIGURE 4. FORESTED AREAS OF THE NATIONAL CAPITAL COMMISSION'S GREEN NETWORK

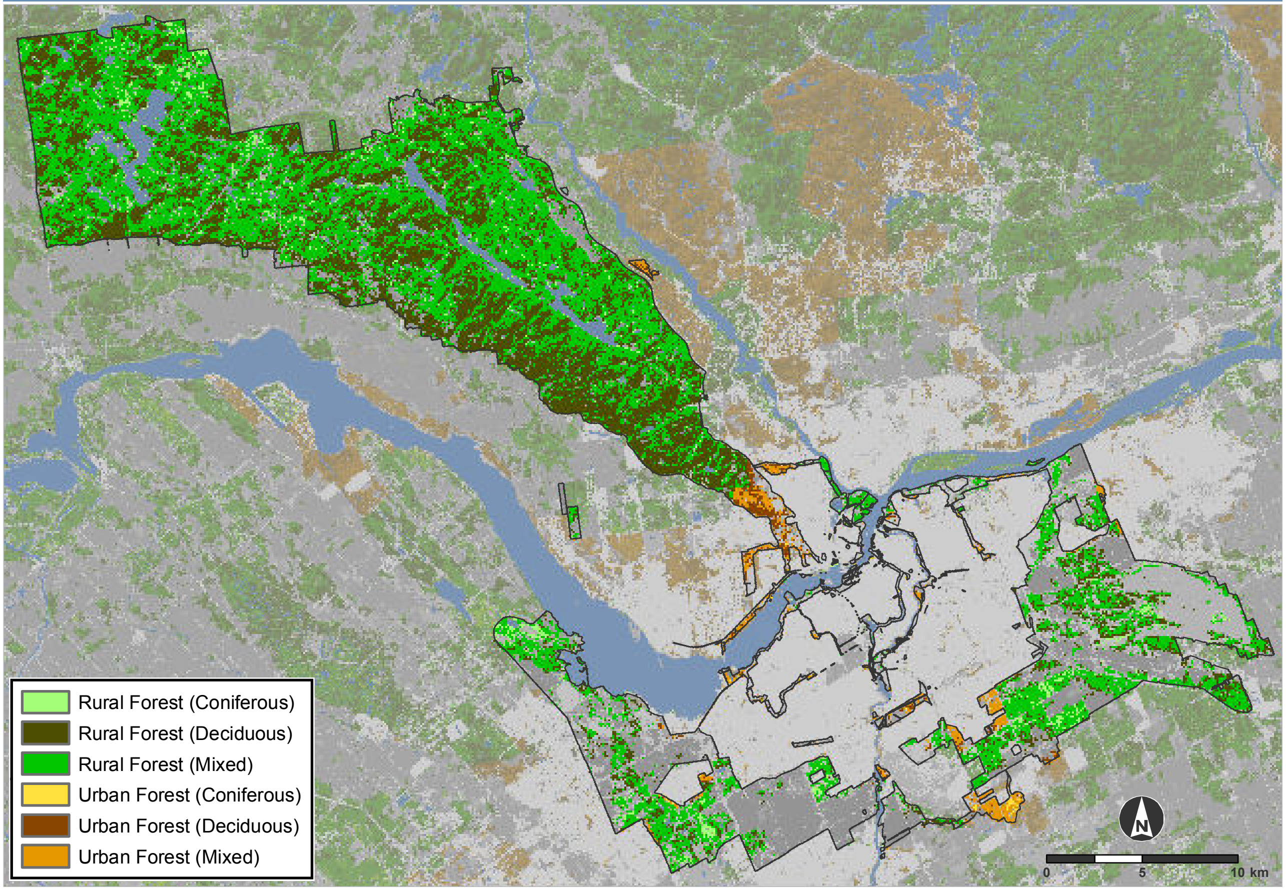

from the issuance of an additional ton of carbon dioxide (CO_2) into the atmosphere during a given year. Environment and Climate Change Canada recommends its use to assess changes in CO_2 emissions in the context of economic analyses, including cost-benefit analyses, that can inform policy makers of the economic impacts of climate change mitigation public policies. In a recent report, Environment and Climate Change Canada (2016) estimates the SCC value to be $43/tonne of CO_2 equivalent.

To evaluate carbon sequestration, we used the average value of recorded carbon sequestration rates by Environment Canada between 1990 and 2009 (Dupras et al. 2013). This quantity corresponds to 1.93 tCO_2/ha/year, which is valued at $83/ha/year.

To evaluate carbon storage, we used a long-term study by Kurz and Apps (1999). In their analysis, they evaluated the carbon fluxes in the Canadian forest sector over a 70-year period. The authors estimated that the stock of carbon stored in the cool temperate forests, found in the NCCGN, was equal to 220 tonnes/ha. Nearly nine million tonnes of carbon are stored in the NCCGN forests.

To transpose the total annual value of carbon, we distributed carbon stocks over 50 years. We used a discount rate of 3% to estimate the annual value, which is the rate recommended by Environment and Climate Change Canada (2016) to analyse the economic value of the carbon. Considering these parameters, the carbon storage value of forests in the NCCGN is $158/ha/year. The value estimated for climate regulation is then $241/ha/year.

Forests, trees, and woodlands capture CO_2, but they also intercept other pollutants that impact local air quality, such as carbon monoxide, sulfur dioxide, and suspended particles. These pollutants contribute to atmospheric pollution, episodes of smog, and health problems such as asthma. The presence of trees in an urban environment is especially crucial to offset atmospheric pollution, provide clean air and reduce health costs. Table 4 provides information on the effects of urban and rural forests on **air quality**. The air cleaning capacities of trees were translated in economic terms using the I-tree software value multipliers and described by Hirabayashi (2014), resulting in a value of $10/ha/year for rural forests and $554 /ha/year for urban forests.

Pollination is a naturally occurring process undertaken by wind, insects, and animals, which results in plant fertilization. Pollination is a critical step in the sexual reproduction of flowering plants, and it results in the growth of the plants and their evolution. To estimate the value of the service provided by pollinators, we've hypothesized, based on a study of Rands and Whitney (2011), that the area of influence of pollinators is 1 km around agricultural lands

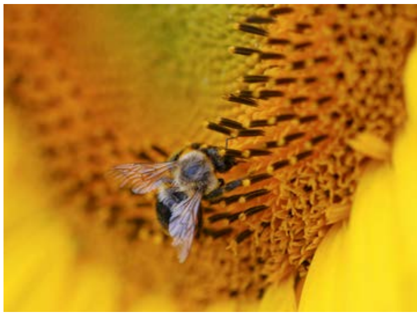

Pollination is a naturally occurring process undertaken by wind, insects, and animals, which results in plant fertilization. Pollination is a critical step in the sexual reproduction of flowering plants, and it results in the growth of the plants and their evolution.

PHOTO COURTESY JULIETTE GIANNESINI (ZHU/FLICKR)

TABLE 4. MULTIPLIERS DERIVED FROM THE UNITED STATES' TOTAL VALUES

Polluant	Removal Multiplier (kg/ha/yr)		Value Multiplier ($/ha/yr)	
	Urban	Rural	Urban	Rural
CO	1.27	1.00	1.7	0.02
NO_2	7.00	5.45	3.1	0.04
O_3	54.04	54.93	154.1	2.6
PM_{10}	15.34	18.51	97.3	2.1
$PM_{2,5}$	2.76	2.66	297.4	4.9
SO_2	3.44	3.47	0.5	0.01
**Total**			**554.1**	**9.7**

Source: Adapted from Nowak et al. (2006) and Hirabayashi (2014)

of the NCCGN. The market pricing method was used to determine the benefits of pollination on agricultural production. We measured the value of the pollination service based on the value of different cultures, their size and their respective rates of dependence on pollinators for fruit and vegetable production. We divided the net benefit (i.e. the balance of market benefits and production costs) of the crops within the one-kilometre buffer zone that were considered to be produced under the action of pollinators by the area under production. We did not consider the pollination service inside the NCCGN to avoid double counting with the market value of agricultural products. The value of pollination was then estimated at $31/ha/year for urban and rural forests in the NCCGN.

Forests are often used as an area to practice recreational activities. Gatineau Park was initially designed for recreational and cultural purposes, but it now has a stronger conservation mandate. The Park has a number of cycling, hiking, and nature observation trails, as well as cross-country skiing, snowshoeing, and winter bike paths. There are also 6 beaches, camping and canoe-camping sites, as well as yurts, cabins, and 4-season tents available for visitors. In 2014-2015, the NCC collected $2.7 million in user fees, which is equivalent to $75/ha over Gatineau Park's entire spatial area (NCC, 2014-2015 Annual Report: 49). Even if this amount gives only a partial representation of the total economic value of recreational services provided by the Park, we used that value as a proxy for the **recreation** service for the entire study site (as most of the activities available in the Park are free).

Water is essential to human life. Using the benefit transfer database, we measured a mean value of $340/ha/year for urban forests and $839/ha/year for rural forests in the water provisioning service.

Water is essential to human life. In 2013, Canada's capital region's daily mean consumption of water per person was 403 litres, and the main source of water is the Ottawa River. The Ottawa River watershed is very large, covering six administrative regions on the Quebec side, totalling 146,334 km^2 in Ontario and Quebec. Using the benefit transfer database, we measured a mean value of $340/ha/year for urban forests and $839/ha/year for rural forests in the **water provisioning** service.

Water quality is highly influenced by non-point source pollution coming from urban centres, agricultural lands, and industrial sites. A 2015 report from the *Ministère du Développement durable, de l'Environnement et de la Lutte contre les changements climatiques* (MDDELCC)

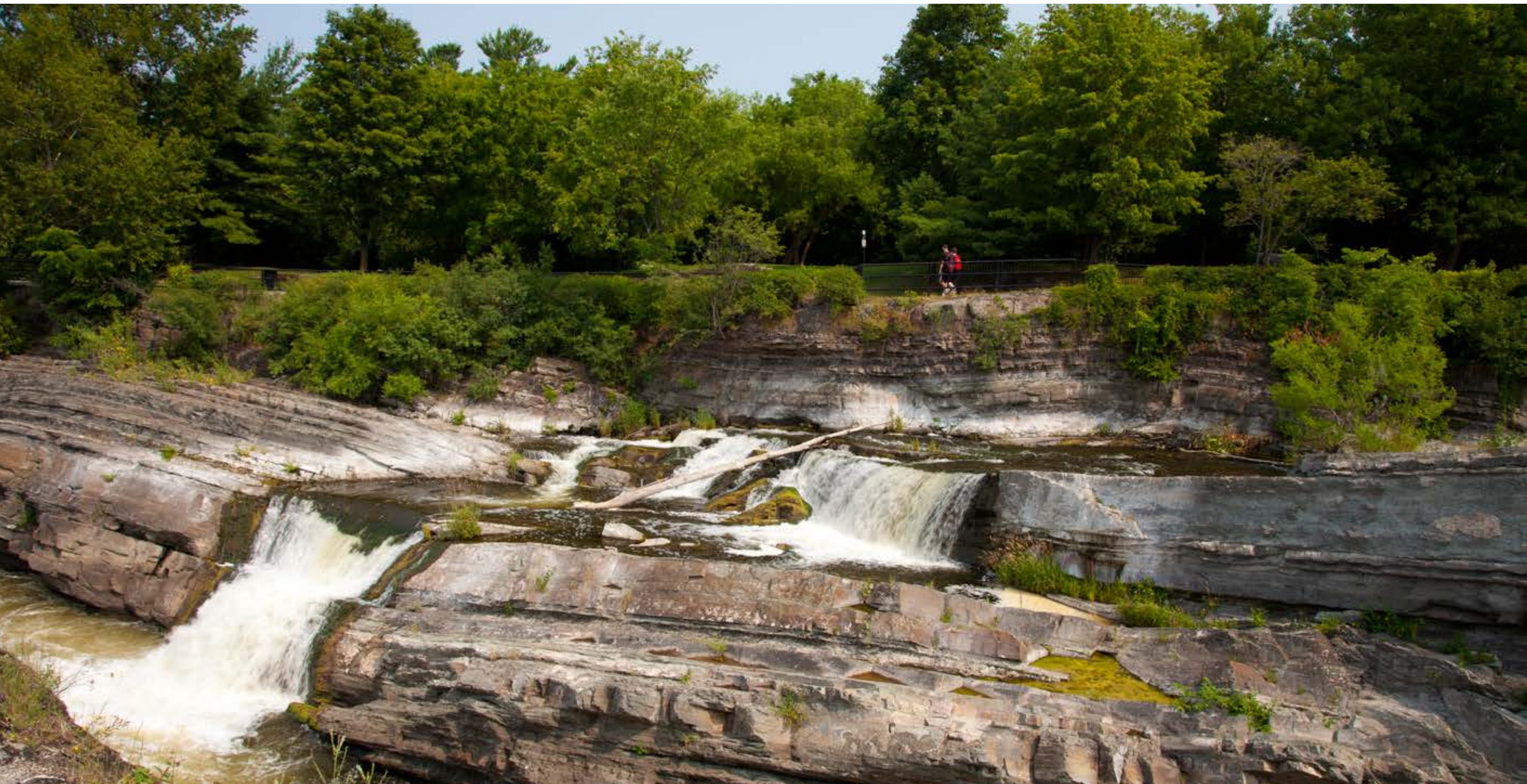

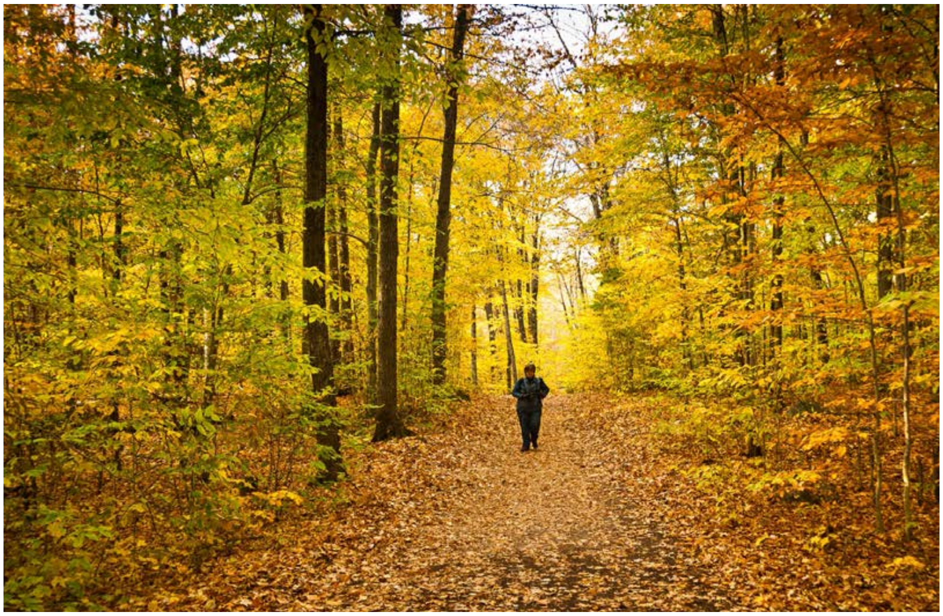

stated that water quality in the watershed was overall good, with some deficiencies in the area of Gatineau-Ottawa, where the land cover is characterized by a higher proportion of urban and agricultural lands. The presence of fewer trees and overall forest cover increases the proportion of nutrients and pollutants going directly into the river. Indeed, forests have the capacity to filter, store, and transform pollutants, so that they become less harmful. According to our data collection and transfer, the **waste treatment** service is estimated at $140/ha/year for urban forests and $318/ha/year for rural forests.

Trees and vegetated areas in urban settings ensure a better control of stormwater runoff through absorption, thus diminishing chances of runoff going directly into municipal sewers and natural waterways. Portions of sewer systems in Gatineau and Ottawa are combined. If rainfall is abundant, the sewer systems directly discharge wastewater in the Ottawa River. This situation imposes an environmental cost to the river, in addition to an economic cost due to the closure of beaches downstream following the discharges. The **disturbance prevention** service was estimated at $5,030/ha/year for the urban forests.

As presented in the study site description, the NCCGN is home for to many fauna and flora species. **Biodiversity habitat** is a supporting service that can directly or indirectly benefit human well-being. Based on 17 different monetary estimates, this service was valued at $2,688/ha/year for urban forests and $2,186/ha/year for rural forests.

Trees and forests control soil erosion through their leaves and their root system. The leaves of individual trees and forest canopy intercept rain and reduce the impact of rainfall, which reduces soil disturbance. On stream banks, root systems hold the soil in place, thus limiting the amount of soil going into streams after rainfall. Permeable soil can also control erosion by allowing water to seep into the ground, once again limiting soil disturbance. The value of **erosion control** is estimated at $211/ha/year for urban forests and at $137/ha/year for rural forests.

Forests and trees can provide pest management control by preventing plant diseases and diminishing the importance of insect pests because of the presence of natural enemies. For example, the U.S. Forest Service has determined that forest birds performed biological control in forests (Wilson 2008). The value of **pest management** was estimated at $42/ha/year for urban forests and at $28/ha/year for rural forests.

The natural process of leaf fall and, more generally, decay of plant and animal matter, provides organic matter to the forest. This organic matter is then decomposed and the minerals and nutrients are released into the forest soil to fuel plant growth. This process is called nutrient cycling. It represents $318/ha/year for rural forests.

The natural process of leaf fall and, more generally, decay of plant and animal matter, provides organic matter to the forest. This organic matter is then decomposed and the minerals and nutrients are released into the forest soil to fuel plant growth. This process is called **nutrient cycling**. It represents $318/ha/year for rural forests.

Table 5 shows the aggregated values for the urban and rural forests. For the 11 ecosystem services we measured, we found a yearly value per hectare of $9352 for urban forests and $4,183 for rural forests. The minimum total value for urban and rural forests is $11 and $19 million/year, respectively, and the maximum total value is $22 and $647 million per year.

TABLE 5. NON-MARKET VALUES PROVIDED BY THE FORESTS AND WOODLANDS OF THE NATIONAL CAPITAL COMMISSION'S GREEN NETWORK

Ecosystem Services	Nb. of $ estimates	Total area (ha)	Min. value	Max. value	Mean	St. deviation	Method	Total value ($k/y)
			($ per hectare per year)					
**Urban Forests**	**15**	**1552**	**6816**	**14,333**	**9352**			**14,514.3**
Global Climate Regulation	1		-	-	241	nd	RC	374.0
Air Quality	1		-	-	554	nd	BT	859.8
Water Provisioning	3		203	609	340	233	BT	527.7
Waste Treatment	1		-	-	140	nd	BT	217.3
Erosion Control	3		111	396	211	160	BT	327.5
Pollination	1		-	-	31	nd	MP	48.1
Biodiversity Habitat	3		444	7160	2688	3873	BT	4,171.8
Disturbance Prevention	2		4975	5085	5030	78	BT	7,806.6
Pest Management	1		-	-	42	nd	BT	65.2
Nutrient Cycling	-		-	-	-	-	-	-
Recreation	1		-	-	75	nd	MP	116.4
**Rural Forests**	**36**	**38,128**	**521,2**	**16,991**	**4183**			**159,489.4**
Global Climate Regulation	1		-	-	241	nd	RC	9188.9
Air Quality	1		-	-	10	nd	BT	381.3
Water Provisioning	5		123	3053	839	1252	BT	31,989.4
Waste Treatment	4		26	806	318	344	BT	12,124.7
Erosion Control	6		1	536	137	202	BT	5,223.5
Pollination	1		-	-	31	nd	MP	1,182.0
Biodiversity Habitat	14		0.1	11,349	2186	3673	BT	83,347.8
Disturbance Prevention	-		-	-	-	-	-	-
Pest Management	2		14	42	28	20	BT	1067.6
Nutrient Cycling	3		0.1	848	318	462	BT	12,124.7
Recreation	1		-	-	75	nd	MP	2859.6

BT: Benefit transfer; RC: Replacement cost; MP: Market pricing

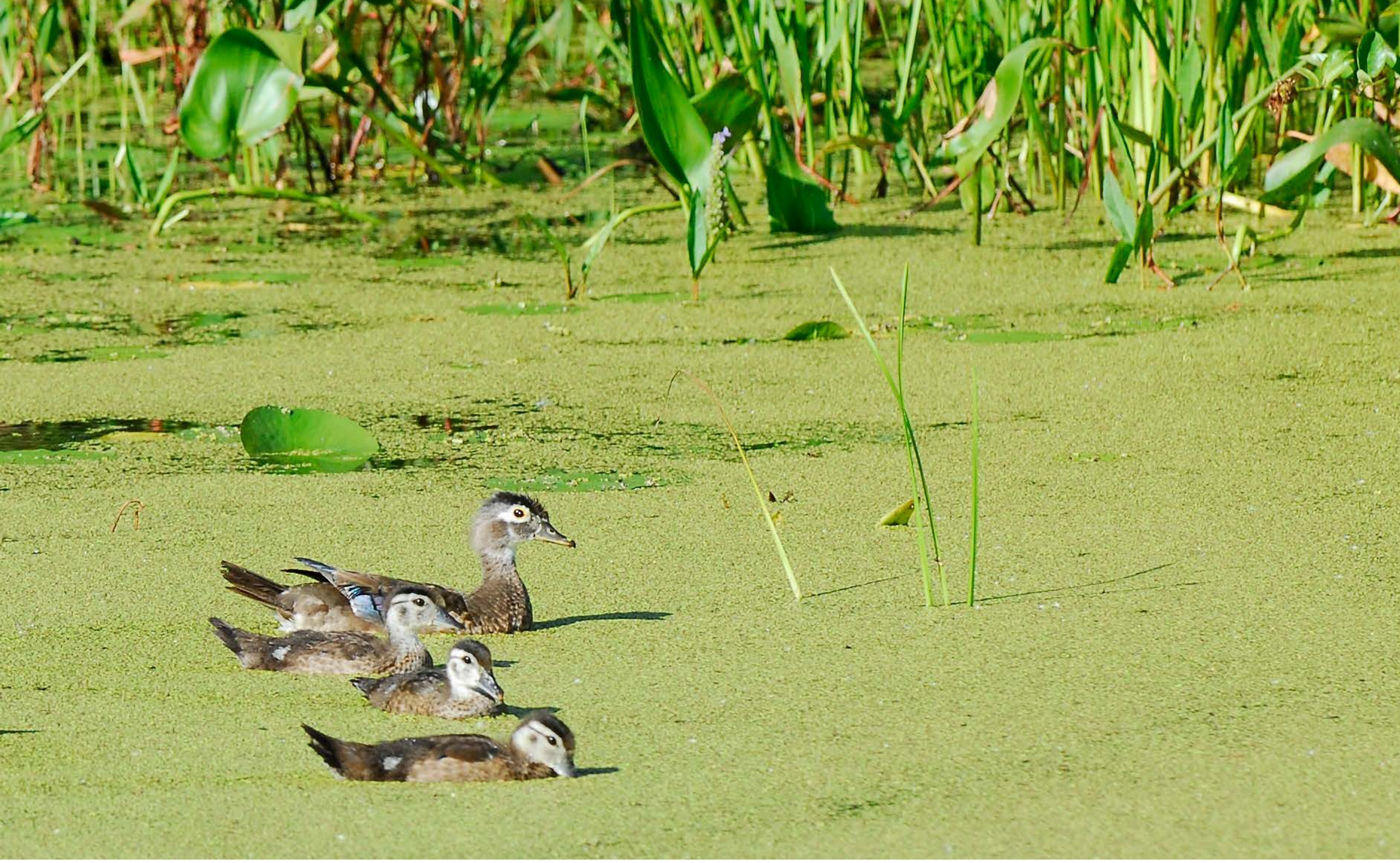

When considering the mean values for urban and rural forests, the total value for the forest ecosystem equals **$174 million per year**. The services with the highest values are habitat for biodiversity and disturbance prevention (for urban forests only). The mean value per hectare per year is highest for urban forests, because of significantly higher values per hectare for air quality and biodiversity habitat due to the importance of isolated urban forests in providing ecosystem services, and because there was no value found for disturbance prevention for rural forests.

In table 5, the standard deviation value is given when the Benefit Transfer (BT) method was used during the analysis, using at least two studies. This value is influenced by the number of estimates and their respective values. When there are few values and those are far apart, the standard deviation value can be very high.

Wetlands include lakes, marshes, swamps, bogs and fens. A quarter of the world's wetlands are located in Canada.

5.4 Wetlands

Wetlands are crucial to the Canadian landscape, as they provide a number of diverse ecosystem services. Wetlands include lakes, marshes, swamps, bogs, and fens. A quarter of the world's wetlands are located in Canada. However, wetlands are under threat by human activities like urban sprawl, drainage for agriculture, and commercial activities. In 2005, the Millennium Ecosystem Assessment reported that 80 to 98% of the wetlands located near major urban centres have disappeared worldwide. In Canada, Ducks Unlimited estimates that 70% of wetlands have disappeared or have been degraded in the country's inhabited areas. Ontario's

FIGURE 5. WETLANDS OF THE NATIONAL CAPITAL COMMISSION'S GREEN NETWORK

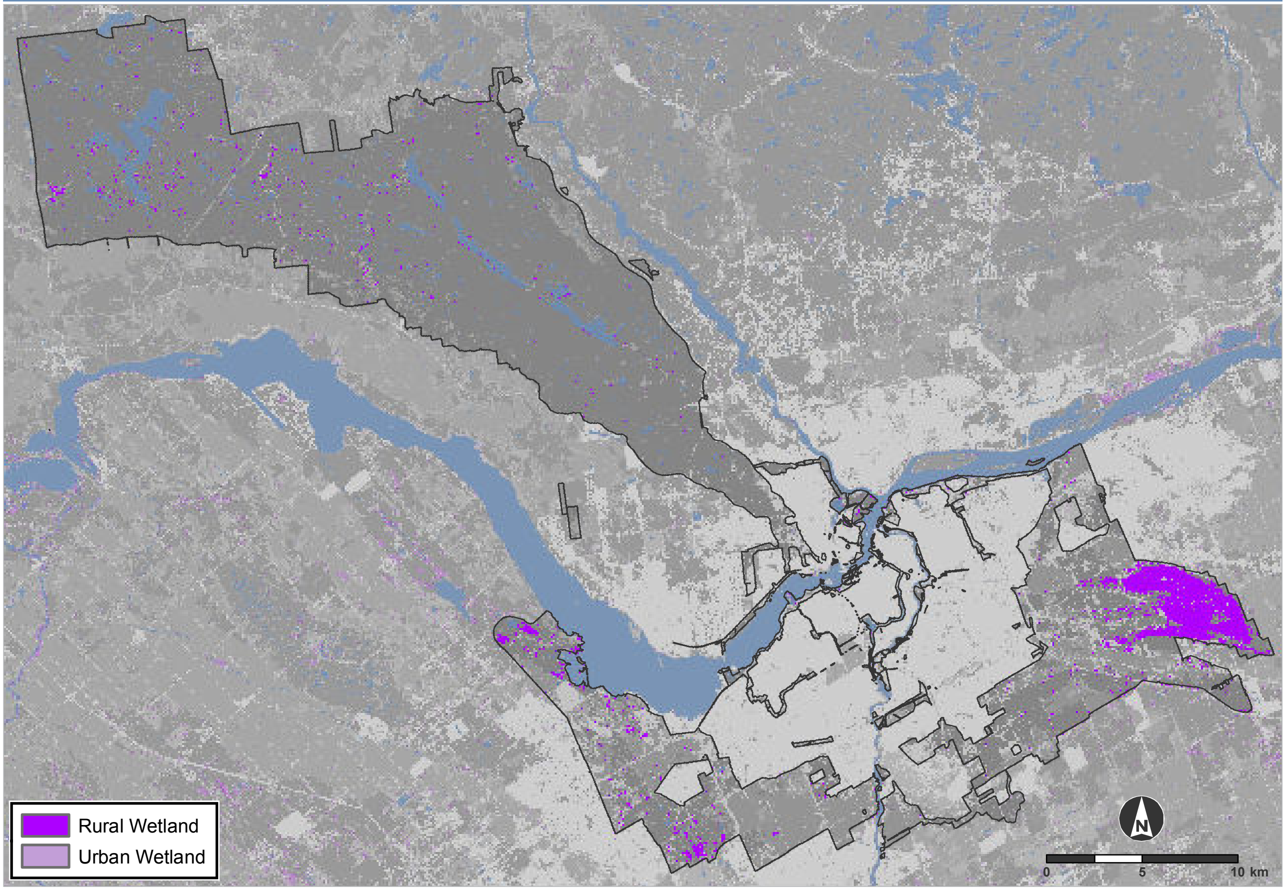

Figure 5 shows the wetland distribution in the NCCGN. We note the importance of Mer Bleue, the wetland complex located east of the region and representing most NCCGN wetlands.

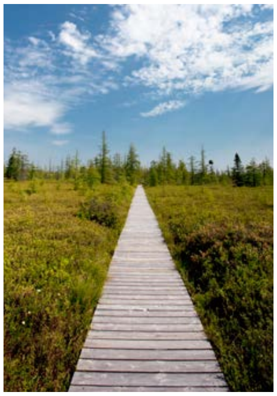

Ministry of Natural Resources has identified 14,000 ha of significant wetlands in the Greater Ottawa area; six of these are key complexes: Mer Bleue, Leitrim, Stony Swamp, Shirley's Bay, Mud Lake and Petrie Island (City of Ottawa). On the Quebec side of the NCR, 8,104 ha of wetlands were identified by Ducks Unlimited in 2007 in the Collines-de-l'Outaouais region and in the city of Gatineau. Work is still underway to locate every wetland in the region (Ducks Unlimited, http://maps.ducks.ca/cwi/).

Wetlands represent 4.5% of the NCCGN landscape. Two protected wetlands are in the Greenbelt: Mer Bleue and Stony Swamp. Mer Bleue is a 7,700-year-old bog. This 3,500 hectare conservation area has a northern ecosystem more typical of the Arctic than the Ottawa Valley. It is an important wetland for climate regulation and research activities, and represents a good habitat for fauna and flora. Stony Swamp is composed of wetlands, forests, and beaver dams; it is also crucial to biodiversity and scientific research. It also offers a great variety of recreational activities. Gatineau Park also hosts an extensive network of bogs and wetlands. In addition to the ecosystem services previously mentioned, these land cover types provide carbon sequestration and storage, water filtration and provisioning, waste treatment, groundwater recharge, biodiversity habitat, and recreation. Figure 5 shows the wetland distribution in the NCCGN. We note the importance of Mer Bleue, the wetland complex located east of the region and representing most NCCGN wetlands.

For the economic evaluation of wetlands, we used a meta-analysis approach based on the spatial analysis method. We analysed land cover composition for each 50km^2 sub-region.

Thus, the value of each wetland is representative of its environment in terms of urbanized and agricultural land within a ten-kilometre radius.

To evaluate the economic value of four of the ES provided by these wetlands, the meta-analysis benefit transfer methodology integrated different criteria, including wetland size and type, GDP per capita, and land cover composition around the wetlands. After performing the spatial analysis for each wetland, we found a value of $21,461/ha/year for the **biodiversity habitat** service, $15,893/ha/year for the **waste treatment** service and $20,766/ha/year for the **disturbance prevention** service. No value was found for market services, since there are no commercial activities in the NCCGN wetlands.

The high value of these services is due to several economic and environmental factors. First, the statistical model we used links the economic value of wetlands to their abundance and ecological importance in the landscape. In this sense, the more wetlands are rare and surrounded by urban and agricultural areas in a given environment, the more essential their ecological roles in terms of ES will be. Second, in socio-economic terms, the meta-analysis model states that the value of services provided by wetlands varies depending on the relative wealth of the population. For example, the service of flood protection will have a higher value in a territory where infrastructure and houses have a higher value and where the size of the population is larger. Since GDP per person in the NCR is relatively high, the value of wetlands is thereby increased. In summary, the scarcity of wetlands, their geographic location, demographics, and the regional economy explain the high value of these services.

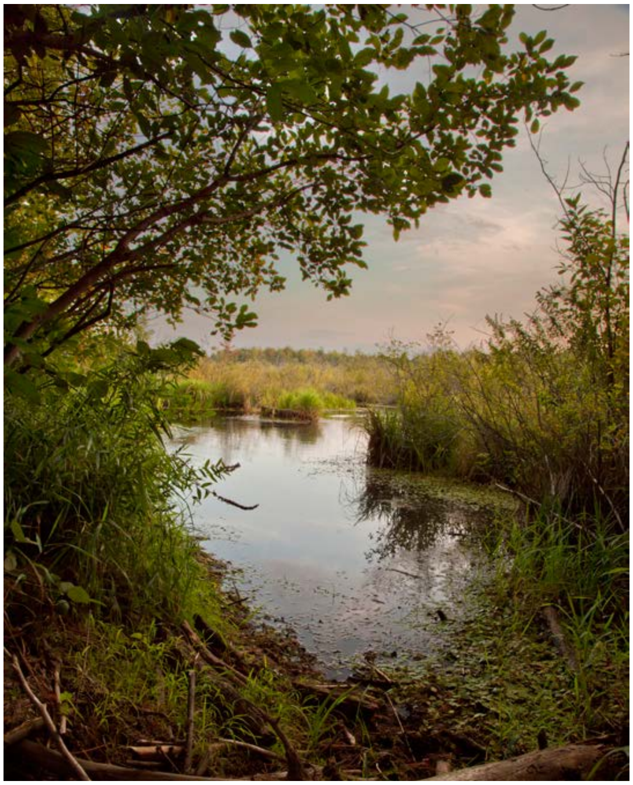

Services related to habitat for biodiversity, prevention of extreme events and water purification (i.e. waste treatment) have by far the highest economic value (more than $15,000/ha/year).

Wetlands are important carbon sinks. In a recent work, Garneau and Van Bellen (2016) estimated the carbon stocks in peat bogs across Quebec. We used these values to estimate the carbon stocked in the NCCGN wetlands. By using the values of three representative regions of the NCCGN wetland systems (i.e. Ottawa Plain, Mont-Laurier Depression, Mt-Tremblant Range), we measured an average of 1468 tonnes of C/ha. Using the value of the SCC ($43/tonne of CO_2), a 50-year annualization, and a 3% discount rate, we come up with a carbon stock value of $1,057/ha/year.

The estimation of carbon sequestration is based on the rate found in Mer Bleue wetlands—0.7 tC/ha/year (Lafleur et al. 2001), for an economic value of $111/ha/year. By combining these two values, we find a value of $1,168/ha/year for the **climate regulation** service.

Using the benefit transfer database, a value of $31/ha/year was also determined for **water provisioning**.

For the **recreation** service, we used the value determined by the Gatineau Park analysis (see section 5.3), which is $75/ha/year.

Table 6 provides a summary of measured values for wetlands and figure 6 shows the distribution of the wetlands' value. Isolated wetlands have higher values as their role in terms of ES is more important in sectors where they are scarce.

Services related to habitat for biodiversity, prevention of extreme events, and water purification (i.e. waste treatment) have by far the highest economic value (more than $15,000/ha/year). All six assessed services have a combined value of $59,394/ha/year, totalling $146 million/year. Considering the meta-analysis method we used, there is very little

variance in the values we measured. These significant values illustrate their big contribution to the quality of life and safety of regional communities. As reviewed in The Economics of Ecosystems and Biodiversity (TEEB 2013), maintaining and restoring wetlands often lead to cost savings when compared to built-up solutions.

TABLE 6. NON-MARKET VALUES PROVIDED BY THE NATIONAL CAPITAL COMMISSION'S GREEN NETWORK WETLANDS

Ecosystem Services	Nb. of $ estimates	Total area (ha)	Min. value	Max. value	Mean	St. deviation	Method	Total value ($k/y)
			($ per hectare per year)					
Urban and Rural Wetlands	7	2453	59,371	59,417	59,394			145,693.5
Global Climate Regulation	1		-	-	1168	nd	RC	2865.1
Water Provisioning	2		8	54	31	33	BT	76.0
Waste Treatment	1		-	-	15,893	nd	BT	38,985.5
Biodiversity Habitat	1		-	-	21,461	nd	BT	52,643.8
Disturbance Prevention	1		-	-	20,766	nd	BT	50,939.0
Recreation	1		-	-	75	nd	MP	184.0

BT: Benefit transfer; RC: Replacement cost; MP: Market pricing

FIGURE 6. ECONOMIC VALUE OF THE WETLANDS OF THE NATIONAL CAPITAL COMMISSION'S GREEN NETWORK (VALUES EXPRESSED IN $/HA/YEAR)

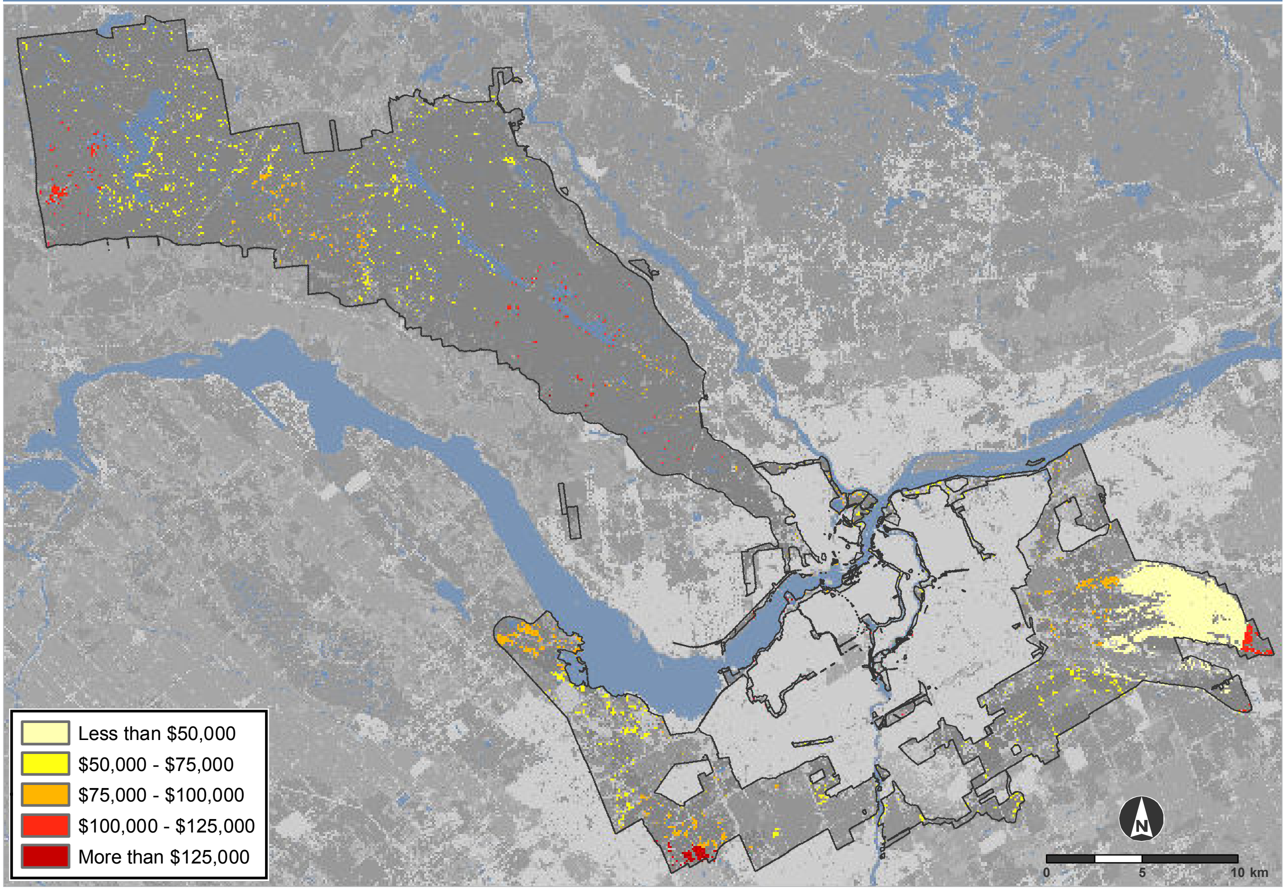

5.5 Croplands

Agriculture is a main component of the region's economy as it provides jobs, generates economic benefits, and provides local produce. Agricultural lands represent 10% of the land cover managed by the NCC. The main agricultural crops grown in the Greenbelt are soy, corn, and barley. Fruits, vegetables and herbs are also produced; visitors can buy these products directly on the farm or at various farmers' markets across town.

Several equine farms are present in the Greenbelt, which offer interesting recreational activities. Figure 7 presents the location and distribution of all the agricultural productions in the study region.

There is a wide variety in agricultural production in the Greenbelt. To evaluate the net benefit of each **agricultural production** ($/ha/year), we used the market pricing method. This calculation is done by measuring the difference between the gross income (value of agricultural product sales on markets) and production costs. These costs include costs of labour, machinery, and various inputs for agricultural production. The net benefit was calculated for barley, oat, wheat, corn, soy, dry beans, berries (i.e. strawberries), and other cereals. Their values vary from $304 to $16,925 per ha per year. We took the data from the *Financière agricole du Québec* (FADQ), the CRAAQ, and the Ontario Ministry of Agriculture, Food and Rural Affairs (OMAFRA). The total value of the net benefit of agricultural production was evaluated at a mean of $919/ha/year ($3 million per year). The value per crop is given in the Table 7.

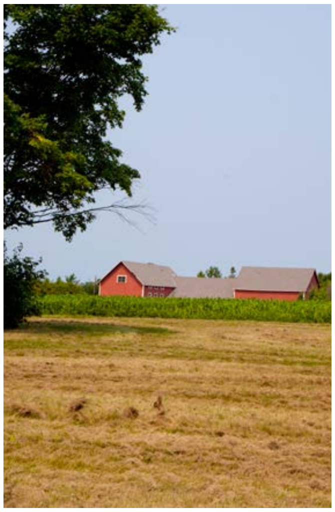

Agriculture is a main component of the region's economy as it provides jobs, generates economic benefits, and provides local produce.

FIGURE 7. AGRICULTURAL LAND USE IN NATIONAL CAPITAL COMMISSION'S GREEN NETWORK

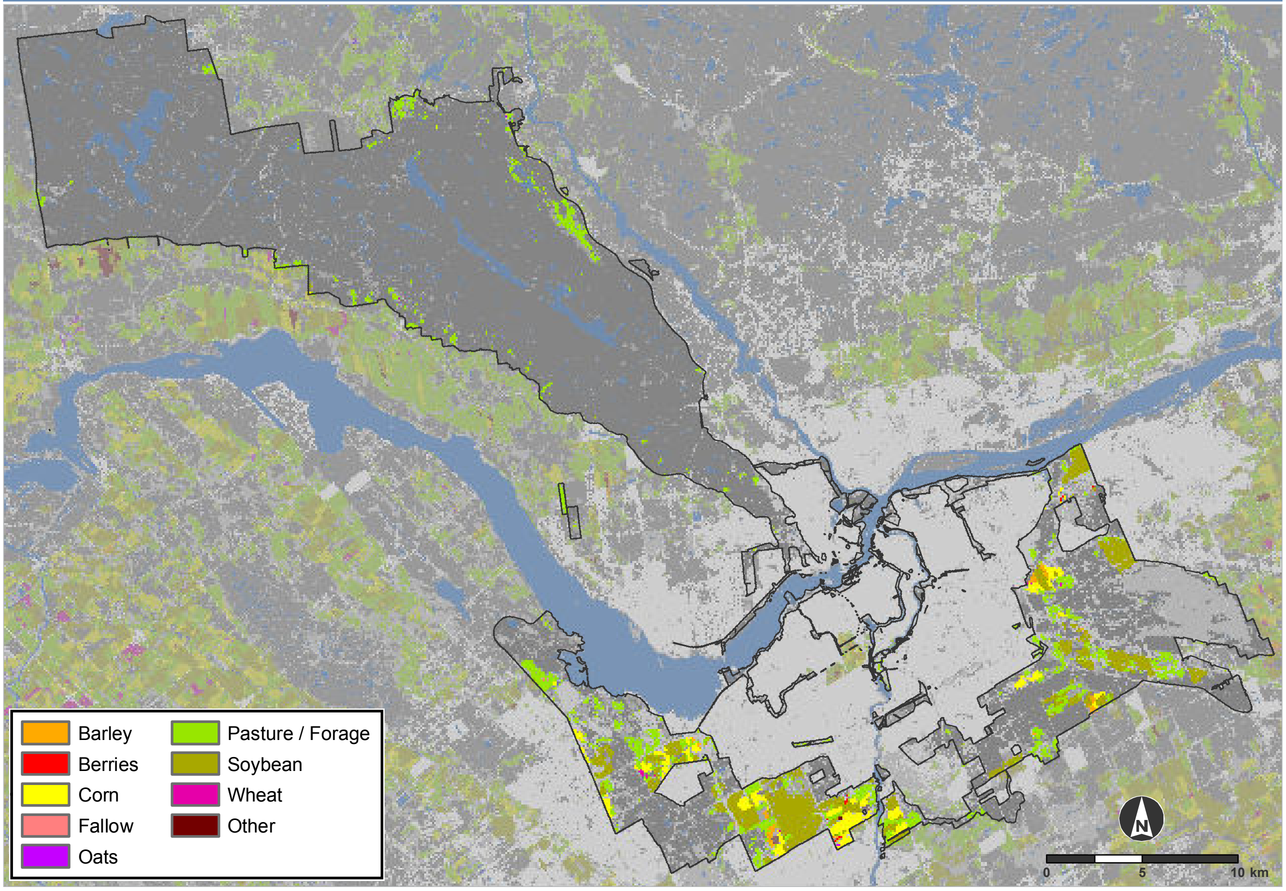

TABLE 7. MARKET VALUES PROVIDED BY AGRICULTURE LANDS OF THE STUDY AREA

	Benefits	Production Costs	Net benefit	Area (ha)	Total value (k$/y)
	($ per hectare per year)				
**Crops**				3306	3037.0
Barley	1462	962	500	104	52.0
Beans	1479	1175	304	3	1.0
Berries	29,837	12,912	16,925	17	287.7
Corn	3057	1913	1144	769	879.7
Fallow	-	-	-	1	-
Oats	1413	902	511	3	1.5
Soybeans	1859	1104	755	2362	1783.3
Wheat	1885	1206	679	20	13.6
Other Grains	-	-	674	27	18.2

Sources: FADQ, 2013; CRAAQ, 2011, 2014; OMAFRA, 2014; NCC.

Crops that remain on the land yearly, such as orchards, have a higher capacity to capture and store carbon, since there is less disturbance and surface decomposition.

Complementary to agriculture, agro-tourism is a recreo-tourist activity that takes place on a farm. It puts agricultural producers in contact with tourists, allowing them to discover the agricultural environment, agriculture, and its production through contact with farmers. Agro-tourism encourages closer relations between citizens and the agricultural world. Since no specific data for agro-tourism are available for the study site, we used a value from Dupras and Alam (2015) who estimated the **recreation service** at $88/ha/year (based on the income from the agro-tourism of 66 agro-businesses in the Greater Montreal area).

To measure the value of other non-market ES, we used the database and identified values for **erosion control** ($106/ha/year), **nutrient cycling** ($174/ha/year), and **landscape aesthetics** ($76/ha/year). Although agricultural areas may provide many other types of ES, the high variability in crops and farming practices prevents us from identifying values that are uniform and transferable to all kinds of crops. For example, in **climate regulation,** agricultural lands have the potential to store carbon in soils and they have the capacity to absorb carbon through plants. Crops that remain on the land yearly, such as orchards, have a higher capacity to capture and store carbon, since there is less disturbance and surface decomposition (Wilson 2008). Emissions from the agricultural sector accounted for 10% of total emissions among greenhouse gases in Canada (AAFC 2015). Since agricultural activities can act as both sources and sinks for CO_2, N_2O and CH_4, we have decided not to calculate a value for the agricultural sector.

Table 8 summarizes the values associated with agricultural lands. In terms of provisioning services, the food production value amounted to $3.04 million/year. For regulating services, both identified ES (erosion control and nutrient cycled) contributed of $0.92 million/year. Agricultural lands also offer intangible benefits in the form of cultural services. These services include aesthetic value and recreational value and have a combined value of $0.54 million/year. The minimum total value identified equals $4 million/year, whereas the maximum total value equals $5 million/year. Finally, when we consider the mean value, the total ES value produced by agricultural areas reaches **$4.51 million per year**, an average of $1,363/ha/year.

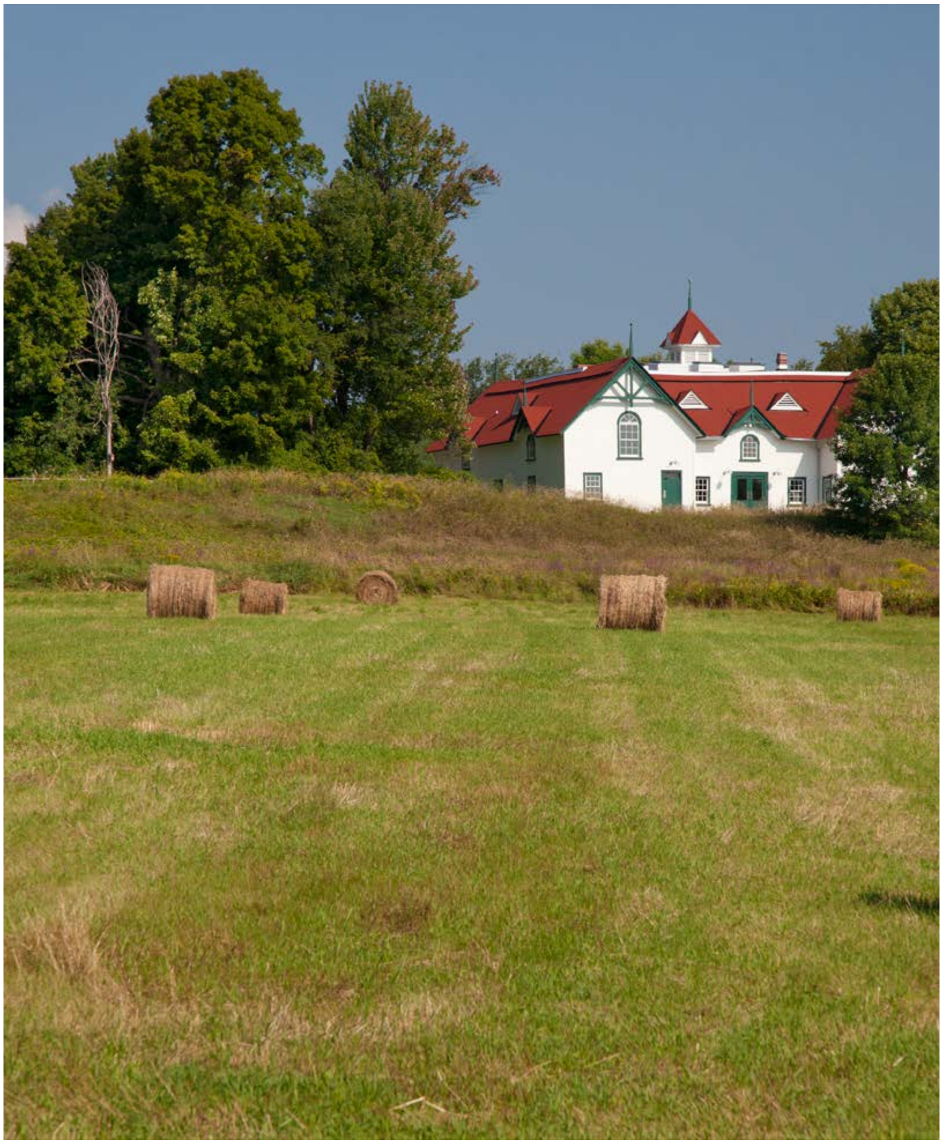

Although agricultural areas may provide many other types of ES, the high variability in crops and farming practices prevents us from identifying values that are uniform and transferable to all kinds of crops.

TABLE 8. MARKET AND NON-MARKET VALUES PROVIDED BY CROPLANDS IN THE NATIONAL CAPITAL COMMISSION'S GREEN NETWORK

Ecosystem Services	Nb. of $ estimates	Total area (ha)	Min. value	Max. value	Mean	St. deviation	Method	Total value ($K/y)
			($ per hectare per year)					
Croplands (Annual Crops)	12	3306	1192	1494	1363			4506.1
Food Production	1		-	-	919	nd	MP	3038.2
Erosion Control	1		-	-	106	nd	BT	350.4
Nutrient Cycling	3		58	190	174	68	BT	575.2
Aesthetics	6		21	191	76	64	BT	251.3
Recreation	1		-	-	88	nd	BT	291.0

BT: Benefit transfer; MP: Market pricing

5.6 Prairies, Pastures and Grasslands

Prairies, pastures, and grasslands provide valuable services and habitat for a large number of species (Tilman et al. 2001; MEA 2005). Croplands meet immediate material needs, but by generating a diversified habitat mosaic, grasslands are important for non-material human well-being. They provide a wide range of ES such as pest management, pollination, nutrient cycling, and direct and indirect crop production (Tilman et al. 2001). In that sense, the beneficial effects of non-intensive agricultural land use, such as grasslands and open fields, are still important for biodiversity habitat conservation and its management.

To assess the market value of **agricultural products** from grasslands and forage crops, we proceeded in the same way as for croplands. Thus, we calculated an economic rent, the balance between income coming from the sale of hay and the costs of its production. The resulting value is $116/ha/year (Table 9).

In terms of erosion control, grasslands, prairies, and pastures maintain ground cover and help reduce erosion caused by wind and water runoffs.

TABLE 9. MARKET VALUES PROVIDED BY FORAGE LANDS IN THE NATIONAL CAPITAL COMMISSION'S GREEN NETWORK

	Benefits	Production Costs	Net benefit	Area (ha)	Total value (k$/y)
	($ per hectare per year)				
Forage	629	513	116	2320	269,1

In terms of **climate regulation**, Smith et al. (2001) estimated that grasslands, pasture, and forage crops contain 105 tonnes of carbon per hectare on average. The annualized value of this stock over 50 years, with a 3% discount rate and a value of $43/ton, is $76/ha/year. For the annual sequestration, Klumpp et al. (2011) measured an annual sequestration rate of 2.17 tonnes of C/ha, equivalent to an annual value of $342/ha/year. The combined value of carbon storage and sequestration is equivalent to an annual value of $418/ha.

For **recreation** and **pollination services**, we used the methodology based on market prices detailed in section 5.3. We've obtained values of $75/ha/year and $31/ha/year.

In terms of **erosion control**, grasslands, prairies, and pastures maintain the ground cover and help reduce the erosion caused by wind and water runoff. Using the benefit transfer database, we found three studies that economically valued these land use covers at a mean of $109/ha/year. The same database was used to estimate values for four additional services: biodiversity habitat, pest management, nutrient cycling, and landscape aesthetics.

Biodiversity habitat is part of the Habitat or Supporting services category. It shows the highest value for ES in this land cover type, i.e. $2,324 ha/year. Prairies, pastures, and especially grasslands are recognized as species-rich ecosystems for vegetation, which has value in itself, but is also important to support a diversity of species of birds, reptiles, amphibians, and mammals, including a number of species at risk (Tilman et al. 2001).

The ability of prairies, pastures, and grasslands to provide **pest management** service can be derived from their high level of biodiversity, which influences biological control provided by natural enemies. This service has an estimated economic value of $42/ha/year.

Nutrient cycling is a service provided by a number of ecosystems, including prairies, pastures, and grasslands. It involves decomposing organic matter, incorporating minerals and nutrients into the soil, and forming an organic layer. These minerals and nutrients are

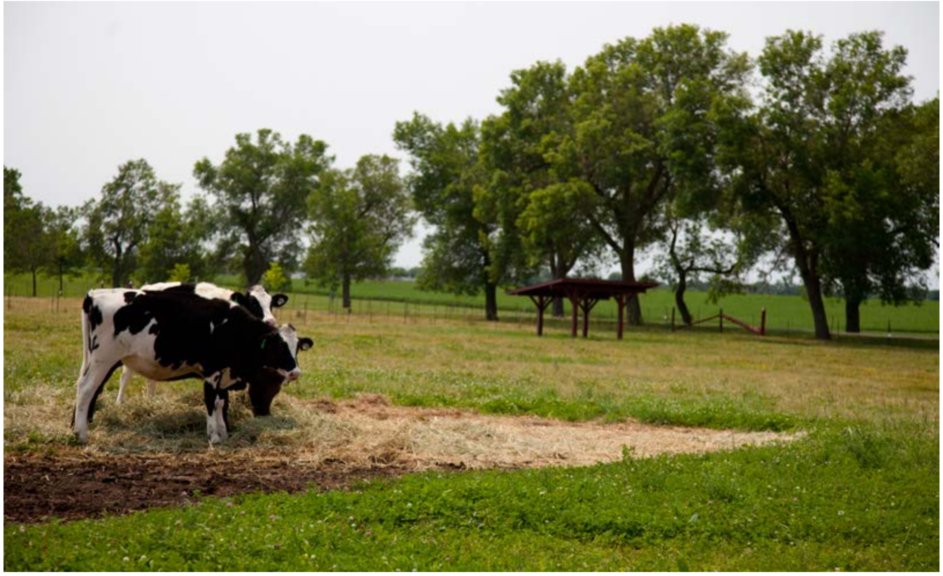

then taken up by plants that produce oxygen and absorb carbon dioxide. The cycle resumes when the plants are either eaten by animals or are left to decay. Based on our benefit transfer analysis, this service has a value of $147/ha/year.

The beauty of a landscape is an essential cultural value, and its associated economic value varies based on the type of landscape and the people who value it. As a result, the economic value of landscape aesthetics is often estimated using surveys. In this case, we've used the benefit transfer approach and obtained a mean value of $76/ha/year for the **landscape aesthetics** service.

As presented in Table 10, the yearly value per hectare for prairies, pastures, and grasslands is $3,338 with little variance, for a total value of $8 million/year.

The beauty of a landscape is an essential cultural value, and its associated economic value varies based on the type of landscape and the people who value it.

TABLE 10. MARKET AND NON-MARKET VALUES PROVIDED BY PASTURES, PRAIRIES, AND GRASSLANDS IN THE NATIONAL CAPITAL COMMISSION'S GREEN NETWORK

Ecosystem Services	Nb. of $ estimates	Total area (ha)	Min. value	Max. value	Mean	St. deviation	Method	Total value ($k/y)
			($ per hectare per year)					
Pastures and Grasslands	14	2320	3235	3537	3338			7744.2
Agricultural Products	1		-	-	116	nd	MP	269.1
Global Climate Regulation	-		-	-	418	-	RC	969.8
Erosion Control	3		61	193	109	73	BT	252.9
Pollination	1		-	-	31	nd	MP	71.9
Biodiversity Habitat	1		-	-	2324	nd	BT	5391.7
Pest Management	1		-	-	42	nd	BT	97.4
Nutrient Cycling	1		-	-	147	nd	BT	341.0
Aesthetics	6		21	191	76	64	BT	176.3
Recreation	1		-	-	75	nd	MP	174.0

BT: Benefit transfer; RC: Replacement cost; MP: Market pricing

5.7 Freshwater Systems

In the context of this study, the aquatic systems refer to lakes, streams, and two rivers: the Rideau River and the La Pêche River. Both cross through the Greenbelt and Gatineau Park. All of them are located in the Ottawa River watershed, but are part of different sub watersheds. They represent 5% of the total land area under study (Figure 8).

Freshwater environments are a vital habitat for biodiversity. They allow for food and water provisioning, recreational activities, and waste treatment.

There are few studies on the economic value of freshwater systems, even though biophysical relationships between ecosystem services and these systems have been valued extensively (Mueller et al. 2016). Most of the studies we found evaluate ES using hedonic pricing, and thus focus on amenity value of waterfront private properties. These values are hardly useful in the current context, as the NCC owns most of the properties and is a federal Crown Corporation. Additionally, the cultural value of freshwater systems has already been included in the recreational value for forests. We consequently used the value found for Gatineau Park ($75/ha/year) to value the **recreation** service associated with freshwater systems.

To find values for other relevant ecosystem services, we used the benefit transfer approach to transfer values measured for the Blue Network of the Greater Montreal area (Poder et al. 2015). In this study, the authors used a stated preference method (i.e., contingent ranking)

Freshwater environments are a vital habitat for biodiversity. They allow for food and water provisioning, recreational activities, and waste treatment.

FIGURE 8. FRESHWATER SYSTEMS IN THE NATIONAL CAPITAL COMMISSION'S GREEN NETWORK

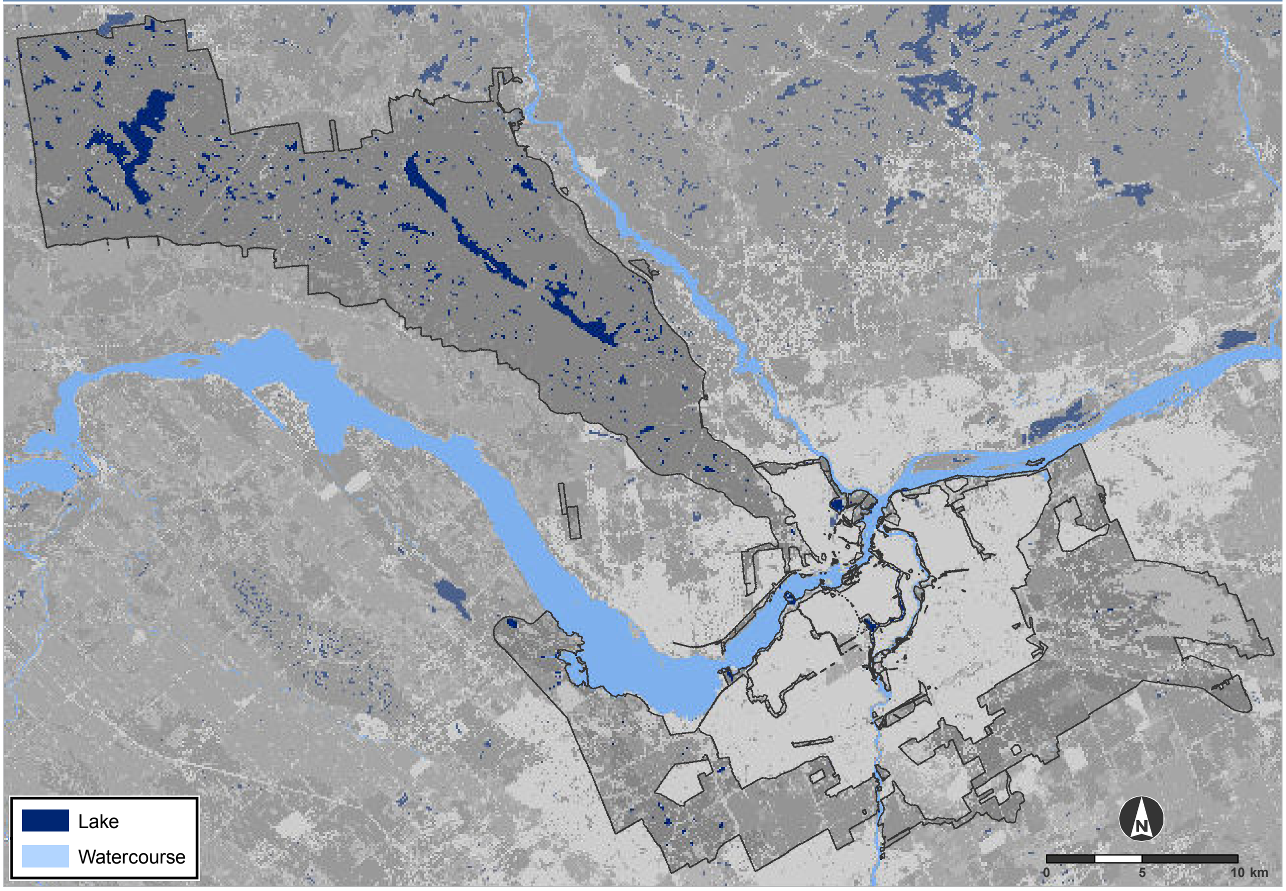

to measure the willingness to pay of the Quebec population for improving the quality of the water systems in the Montreal Greenbelt. Based on three ongoing restoration projects in riparian zones and water systems on Montreal Island and the south and north shores, the authors found economic values for improving the biodiversity habitat, waste treatment, and aesthetics.

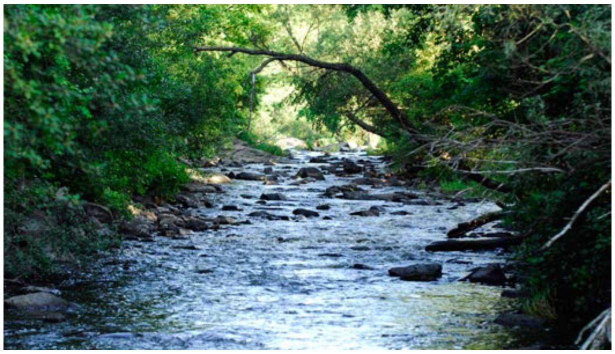

To value the NCCGN freshwater systems, we consider that these indicators represent the willingness to pay of citizens to avoid deteriorating their aquatic environment. To identify values coherent with the region's population, we multiply the willingness to pay per household for the selected services by the number of households in the region and divide it by the total size of the freshwater system in the Ottawa/Gatineau Metropolitan region (see Table 11 for details). The total value obtained was then annualized over 20 years at an actualization rate of 3%. We then obtain a value of $10/ha/year for **biodiversity habitat**, $48/ha/year for **waste treatment**, and $4/ha/year for **aesthetics**. Freshwater systems offer habitat to fish species and plants, and provide food for a number of other species. They are indispensable critical tool for biodiversity. These systems also provide waste treatment services by diluting or degrading pollutants through aquatic plants. The aesthetic value is related to the beauty and aesthetics appreciation of the freshwater systems.

Freshwater systems offer habitat to fish species and plants, and provide food for a number of other species. They are indispensable critical tool for biodiversity.

TABLE 11. VALUES FOR THREE ECOSYSTEM SERVICES DERIVED FROM THE MONTREAL BLUEBELT ECOSYSTEMS

Ecosystem Services	WTP for the Montreal Blue Network ($/hh)	Nb of Households in the Ottawa/ Gatineau Region	Freshwater Systems area in the Ottawa/ Gatineau Region (ha)	Value for the CCRGN services ($/ha/y)
Biodiversity Habitat	25	340,515	24,897	10
Waste Treatment	218	340,515	24,897	48
Aesthetics	10	340,515	24,897	4

WTP: Willingness to pay; hh: Household

As presented in Table 12, the aggregated value for freshwater system is $137/ha/year, for a total yearly value of $0.225 million per year.

TABLE 12. MARKET AND NON-MARKET VALUES PROVIDED BY FRESHWATER SYSTEMS IN THE NATIONAL CAPITAL COMMISSION'S GREEN NETWORK

Ecosystem Services	Nb. of $ estimates	Total area (ha)	Min. value	Max. value	Mean	St. deviation	Method	Total value ($k/y)
			($ per hectare per year)					
Aquatic Systems	5	1643	137	137	137			225.1
Biodiversity Habitat	1		-	-	10	nd	BT	16.4
Waste Treatment	1		-	-	48	nd	BT	78.9
Aesthetics	1		-	-	4	nd	BT	6.6
Recreation	1		-	-	75	nd	MP	123.2

BT: Benefit transfer; MP: Market pricing

5.8 Total Economic Value

Table 13 presents a summary of the estimated ES values for each ecosystem studied (dollars per hectare per year). Wetlands generate the highest value with $59,394/ha/year for six ES. Urban forests, rural forests, and prairies and grasslands follow, with ES values of $9,352/ha/year, $4,183/ha/year, and $3,338/ha/year, respectively. Finally, croplands and freshwater systems each provide $1,363/ha/year and $137/ha/year.

Table 14 provides the total values for each ecosystem service per ecosystem type. The service presenting the highest value is biodiversity habitat ($145 million per year), followed by disturbance prevention ($59 million per year) and waste treatment ($51 million per year).

Despite the fact that wetlands have a higher value for ecosystem services per hectare, the highest value is generated by rural forests with $159 million per year. Wetlands follow with $146 million per year, urban forests at $15 million per year, prairies and grasslands at $8 million per year, croplands at $4.5 million per year, and freshwater systems at $0.2 million per year.

The total economic value for all ecosystem services is **$332 million per year.** When considering the minimum and maximum values for each ecosystem (Table 15), this value ranges from $188 to $829 million per year.

The values in the previous tables are expressed in annual terms. If we consider the yearly fluxes of ES produced by the NCCGN ecosystems as a product of natural capital, we can convert yearly values to a total value. To do so, we consider the value of the NCCGN as being the sum of 20 years of ES, actualized at a rate of 3%, totalling **$5.037 billion.** The distribution of ES values across the NCCGN is shown in Figure 9.

The total economic value for all ecosystem services is $332 million per year. When considering the minimum and maximum values for each ecosystem (Table 15), this value ranges from $188 to $829 million per year.

TABLE 13. VALUES PER HECTARE FOR THE STUDIED ECOSYSTEM SERVICES

Ecosystem Services	Urban Forests	Rural Forests	Wetlands	Croplands	Prairies, grasslands	Freshwater Systems
	$ per hectare per year					
Agricultural Products	-		-	919	116	-
Global Climate Regulation	241	241	1168	-	418	-
Air Quality	554	10	-	-	-	-
Water Provisioning	340	839	31	-	-	-
Waste Treatment	140	318	15,893	-	-	48
Erosion Control	211	137	-	106	109	-
Pollination	31	31	-	-	31	-
Biodiversity Habitat	2688	2186	21,461	-	2324	10
Disturbance Prevention	5030	-	20,766	-	-	-
Pest Management	42	28	-	-	42	-
Nutrient Cycling	-	318	-	174	147	-
Aesthetics	-	75	-	76	76	4
Recreation	75	75	75	88	75	75
TOTAL	9352	4183	59,394	1363	3338	137

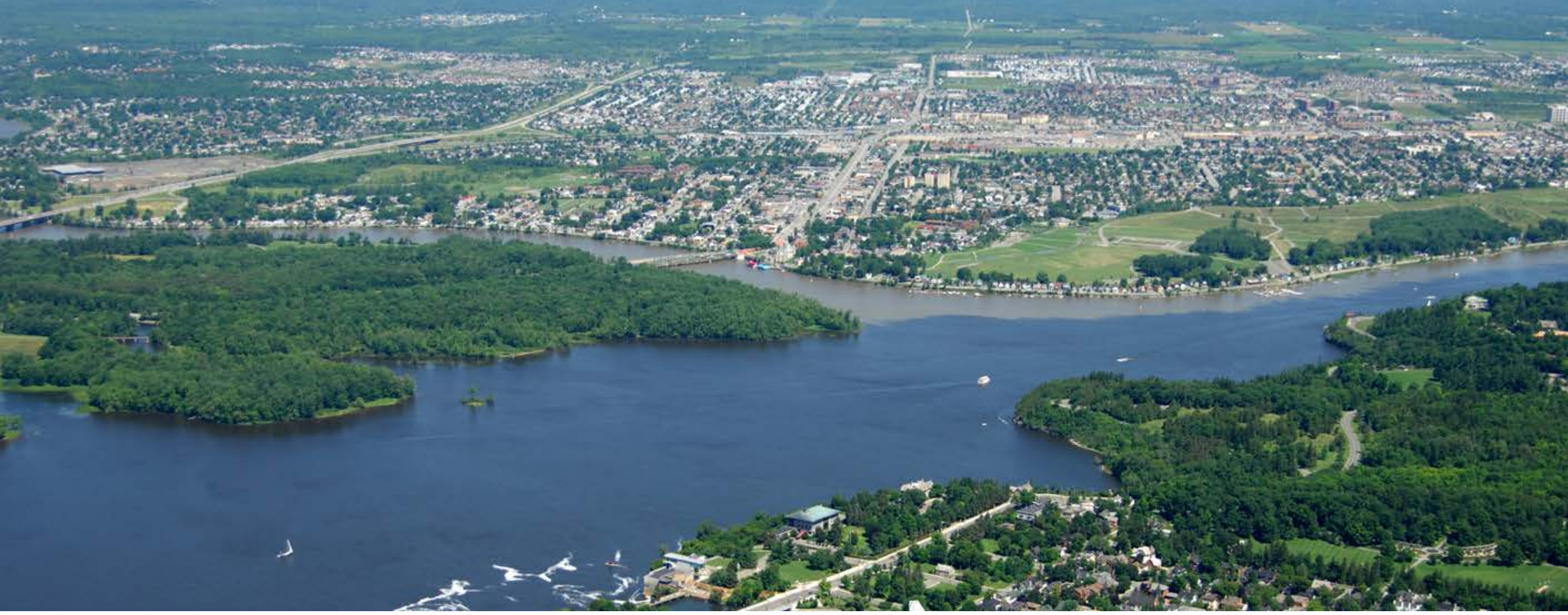

The monetary estimation we provide must be considered in light of methodological limitations inherent to the approach used in this study. The uncertainties that must be considered are related both to the benefit transfer methodology and the available data. First, the benefit transfer implies that values transferred from a starting location to a target site meet all the ecological and socio-economic conditions. Although care has been taken to minimize transfer bias, this approach should not be considered an exact science. Secondly, research efforts and studies published in the scientific literature are relatively unequal for different ecosystems. For example, data on forests and wetlands are more abundant than those of other ecosystems we studied, such as agricultural land and freshwater systems. Consequently, the estimates provided for each ecosystem do not have the same accuracy.

Values determined are an estimate of the ecosystem value for some services and as such, they must be considered as an order of magnitude more than a precise measurement. The results of this study must then be put in perspective and be seen as a first estimate of the value of the NCCGN's natural capital.

PHOTO COURTESY TOM ALFÖLDI

TABLE 14. TOTAL VALUES FOR THE STUDIED ECOSYSTEMS

Ecosystem Services	Urban Forests	Rural Forests	Wetlands	Croplands	Prairies, grasslands	Freshwater Systems	Total
	k$/year						
Agricultural Products	-		-	3038.2	269.1	-	3307.3
Global Climate Regulation	374.0	9188.9	2865.1	-	969.8	-	13,397.7
Air Quality	859.8	381.3	-	-	-	-	1241.1
Water Provisioning	527.7	31,989.4	76.0	-	-	-	32,593.1
Waste Treatment	217.3	12,124.7	38,985.5	-	-	78.9	51,406.4
Erosion Control	327.5	5,223.5	-	350.4	252.9	-	6154.3
Pollination	48.1	1,182.0	-	-	71.9	-	1302.0
Biodiversity Habitat	4,171.8	83,347.8	52,643.8	-	5391.7	16.4	145,571.5
Disturbance Prevention	7,806.6	-	50,939.0	-	-	-	58,745.6
Pest Management	65.2	1067.6	-	-	97.4	-	1230.2
Nutrient Cycling	-	12,124.7	-	575.2	341.0	-	13,040.9
Aesthetics	-	-	-	251.3	176.3	6.6	434,2
Recreation	116.4	2859.6	184.0	291.0	174.0	123.2	3748.2
**TOTAL**	**14,514.3**	**159,489.4**	**145,693.5**	**4506.1**	**7744.2**	**225.1**	**332,172.6**

It is important to recall that values determined are an estimate of the ecosystem value for some services and as such, they must be considered as an order of magnitude more than a precise measurement. The results of this study must then be put in perspective and be seen as a first estimate of the value of the NCCGN's natural capital.

TABLE 15. TOTAL MINIMUM, MEAN, AND MAXIMUM VALUES FOR THE STUDIED ECOSYSTEMS

Ecosystems	Minimum Value	Mean Value	Maximum Value
	M$/yr		
Urban Forests	10.6	14.5	22.2
Rural Forests	19.9	159.5	647.8
Wetlands	145.6	145.7	145.8
Croplands	3.9	4.5	4.9
Prairies, pastures, grasslands	7.5	7.7	8.2
Freshwater Systems	0.2	0.2	0.2
**TOTAL**	**187.8**	**332.2**	**829.2**

FIGURE 9. ECOSYSTEM SERVICES VALUE OF NATIONAL CAPITAL COMMISSION'S GREEN NETWORK (VALUES EXPRESSED IN $/HA/YEAR)

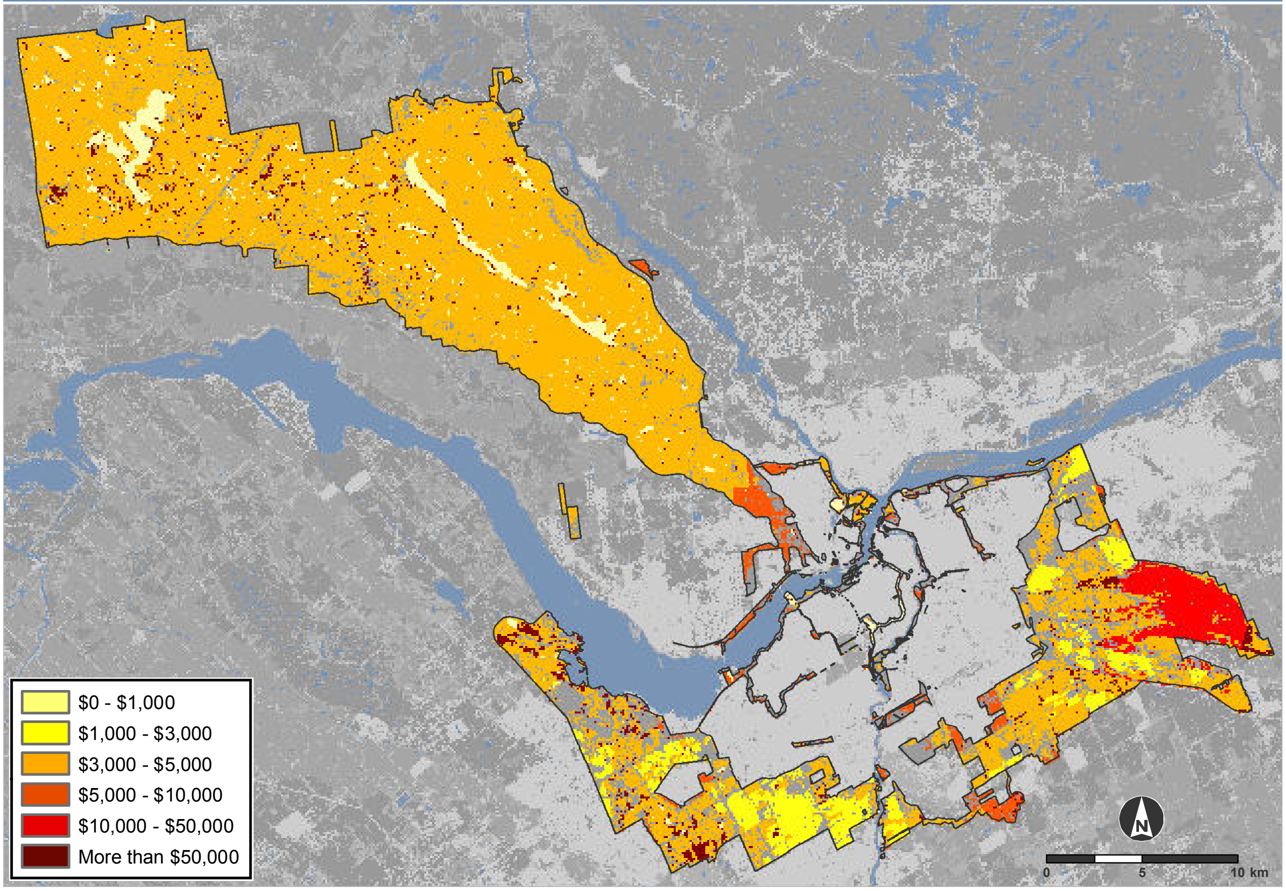

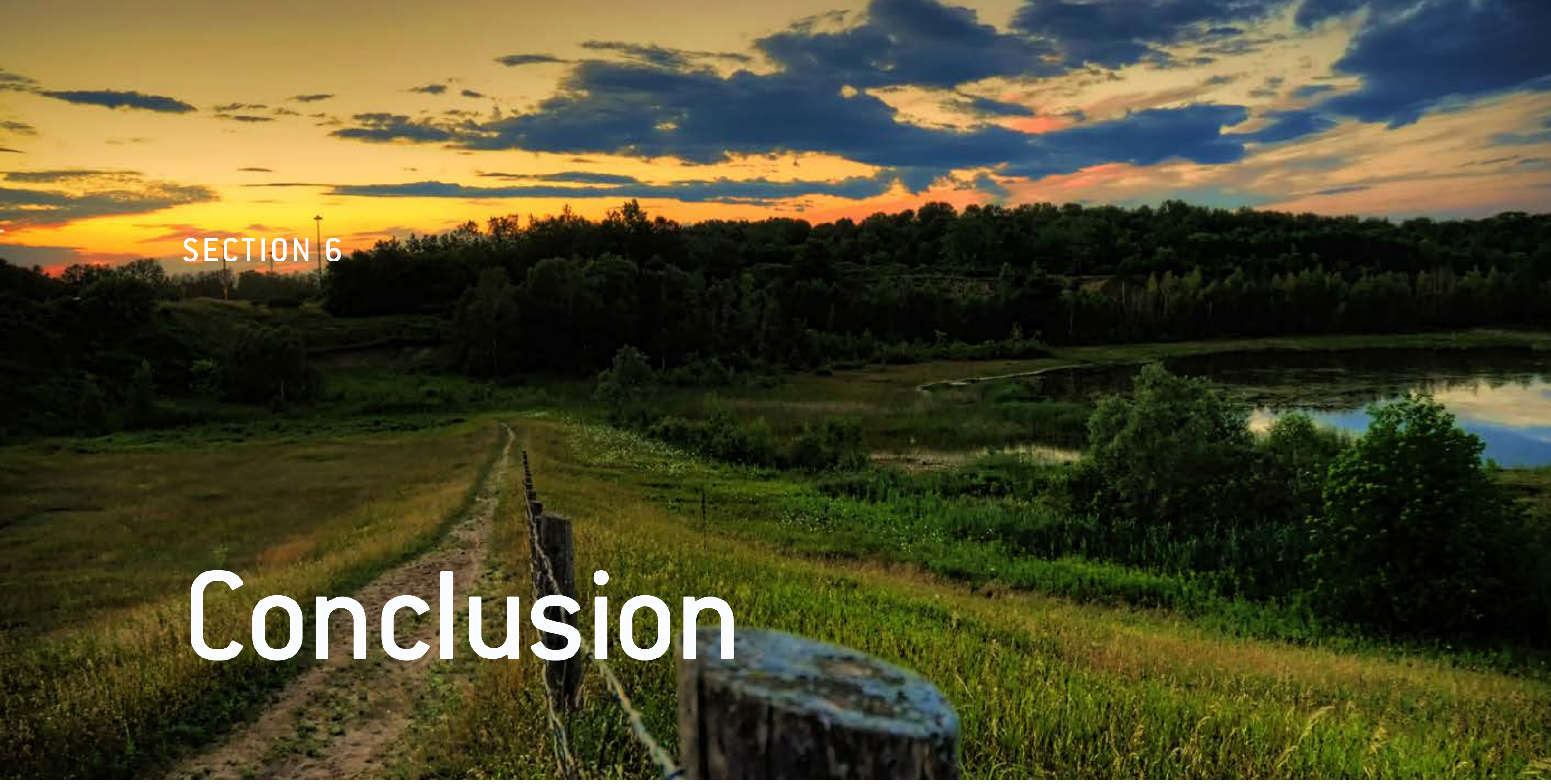

CANADA'S CAPITAL GREEN NETWORK managed by the National Capital Commission, namely the Greenbelt, Gatineau Park, and Urban Lands cover a total area of 55,000 hectares of which 72% is composed of forests and woodlands, 10% of agricultural land, 8% of urbanized land and nearly 10% of wetlands and aquatic environments.

The NCCGN provides diverse ecosystem services that have an impact on residents' well-being in the Ottawa-Gatineau metropolitan region. As described in this study, forests, agricultural lands, wetlands, prairies, pastures and grasslands, and freshwater systems provide services with a minimum value of $332 million per year, a mean of $6,026/ha/year, for a total value of greater than $5 billion. The majority of the total value is explained by non-market ecosystem services such as habitat for biodiversity, waste treatment, disturbance prevention, and global climate regulation.

Major environmental and human pressures affect the integrity of the NCCGN. They are mainly related to human activity (e.g. recreation, tourism, construction of roads, and buildings) and global change (e.g. climatic change, invasive species).

The defining feature of Canada's Capital Region is widely recognized as its green spaces. The NCC stewards 55,000 ha of natural lands and waters that sustain life and greatly enhance the quality of life in the region. While many recognize and appreciate some of the intangible benefits provided by this precious natural capital, this study enables us, for the first time, to quantify in monetary terms the vast array of benefits and services it provides. The NCC hopes that the study will enhance the ability of regional planners and decision makers to ensure that the region can continue to benefit from the ecological services for generations to come.

While many recognize and appreciate some of the intangible benefits provided by this precious natural capital, this study enables us, for the first time, to quantify in monetary terms the vast array of benefits and services it provides.

PHOTO COURTESY JANA KRIZ

References

Agriculture et Agroalimentaire Canada (2015). Gaz à effet de serre. www.agr.gc.ca/fra/science-et-innovation/pratiques-agricoles/climat/gaz-a-effet-de-serre/?id=1329321969842

Anielski, M., Wilson, S. (2005) Counting Canada's Natural Capital: Assessing the Real Value of Canada's Boreal Ecosystems. Canadian Boreal Initiative, Ottawa.

Bateman, I.J., Mace, G.M., Fezzi, C., Atkinson, G., Turner, K. (2011) Economic Analysis for Ecosystem Service Assessments. Environmental Resource Economics 48, 177–218.

Boyd, J., Banzhaf, S. (2007) What are ecosystem services ? The need for standardized environmental accounting units. Ecological Economics, 63(2-3), 616-626.

Breuste, J., Haase, D., and Elmqvist, T. (2013). Urban Landscapes and Ecosystem Services, in Ecosystem Services in Agricultural and Urban Landscapes, Edited by Steve Wratten, Harpinder Sandhu, Ross Cullen and Robert Costanza, Wiley-Blackwell, pp.83-104.

Brown, M., and Rispoli, L. (2014). Metropolitan Gross Domestic Product: Experimental Estimates, 2001 to 2009. Economic Insights, no. 042, November 2014, Statistics Canada, Catalogue no. 11-626-X no.042, 8 pages.

Centre d'Analyse Stratégique (2009) Approche économique de la biodiversité et des services liés aux écosystèmes. Rapports et documents du Centre d'Analyse Stratégique, Paris.

Ciriacy-Wantrup, S.V. (1952) Resource Conservation : Economics and Policies. University of California Press, Berkeley.

City of Ottawa (a). Geography. http://ottawa.ca/en/residents/arts-culture-and-community/museums-and-heritage/billings-family-virtual-exhibit-0-1

City of Ottawa (b). Species at risk. http://ottawa.ca/en/residents/water-and-environment/plants-and-animals/species-risk

City of Ottawa (c). Ottawa's Natural Lands. http://ottawa.ca/en/city-hall/planning-and-development/official-and-master-plans/greenspace-master-plan/2-identifying/22

Costanza, R., de Groot, R., Sutton, P., van der Ploege, S, Anderson, S., Kubiszewski, I., Farber, S. Turner, K.T. (2014). Changes in the global value of ecosystem services. Global Environmental Change, 26 : 152-158.

Costanza, R., d'Arge, R., de Groot, R., Farberk, S., Grasso, M., Hannon, B., Limburg, K., Naeem, S., O'Neill, R.V., Paruelo, J., Raskin, R.G., Suttonkk, P., van den Belt, M. (1997) The value of the world's ecosystem services and natural capital. Nature 387, 253-260.

Daily, G. (ed.) (1997) Nature's Services: Societal Dependence on Natural Ecosystems. Washington, DC: Island Press.

Daily, G.C., Polasky, S., Goldstein, J., Kareiva, P.M., Mooney, H.A., Pejchar, L., Ricketts, T.H., Salzman, J., and Shallenberger, R. (2009) Ecosystem services in decision making: time to deliver, Front Ecol Environ, 7(1), p.21-28.

De Groot, R.S., Wilson, M.A., Boumas, R.M.J. (2002) A typology for the classification, description and valuation of the ecosystems goods, services and functions. Ecological Economics 41 (3), 393-408.

Del Degan, Massé (2010). Plan de conservation du Parc de la Gatineau. www.ccn-ncc.gc.ca/sites/default/files/pubs/plan-conservation-ecosystemes-parc-Gatineau-resume-fev-2010.pdf

Ducks Unlimited (2007). Portrait des milieux humides – Région administrative de l'Outaouais (07), mars. Slides 27, 35. www.canards.ca/assets/2012/07a/PRCMH_R07_OUTA_2007_portrait_cartes.pdf

Dupras, J., Alam, M. J. Revéret. (2015) Economic Value of Greater Montreal's Non-Market Ecosystem Services in a Land Use Management and Planning Perspective. The Canadian Geographer/ Le géographe canadien. 59 (1) : 93-106.

Dupras, J., Alam, M. (2015) Urban Sprawling and Ecosystem Services: A Half-Century Perspective in the Montreal Region (Quebec, Canada). Journal of Environmental Policy and Planning 17(2) : 180-200.

Dupras, J., Michaud, C., Charron, I., Revéret, J.P. (2013). Le capital écologique du Grand Montréal : une évaluation économique de la biodiversité et des écosystèmes de la Ceinture verte. Fondation David Suzuki.

Environment and Climate Change Canada (2016). Technical Update to Environment and Climate Change Canada's Social Cost of Greenhouse Gas Estimates. http://ec.gc.ca/cc/BE705779-0495-4C53-BC29-6A055C7542B7/Technical%20Update%20to%20Environment%20and%20Climate%20Change%20Canadas%20Social%20Cost%20of%20Greenhouse%20Gas%20Estimates.pdf

Fabos, J., Ryan, R.L. (2004) International greenway planning: An introduction, Landscape and Urban Planning, 68 (2–3), pp. 143–146.

Garneau, M, van Bellen, S. (2016). Synthèse de la valeur et la répartition du stock de carbone terrestre au Québec. Centre GEOTOP et Département de géographie, Université du Québec à Montréal, Montréal, 60 p.

Gomez-Baggethun, E. and Barton, D.N. (2013). Classifying and valuing ecosystem services for urban planning. Ecological Economics, 86, pp. 235-245.

Gómez-Baggethun, E., Gren, A., N. Barton, D., Langemeyer, J., McPhearson, T., O'Farrell, P., Andersson, E., Hamstead, Z., Kremer, P. (2013) Urban ecosystem services. In T. Elmqvist M. Fragkias, et al. (Eds.), Cities and biodiversity outlook: Urbanization, biodiversity and ecosystem services: Challenges and opportunities (175–251). Netherlands: Springer.

Gordon, D., and Scott, R. (2008) Ottawa's Greenbelt Evolves from Urban Separator to Key Ecological Planning Component, in Urban Green Belts in the Twenty-first Century, Edited by Marco Amati, Ashgate, pp. 129-147.

Guerry, A.D. Polasky, S., Lubchenco, J., Chaplin-Kramer, R., Daily, G.C., Griffin, R., Ruckelshaus, M., Bateman, I.J., Duraiappah, A., Elmqvist, T., Feldman, M.W., Folke, C., Hoekstra, J., Kareiva, P.M., Keeler, B.L., Li, S., McKenzie, E., Ouyang, Z., Reyers, B., Ricketts, T.H., Rockström, J., Tallis, H. and Vira, B. (2015) Natural capital and ecosystem services informing decisions: From promise to practice, PNAS, 112 (24), June 16, 2015, pp. 7348-7355.

Haines-Young, R., Potschin, M. (2008) England's terrestrial ecosystem services and the rationale for an ecosystem approach. DEFRA, www.ecosystemservices.org.uk

He, J., Moffette, F., Fournier, R., Revéret, J.P., Théau, J., Dupras, J., Boyer, J.P., Varin, M. (2015) Meta-Analysis for the Transfer of Economic Benefits of Ecosystem Services Provided by Wetlands within Two Watersheds in Quebec, Canada. Wetland Ecology and Management 23 (4) : 707-725.

He, J., Dupras, J., Poder, T. (2016) The value of Wetlands in Quebec : a comparison between contingent valuation and choice experiment. Journal of Environmental Economics and Policy. DOI : 10.1080/21606544.2016.1199976.

Hirabayashi, S. (2014) i-Tree Canopy Air Pollutant Removal and Monetary Value Model Descriptions. https://www.itreetools.org/canopy/resources/i-Tree_Canopy_Air_Pollutant_Removal_and_Monetary_Value_Model_Descriptions.pdf

Kenny, A., Elgie, S., Sawyer, D. (2011) Advancing the Economics of Ecosystems and Biodiversity in Canada: A Survey of Economic Instruments for the Conservation & Protection of Biodiversity. Environment Canada, Ottawa.

Klumpp, K., Tallec, T., Guix, N., Soussana, J. F. (2011). Long-term impacts of agricultural practices and climatic variability on carbon storage in a permanent pasture. Global Change Biology, 17, 3534–3545.

Kurz, W. A., M.J. Apps (1999). A 70-year retrospective analysis of carbon fluxes in the Canadian forest sector. Ecological Applications, 9(2):526–547.

Lafleur, P.M., Roulet, N.T., Admiral, S. (2001) The annual cycle of CO_2 exchange at a boreal bog peatland. J. Geophys. Research, 106: 3071-3081.

Lantz, V., Boxall, P., Kennedy, M., Wilson, J. (2013) The valuation of wetland conservation in an urban/peri urban watershed. Regional Environmental Change 13 (5), 939-953.

Laurans, Y., Rankovic, A., Billé, R., Pirard, R., Mermet, L. (2013) Use of ecosystem services economic valuation for decision making: Questioning a literature blindspot, Journal of Environmental Management, 119, pp. 208-219.

Millennium Ecosystem Assessment (2005) Ecosystems and Human Well-being: Synthesis. Island Press, Washington.

Ministère du Développement durable, de l'Environnement et de la Lutte contre les changements climatiques. (2015) Portrait sommaire du bassin versant de la rivière des Outaouais.51 pages. [En ligne]. www.mddelcc.gouv.qc.ca/eau/bassinversant/bassins/outaouais/portrait-sommaire.pdf (page consultée le 11/04/2016).

Mueller, H., Hamilton, D.O., Doole, G.J. (2016) Evaluating services and damage costs of degradation of a major lake ecosystem, Ecosystem Services. http://dx.doi.org/10.1016/j.ecoser.2016.02.037i

National Capital Commission (NCC) (2005). Plan directeur du parc de la Gatineau. www.ccn-ncc.gc.ca/sites/default/files/pubs/CCN-parc-de-la-gatineau-plan-directeur-2005.pdf

National Capital Commission (NCC) (2013). Greenbelt Master Plan. www.ncc-ccn.gc.ca/sites/default/files/pubs/final-2013-greenbelt-master-plan-en.pdf

National Capital Commission (NCC) (2016). Report on Gatineau Park Ecosystems. Gatineau Park. 24 pages.

National Capital Commission (NCC) (nd). 2014-2015 Annual Report, p.49. www.ncc-ccn.gc.ca/sites/default/files/pubs/annualreport_2014-15_e_web_144dpi.pdf

National Capital Commission (NCC) (nd). NCC, Mandate. www.ncc-ccn.gc.ca/about-ncc/mandate-organization

Nowak, D., Crane, D.E., Stevens, J.C. (2006) Air pollution removal by urban trees and shrubs in the United States. Urban Forestry & Urban Greening 4 11, 5-123.

Pattison, J., Boxall, P.C., Adamowicz, W.L. (2011) The Economic Benefits of Wetland Retention and Restoration in Manitoba. Canadian Journal of Agricultural Economics/Revue canadienne d'agroéconomie 59 (2), 223-244.

Pauly, D., Zeller, D. (2016) Catch reconstructions reveal that global marine fisheries catches are higher than reported and declining. Nat. Commun. 7:10244 doi: 10.1038/ncomms10244

Poder, T., He, J., Dupras, J., Ndefo, F. (2015) La valeur économique de la Ceinture et la trame bleue du Grand Montréal. Rapport préparé pour la Fondation David Suzuki.

Rands, S.A., Withney, H.M. (2011) Field Margins, Foraging Distances and Their Impacts on Nesting Pollinator Success. PlosOne, 6(10): e25971.

Samuelson, P.A. (1954) The Pure Theory of Public Expenditure The Review of Economics and Statistics, Vol. 36, No. 4 : 387-389.

Sandhu, H., Wratten, S. (2013) Ecosystem Services in Farmland and Cities, in Ecosystem Services in Agricultural and Urban Landscapes, Edited by Steve Wratten, Harpinder Sandhu, Ross Cullen and Robert Costanza, Wiley-Blackwell, pp. 1-15.

Statistics Canada (2012) Ottawa - Gatineau, Ontario (Code 505) and Ontario (Code 35) (table). Census Profile. 2011 Census. Statistics Canada Catalogue no. 98-316-XWE. Ottawa. www12.statcan.gc.ca/census-recensement/2011/dp-pd/prof/index.cfm?Lang=E

Statistics Canada (2013) Ottawa - Gatineau, CMA, Ontario (Code 505) (table). National Household Survey (NHS) Profile. 2011 National Household Survey. Statistics Canada Catalogue no. 99-004-XWE. Ottawa. www12.statcan.gc.ca/nhs-enm/2011/dp-pd/prof/index.cfm?Lang=E

Statistics Canada (2015) Environmental and resource accounts: Frequently asked questions. Last modified 2015-08-21 www.statcan.gc.ca/eng/nea/faq/env#q4

Smith, W.N., Desjardins, R.L., Grant, B. (2001) Estimated changes in soil carbon associated with agricultural practices in Canada. Canadian Journal of Soil Science. 81:221-227.

Taylor, J., Paine, C., Fitzgibbon, J. (1995) From Greenbelt to Greenways: Four Canadian case studies, Landscape and Urban Planning, 33, pp. 47–64.

TD Economics (2014a) Urban forests: The value of trees in the City of Toronto. https://www.td.com/document/PDF/economics/special/UrbanForests.pdf

TD Economics (2014b) The value of urban forests in cities across Canada. https://www.td.com/document/PDF/economics/special/UrbanForestsInCanadianCities.pdf

TEEB – The Economics of Ecosystems & Biodiversity. Ecosystem Services (2010) www.teebweb.org/resources/ecosystem-services/

Tilman, D., Reich, P.B., Knops, J., Wedin, D., Mielce, T., Lehman, C. (2001) Diversity and productivity in a long-term grassland experiment. Science 294, 843-845.

Troy, A., Wilson, M.A. (2006) Mapping ecosystem services: practical challenges and opportunities in linking GIS and value transfer. Ecological Economics 60, 435-449.

Wilson, S. (2008) Ontario's Wealth, Canada's Future: Appreciating the Value of the Greenbelt's Eco-Services. David Suzuki Foundation, 62 pages.

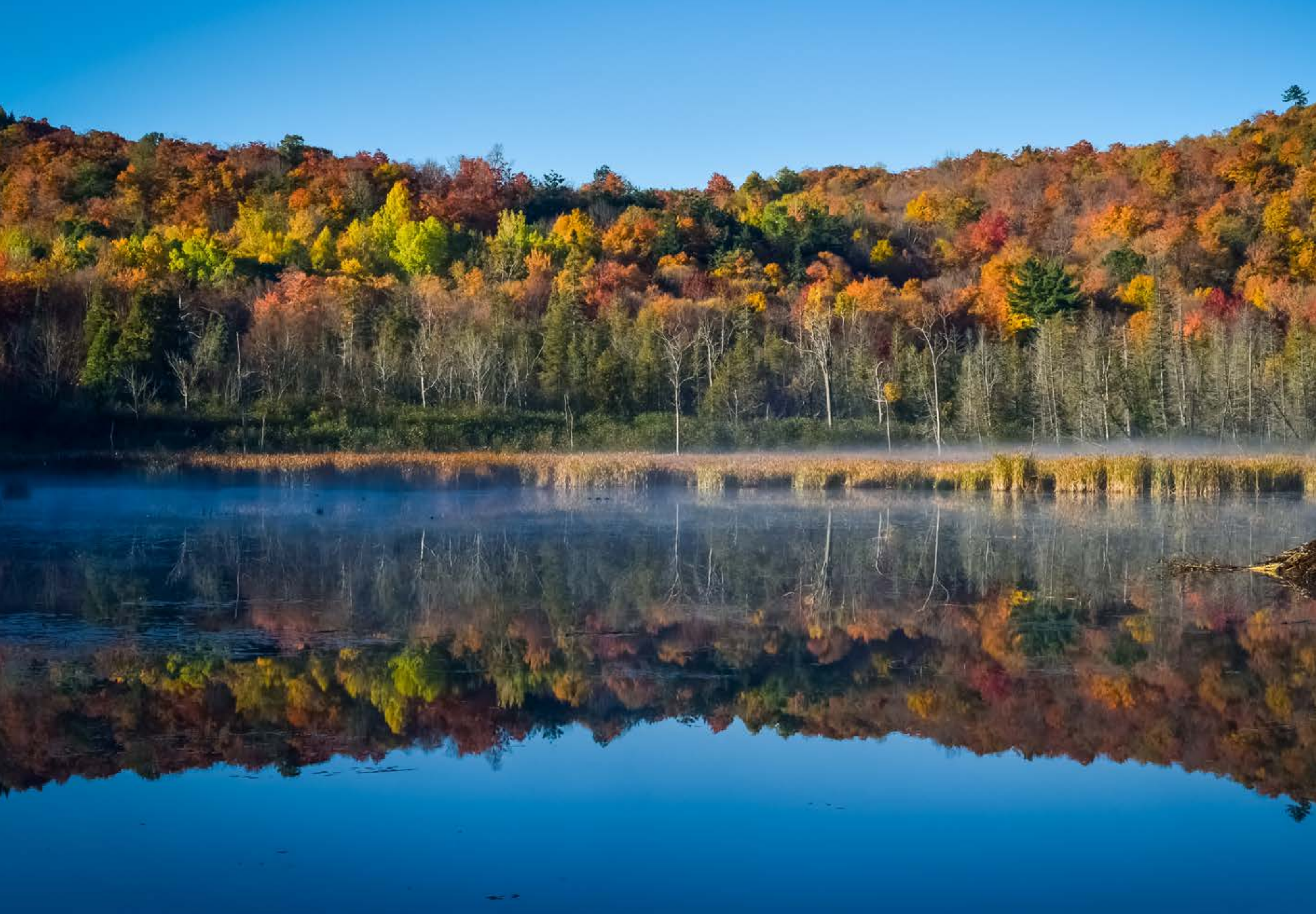

NATIONAL CAPITAL COMMISSION
COMMISSION DE LA CAPITALE NATIONALE

The National Capital Commission (NCC) is the federal Crown corporation dedicated to ensuring that Canada's Capital is a dynamic and inspiring source of pride for all Canadians, and a legacy for generations to come. Building on more than a century of experience, the NCC provides unique value in the Capital Region by fulfilling three specific roles: long-term planner of federal lands, principal steward of nationally significant public places, and creative partner committed to excellence in development and conservation.

ncc-ccn.gc.ca

David
Suzuki
Foundation

We collaborate with Canadians from all walks of life, including government and business, to conserve our environment and find solutions that will create a sustainable Canada through science-based research, education and policy work. Our mission is to protect the diversity of nature and our quality of life, now and for the future.

davidsuzuki.org

Printed on 100% post-consumer recycled paper processed without chlorine.
