# Supplementary material for: The economic value of Canada’s National Capital Green Network
Source: PLoS One. 2021 Jan 19;16(1):e0245045. doi: 10.1371/journal.pone.0245045 (PMC7815161; doi:10.1371/journal.pone.0245045)
Supplement: Attachment — Submitted filename: Review comments.docx [file pone.0245045.s004.docx]

The article starts by providing an excellent overview of the importance of ecosystem service (ES) in enabling decision-makers to consider the impact of planning on people's wellbeing. An extensive list of 13 Ecosystem services from the NCC's Greenbelt is provided. Several land uses and their spatial extent within the NCC Green network are provided. Four valuation methods are then given as the methods that will be used, however, there is no explanation as to why these are chosen for the different ecosystem services.

After this, there is missing information between the land use covers and ecosystem services from these land use covers. The different land covers provide a lot more ecosystem services but there is no mention of why only a few were considered for valuation. For example, wetlands have property price premium impact in urban areas, and this is not covered in this study.

A major issue with the paper is the limited information provided to support their estimated values. For example, in the pollination benefit is just given as $31/ha/year but there is no explanation on what the counterfactual to this estimate is and there is no information on the crops/plants that benefit from this ES. For each reported benefit estimate, more information on the original study, attributes/indicators driving value and where possible a counterfactual scenario should be provided. For example, at the moment it is not clear whether the $31/ha/year pollination ES is additional yield gains or avoided costs of alternative pollination. This needs to be addressed for all reported benefits in the results section.

A table with land-use covers, a list of ES by land use cover and valuation of methods will be a good improvement. This article has relied on previous work to inform their benefit estimation. There are many non-market valuation methods such as the choice modelling, hedonic pricing models, travel cost models which could provide robust and site-specific benefits. However, these were not used and there is no explanation as to why this is the case.

A brief paragraph on limitations is required. Study limitations must cover why some ES are not quantified and why other more common methods e.g. travel cost for visits to the forest were not used.

In summary, the article does not clearly explain their ES calculations approach, some equations will be helpful. There is also no acknowledgement or mention of the original studies in the results section where estimated values have been provided. More needs to be done to compare the study findings to what others have estimated for similar environmental assets and ES.

**Specific comments**

Line 161 – worth noting that raising taxes is relatively difficult and requires political support.

Line 168 – 172 - Apart from having a lower risk of being developed, are there any other attributes to the NCC that make it to have potentially different end-user values to the open spaces managed by municipalities? Otherwise, the economic value should not be different just because of who manages.

Line 175 – “cost” should be “replacement cost”?

Line 176 – delete “of”

Line 218 – This is the first mention of your database, explain it here or let the reader know that this is described later

Line 218 – you mention that 13 ES were selected. Explain the basis for this selection and provide information on ES that were dropped.

Line 237 – 242 – This introduction requires adjustment to re-focus on valuation methods and not just those that were used in the study. Thus, an overview of non-market valuation techniques followed by a discussion of the one used in the study plus an explanation of why they were chosen should be provided.

Line 254 – 261 what is the counterfactual for this estimate. What production crops are affected and what is the benefit being measured? Is it avoided costs of alternative pollination methods or increases in yield? These questions should be addressed. A similar approach needs to be adopted for all other benefits/ES.

Line 296 – Clarify what is meant by adjustment here. Is it the conversion of foreign currency Canadian dollars or adjusting dollar values for inflation? This should be made clear.

Line 367 (Table 2) – since the paper is about valuing ES. A table with information on Land use cover, ecosystem services and valuation will be more informative for the reader.

Results section – all your ES estimates need a clear explanation of what the estimated values are. For example, Line 421 what exactly does disturbance or flood control mean, what parameters were used to arrive at $5030/ha/year, given that this is a benefit transfer exercise what is the original source of your estimate. This source must be acknowledged as a citation. This comment applies to results in the article.

Results section – for each of the ES quantified, how do your findings compare to previous studies?

Line 596 – 598 – this is an important point. However, the study has not sufficiently supported the methods they used or provided enough information for their methods to be assessed in a meaningful way. This makes it difficult to accept that the study has satisfactorily contributed to the literate as they articulate in Line 596 – 598 (The originality of this study is to explore the value of a suite of regulating services while in many studies, these services are often left aside because of their difficult association with economic indicators.)

Figure 1: What do the different colors in this figure mean?
