# Supplementary material for: The economic value of Canada’s National Capital Green Network
Source: PLoS One. 2021 Jan 19;16(1):e0245045. doi: 10.1371/journal.pone.0245045 (PMC7815161; doi:10.1371/journal.pone.0245045)
Supplement: Attachment — Submitted filename: Response to Reviewers_PlosOne.docx [file pone.0245045.s005.docx]

**Response to Editors and Reviewers**

**[Editors](#_Toc46931596)**	[1](#_Toc46931596)

**[Reviewer #1](#_Toc46931597)**	[2](#_Toc46931597)

**[Reviewer #2](#_Toc46931598)**	[6](#_Toc46931598)

**[General comments:](#_Toc46931599)**	[14](#_Toc46931599)

# **Editors**

When submitting your revision, we need you to address these additional requirements.

1. Please ensure that your manuscript meets PLOS ONE's style requirements, including those for file naming. The PLOS ONE style templates can be found at

[https://journals.plos.org/plosone/s/file?id=wjVg/PLOSOne_formatting_sample_main_body.pdf](https://journals.plos.org/plosone/s/file?id=wjVg/PLOSOne_formatting_sample_main_body.pdf) and

[https://journals.plos.org/plosone/s/file?id=ba62/PLOSOne_formatting_sample_title_authors_affiliations.pdf](https://journals.plos.org/plosone/s/file?id=ba62/PLOSOne_formatting_sample_title_authors_affiliations.pdf)

Thank you for sharing these templates, we used them to modify our submission.

2. Please amend either the title on the online submission form (via Edit Submission) or the title in the manuscript so that they are identical.

3. Please include captions for your Supporting Information files (as the Dupras et al. 2016.pdf file has been changed from "other" to "supporting information" item type) at the end of your manuscript, and update any in-text citations to match accordingly. Please see our Supporting Information guidelines for more information: <http://journals.plos.org/plosone/s/supporting-information>.

We added the citation for the Supporting information file.

4. **Please upload a copy of Figure 1**, to which you refer in your text on page 17. If the figure is no longer to be included as part of the submission please remove all reference to it within the text.

We will add a copy of Figure 1.

**Comments to the Author**

| 1. Is the manuscript technically sound, and do the data support the conclusions?  The manuscript must describe a technically sound piece of scientific research with data that supports the conclusions. Experiments must have been conducted rigorously, with appropriate controls, replication, and sample sizes. The conclusions must be drawn appropriately based on the data presented. | |
| --- | --- |
| Reviewer #1: | Yes |
| Reviewer #2: | Partly |
| 2. Has the statistical analysis been performed appropriately and rigorously? | |
| Reviewer #1: | Yes |
| Reviewer #2: | No |
| 3. Have the authors made all data underlying the findings in their manuscript fully available? The [PLOS Data policy](http://www.plosone.org/static/policies.action#sharing) requires authors to make all data underlying the findings described in their manuscript fully available without restriction, with rare exception (please refer to the Data Availability Statement in the manuscript PDF file). The data should be provided as part of the manuscript or its supporting information, or deposited to a public repository. For example, in addition to summary statistics, the data points behind means, medians and variance measures should be available. If there are restrictions on publicly sharing data—e.g. participant privacy or use of data from a third party—those must be specified. | |
| Reviewer #1: | Yes |
| Reviewer #2: | No |
| 4. Is the manuscript presented in an intelligible fashion and written in standard English? PLOS ONE does not copyedit accepted manuscripts, so the language in submitted articles must be clear, correct, and unambiguous. Any typographical or grammatical errors should be corrected at revision, so please note any specific errors here. | |
| Reviewer #1: | Yes |
| Reviewer #2: | Yes |

5. Review Comments to the Author

Please use the space provided to explain your answers to the questions above. You may also include additional comments for the author, including concerns about dual publication, research ethics, or publication ethics. (Please upload your review as an attachment if it exceeds 20,000 characters)

**Reviewer #1**

**Reviewer #1**: This paper presents an estimation of the value of ecosystem services of forests, wetlands, croplands, prairies and grasslands, and freshwater systems in Canada’s
Capital Region (Ottawa-Gatineau region).

**Main comments:**

This is an important study which I think will be highly cited. I admire the authors’ efforts to estimate so many ecosystem services in one study. It is important that such a study is done, otherwise we will continue to grossly undervalue the ecosystem services of natural assets. Ideally, the valuation of ecosystem services should be based on original studies. However, the authors’ have appropriately dealt with data, time, and budget constraints by applying a combination of methods instead of performing original studies. I believe the methods chosen by the authors which include market pricing, cost replacement, and two benefit transfer approaches (with adjustment and with meta-analysis) for ascertaining each of the ecosystem services of natural assets were appropriate ones.

I mostly have only minor comments for this paper - mainly for clarification. My only main comment for this paper would be for the authors to make a clear distinction - in the methods section - between the value of the stock of natural assets versus the value of the flow of the ecosystem services. This is consistent with the Systems of Environmental and Economic Accounting (SEEA) Framework ([seea.un.org](http://seea.un.org)). For example, the stock value of trees would be based on the replacement cost of trees, which is different from the value of the flow of ecosystem services that trees provide e.g. carbon sequestration, storm water retention. If you don’t include the value of the stock of the asset, you will significantly under estimate its value.

I hope my comments will help improve the quality of this manuscript further.

**Response**
Thank you for all of your comments and suggestions.

More specifically, to take into account the comment with regard to the distinction between stock and flow of resources, we added the following paragraph in section 3:

“There is distinction to be made between the evaluation of stocks of natural resources and of flow of ecosystem services. Whereas the stock value of a wetland would be its replacement cost, the flow of the services provided by wetlands include flood prevention, carbon sequestration and habitat for biodiversity. In this study, we did include the values of the stock of wetlands, as the meta-analysis by He et al. [32] provides the value of the stock of one ha. When it comes to carbon storage, the value of the stock of carbon was taken into account for the service of Global climate regulation for Forests and for Wetlands. However, the value of the stock of capital was not included in the value of the other ES.”

We also added two sentences in our limitations paragraph in the Discussion:

“Finally, our analysis was not exhaustive with regard to the valuation of the stock of natural capital, where only the values for Wetlands ES included the stock of natural capital and the values for the service of Global climate regulation included the value of the stock of carbon. This suggests that our estimate of the value of the NCC’s Green network is an underestimation.”

**Minor comments:**

1. P6 Line 128: The description of the study site really needs a figure or a map to really help the readers who are not familiar with the area to get a good idea of what the land use composition looks like. Perhaps place the figure about here on P6.

**Response**: Our Figure 1 shows the land use in the Green network, but to account for your comment, we added an element to show where this region is located in the Canadian landscape. We understand that this Figure was not properly uploaded at the time of our submission, so we will remediate to the situation at this time.

2. P9 Line 203-205: It seems unusual that you have to make assumptions around population density to distinguish between urban and rural areas. I would have thought that Canada would have some sort of land zoning system that would describe the type of land use whether it is urban or rural.

**Response**: Yes, Canada does use a land zoning system, however in our dataset the divisions are made at the census level, which often includes urban and rural areas. This is why we used Statistics Canada’s definition of urban and rural areas to define our cells.

3. P12 Line 260-261: How were the rates of dependence on pollinators calculated? Please describe the method, or provide a citation for the method.

**Response:** The rates of dependence on pollinators were not calculated; we took the rates provided by Morse and Calderone (2000) as reported by AAC (2017). The method for the estimation of the pollination service is being explained in more detail in the Market pricing subsection.

“The economic valuation of the pollination service was based on the method described in Winfree et al. [53], where the value of the pollinating service from wild pollinators, in this case, is based on the value of production. As our focus was on wild pollinators, we used the hypothesis from the study by Rands and Whitney [54] which studies the effect of wild field hedges for nesting pollinators and their travel capacity which varies from 125 m to 2 km. As a conservative distance, we chose to consider the distance of 1 km that pollinators can travel towards agricultural lands from nesting sites. By doing so, we estimated the pollinating potential of wild field hedges (forests and prairies and grassland) on the croplands. This was done by counting the area in a 1 km buffer around the crops dependent on pollination that contains is made of forests and prairie or grassland (surface_FPG_). The equation we used is the following:

V_pollination_ = ((P * Y – C)* D *ρ) * surface_FPG_	(1)

Where V_pollination_ is the value of the pollination service, P is the price of the crop, Y is the yield, C are the costs of production, D is the crop dependency on insect pollinators, and ρ is the percent reduction in insect pollinators [53]. In our case, we assumed that the value of ρ is 1. We limited our estimation to crops that have a dependence on pollinators. We used the value of the net benefit (P*Y – C) from beans, berries, soy, and forage as calculated for the provisioning service, and took into account their surface area and their rates of dependence on pollinators. We used the degree of dependence as reported by Morse and Calderone [55] [56] for berries/strawberries (0.2), soy (0.1), and forage (1). For beans, although the degree of dependence was not provided in Morse and Calderone [55], we assumed a degree of dependence of 0.1, as it is a flowering crop.”

4. P12 Line 262-265: I’m concerned that the recreational value is a gross under estimation of the true recreational value. And this value is being used to estimate the recreational value for both forests and wetlands. Would it be possible to capture visitor statistics and estimate the travel cost of visitors based on how far they have to travel to the site? This should only require the number of visitors per year and the post code of their residential address.

**Response:** You are very right to point out the fact that our recreational value is a gross underestimation. We would have liked to do a travel cost analysis, but we do not have access to the postal codes of visitors. At the Gatineau Park and in other green infrastructures of the Green network, access is usually free and few people purchase passes, mainly to access the park in the winter. However, there was a study conducted in 2015-2016 by Environics, a firm specialized in surveys. They did not perform a true travel cost analysis, but they did report the amount of money spent by visitors of the Gatineau Park in the region ($184M). As a result, we will change our value for the recreation ES to reflect this spending instead of simply using the entrance fees ($2.7M). We recognize that this value may still be an underrepresentation of the true recreational potential of the Green Network as it only focuses on the Gatineau Park, but it is more interesting than our previous estimate.

5. **Table 1:**
   -For clarification, please specify what is the dependant variable (including units e.g. $/year, etc)?

**Response**: The value of the dependent variable is the natural logarithm of the value of the stock of 1 ha of wetland ($/ha).

6. If I interpret the parameter value of [ln wetland size] correctly, its saying that the larger the wetland the lower the ecosystem services value. Please explain why this is the case for this study site. I would have expected a positive and/or quadratic parameter.

**Response**: In He et al.’s (2015) paper, they explain that the wetland size has a decreasing marginal utility, where the larger the wetland, the smaller the new value of an additional hectare of wetland. In this sense, the value of [ln wetland size] means that as the size of a wetland increases, the ecosystem services value does not increase in the same manner.

7. P20 Line 407: Please explain why the pollination value of urban and rural forests are assumed to be the same - at $31/ha/year. I would have expected them to be different.

**Response**: The value of the pollination service was estimated by dividing the value of production of crops that are dependent on pollination by the area in the 1 km buffer zone that is forested or a prairie or pasture. This hypothesis was based on idea that wild pollinators can establish their nesting area in these land uses and then travel 1 km (125m to 2 km – Rands and Whitney 2011) to croplands. In this sense, we did not distinguish between land uses.

8. P21 Line 428-429: Please provide citations for the ‘three monetary estimates’ of nutrient cycling.

**Response**: As you will see in the reviewed version of the manuscript, we have added all of the sources that were used for the benefit transfer analysis with adjustment in Table 2 and in S.1 Table.

9. P24 Line 494: It would be good if you could cite the studies where the values used in the benefit transfer method came from - unless they all came from He et al.

**Response:** As you will see in the reviewed version of the manuscript, we have added all of the sources that were used for the benefit transfer analysis with adjustment in Table 2 and in S.1 Table.

10. Table 9: Please add a column to specify the area (in ha) for each of the natural assets.

**Response:** We added a row, after the total, to specify the area (in ha) for each of the natural assets.

11. Figure 1: I was not able to see Figure 1 on the PDF copy that I have therefore I cannot comment.

**Response**: Sorry about this situation, it will be corrected in this revision.

12. Figure 2: You should put units in the legend of Figure 2.

**Response:** Thank you for your comment, we added the units in the legend title of Figure 2.

# **Reviewer #2**

**Reviewer #2**: The article start off by providing an excellent overview on the importance of ecosystem service (ES) in enabling decision makers to consider the impact of planning on people's well being. An extensive list of 13 Ecosystem services from the NCC's Greenbelt is provided. Several land uses and their spatial extent within the NCC Green network are provided. Four valuation methods are then given as the methods that will be used, however there is no explanation as to why these are chosen for the different ecosystem services.

After this there is a missing information between the land use covers and ecosystem services from these land use covers. The different land covers provide a lot more ecosystem services but there is no mention of why only a few were considered for valuation. For example wetlands have property price premium impact in urban areas, and this is not covered in this study.

A major issue with the paper is the limited information provided to support their estimated values. For example in the pollination benefit is just given as $31/ha/year but there is no explanation on what the counterfactual to this estimate is and there is no information on the crops/plants that benefit from this ES. For each reported benefit estimate, more information on the original study, attributes/indicators driving value and where possible a counterfactual scenario should be provided. For example, at the moment it is not clear whether the $31/ha/year pollination ES is additional yield gains or avoided costs of alternative pollination. This needs to be addressed for all reported benefits in the results section.

A table with land use covers, a list of ES by land use cover and valuation of methods will be a good improvement. This article has relied on previous work to inform their benefit estimation. There are many non-market valuation methods such as the choice modelling, hedonic pricing models, travel cost models which could provide robust and site specific benefits. However, these were not used and there is no explanation as to why this is the case.

A brief paragraph on limitations is required. Study limitations must cover why some ES are not quantified and why other more common methods e.g. travel cost for visits to forest were not used.

In summary, the article does not clearly explain their ES calculations approach, some equations will be helpful. There is also no acknowledgement or mention of the original studies in the results section where estimated values have been provide. More needs to be done to compare the study findings to what others have estimated for similar environmental assets and ES.

**Response to general comments**:

Firstly, we would like to thank you for all of your comments.

We have responded more specifically to your comment about the relationship between land cover and the methods used in your specific comment #6, and to your comment about the importance of naming our sources in your comment #11.

About our choice of methods, we realize that we could have been clearer. Our choice of methods was based on time and resources constraints. To calculate such an important number of ES in one study and in a short period, we needed to use approaches that were not too resource intensive. Also, we wanted to provide the NCC with values that showcases the importance of ES to the Ottawa-Gatineau region, as part of a case to keep protecting these natural areas, and not to provide specific values on each ES. This was more an exercise designed to raise awareness. To clarify this in the text, we added a sentence in the Materials and methods section:

“This choice of methods was based in part on time and resource constraints, and in part on the purpose of this study, which was to raise awareness on the general importance of ES in the NCC’s Green network.”

A brief paragraph on limitations was already included in the discussion section (the second to last paragraph).

“These results should nevertheless be considered in light of methodological limitations inherent to the approach chosen in this study, especially with regard to the spatial analysis and the economic valuation. Data availability was a limiting factor in both the spatial analysis and the economic valuation. Although tests were made to detect errors in the GIS layer, the chosen source only had an 85% precision. Data availability also impacted the economic valuation, especially when using the benefit transfer approach. Even though care was taken to avoid transfer bias, the results are not as robust as a study based exclusively on primary data. Additionally, the benefit transfer analysis was dependent on published literature, for which the coverage of ecosystems and ES is unequal. This unequal attention affects the accuracy of some monetary estimates. For example, there were more studies on forests and wetlands than on freshwater and agricultural lands. [Finally, our analysis was not exhaustive with regard to the valuation of the stock of natural capital, where only the values for Wetlands ES included the stock of natural capital and the values for the service of Global climate regulation included the value of the stock of carbon. This suggests that our estimate of the value of the NCC’s Green network is an underestimation.]^[1]^”

Despite the presence of this paragraph, we do recognize that a greater acknowledgement of the limits of our analysis can be made clearer in the text. We believe that such an acknowledgement will be made clearer with Table 1 that replace the list of ES valued.

**Specific comments (reviewer 2)**

1. Line 161 – worth noting that raising taxes is relatively difficult and requires political support.

**Response**: Yes, this is a good point. In order to nuance the initial statement, we have added the following sentence on Lines 164-165:

“Indeed, it is often politically damaging for municipal governments to raise new taxes or to increase the taxation level of citizens.”

2. Line 168 – 172 - Apart from having a lower risk of being developed, are there any other attributes to the NCC that make it to have potentially different end-user values to the open spaces managed by municipalities? Otherwise, the economic value should not be different just because of who manages.

**Response**:

Yes, the economic value should not be different because the lands are managed by the NCC, but this study was started in 2015 when the Canadian Premier was Stephen Harper. At this time, the budget allocated to the NCC was less important and it seemed a good idea to present the case for maintaining the current land area of the Green Network through an economic values lens.

To reflect this issue of political pressure, we have adapted the text on Lines 165 – 171:

“The case of the NCC’s green network is singular in the sense that the largest green network in the Ottawa-Gatineau region is not managed by the municipalities, but by an agency of the federal government that is not influenced by the same pressures. It can, however, be indirectly influenced by political pressures, through budget allocation and changes to its mandate. In this case, assessing the economic value of the lands preserved by the NCC, can help to showcase the importance of natural capital to human well-being in the region.”

3. Line 175 – “cost” should be “replacement cost”?

**Response**: Yes, thank you for noticing this. We changed “cost” to “replacement cost”.

4. Line 176 – delete “of”

**Response**: ok.

5. Line 218 – This is the first mention of your database, explain it here or let the reader know that this is described later

**Response**: As suggested, we have added a description of the database at its first mention.

“Our selection was also based on our own database. This database was first developed as part of a literature review by Dupras [35] which included studies of ecosystem services valuation on forest, wetland and agricultural ecosystems. In the context of this study, the database was supplemented with a literature review of the economic valuation exercises of ES that had been undertaken in Quebec and Ontario [e.g. 9, 18, 36, 37] and with studies on the valuation of aquatic ecosystems. The selected studies from this database are presented in S1 Table in the Supporting information.”

6. Line 218 – you mention that 13 ES were selected. Explain the basis for this selection and provide information on ES that were dropped.

**Response**:

The selection of the ES was based on whether or not our database included at least one estimate for the value for the given ecosystem. In some cases, we were able to include additional ES, because of data availability, as in the case of agricultural products, for example.

To clarify our rationale for only discussing these ES, we added a justification in the text.

“13 ES were selected for further analysis in the context of the NCC’s Green Network. Although the ecosystems under study generate more ecosystem services, we limited our analysis to the ecosystem services whose value had been estimated previously and to ecosystem services for which we could estimate the value using the data available in the allotted time.”

To outline the caveat that there are ES generated by the ecosystems that are not presented in the study, we changed our presentation of the list of ES on Lines 235-252. In the new table, where no data was available, we added a hollow dot.

Table 1. Selection of ES based on ecosystems and data available.

| Ecosystem services | Ecosystems / Land uses | | | | | |
| --- | --- | --- | --- | --- | --- | --- |
|  | Urban forest | Rural forest | Wetlands | Cropland | Prairie and grassland | Freshwater |
| Provisioning services | | | | | | |
| Agricultural products |  |  |  | ● | ● |  |
| Other food | ○ | ○ | ○ |  |  | ○ |
| Raw material | ○ | ○ | ○ | ○ | ○ | ○ |
| Genetic diversity | ○ | ○ | ○ | ○ | ○ | ○ |
| Regulating services | | | | | | |
| Global climate regulation | ● | ● | ● | ○ | ● | ○ |
| Air quality | ● | ● | ○ | ○ | ○ | ○ |
| Water provisioning | ● | ● | ● | ○ | ○ | ○ |
| Waste treatment | ● | ● | ● | ○ | ○ | ● |
| Erosion control | ● | ● |  | ● | ● | ○ |
| Pollination | ● | ● | ○ | ○ | ● | ○ |
| Habitat for biodiversity | ● | ● | ● | ○ | ● | ● |
| Disturbance / natural hazards prevention | ● | ● | ● | ○ | ○ | ○ |
| Pest management | ● | ● | ○ | ○ | ○ | ○ |
| Nutrient cycle | ● | ● | ○ | ● | ● | ○ |
| Cultural services | | | | | | |
| Aesthetics (landscape) | ○ | ○ | ○ | ● | ● | ○ |
| Recreational activities and tourism | ● | ● | ● | ● | ● | ● |
| Scientific and educational | ○ | ○ | ○ | ○ | ○ | ○ |
| Spiritual | ○ | ○ | ○ | ○ | ○ | ○ |
| Heritage and cultural identity | ○ | ○ | ○ | ○ | ○ | ○ |

7. Line 237 – 242 – This introduction requires adjustment to re-focus on valuation methods and not just those that were used in the study. Thus, an overview of non-market valuation techniques followed by a discussion of the one used in the study plus an explanation of why they were chosen should be provided**.**

**Response:**

Thank you for your comment. To explain why we chose these methods over others, we moved a sentence from section 3 to section 3.3.

We also added a paragraph on section 3.3 to provide more information on non-market valuation methods, as you suggested. In any case, this inclusion is useful, as some of the studies that we use to obtain the values as part of the benefit transfer method (using the database) also use stated and revealed preference methods. Since we will add more detail about the methods used in studies that we rely upon to obtain the values per ecosystem service, it will be helpful to the reader.

“The analysis of the 13 ES mentioned above was carried out using four methodological approaches. The market pricing method was used to determine the economic value of agricultural products, pollination, and recreational activities and tourism. For all of the other non-market ES, the benefit transfer approach with adjustment, the benefit transfer method with meta-analysis, and the replacement cost method were used. [Considering the large number of ecosystems that compose the NCC’s green network and the quantity of ES that they produced, the use of these methods enabled us to perform the analysis given resource constraints (time, especially), and carry out the main objective of this study. These methods will be discussed in the subsections below. ] *taken from section 3.*

In the case where we would have had time to calculate a value that is more specific to our study area, we could have used other methods. Indirect valuation methods include stated and revealed preference approaches. Revealed preference approaches are based on observed behavior [38]. They include the travel cost method, where the value of recreation, for example, is estimated as a function of the distance traveled to a location and of the purchases made on the way to and at the location [39]. They also include the hedonic pricing method, where the aesthetics ecosystem service, for example, can be estimated based on the premium associated to the value of a house that is located in a specific landscape [39]. The stated preference approaches are based on stated behavior, generally gathered as part of a survey, where hypothetical scenarios are presented to individuals. They include contingent valuation and choice experiment methods, where respondents are asked, for example, how much they are willing to pay to preserve an aesthetically pleasing landscape (contingent valuation) or are asked to choose their favorite option of a recreational area, where each scenario is composed of a number of given attributes (choice experiment) [40]. For more information on the methods and suggestions about the most appropriate ones to use in a specific context, we suggest consulting the Ecosystem Services Toolkit [41] or the TESSA [42].”

8. Line 254 – 261 what is the counterfactual for this estimate. What production crops are affected and what is the benefit being measured? Is it avoided costs of alternative pollination methods or increases in yield? These questions should be addressed. A similar approach needs to be adopted for all other benefits/ES.

**Response**:

We used the value of production from wild pollination to estimate the value of this service. To account for your comment and provide information about the way this service was calculated, we changed the paragraph starting on Line 254 to the text below.

As for the other services, we also added more information about the methods used in the primary studies when the benefit transfer method was used in a table in the Appendix as well as in Table 2. We also added a discussion on how it compares to other values when the values do not come from benefit transfer.

“The economic valuation of the pollination service was based on the method described in Winfree et al. (2011), where the value of the pollinating service from wild pollinators, in this case, is based on the value of production. As our focus was on wild pollinators, we used the hypothesis from the study by Rands and Whitney [47] which studies the effect of wild field hedges for nesting pollinators and their travel capacity which varies from 125 m to 2 km. As a conservative distance, we chose to consider the distance of 1 km that pollinators can travel towards agricultural lands from nesting sites. By doing so, we estimated the pollinating potential of wild field hedges (forests and prairies and grassland) on the croplands. This was done by counting the area in a 1 km buffer around the crops dependent on pollination that contains is made of forests and prairie or grassland (surface_FPG_). The equation we used is the following:

V_pollination_ = ((P * Y – C)* D *ρ) * surface_FPG_

Where V_pollination_ is the value of the pollination service, P is the price of the crop, Y is the yield, C are the costs of production, D is the crop dependency on insect pollinators, and ρ is the percent reduction in insect pollinators (Winfree et al. 2011). In our case, we assumed that the value of ρ is 1. We limited our estimation to crops that have a dependence on pollinators. We used the value of the net benefit (P*Y – C) from beans, berries, soy, and forage as calculated for the provisioning service, and took into account their surface area and their rates of dependence on pollinators. We used the degree of dependence as reported by Morse and Calderone (2000, from AAC 2017) for berries/strawberries (0.2), soy (0.1), and forage (1). For beans, although the degree of dependence was not provided in AAC (2017), we assumed a degree of dependence of 0.1, as it is a flowering crop.”

9. Line 296 – Clarify what is meant by adjustment here. Is it the conversion of foreign currency Canadian dollars or adjusting dollar values for inflation? This should be made clear.

**Response**: To clarify what we meant by adjustment, we added the following sentence after Line 296:

“When the values were in a currency other than Canadian dollars, we performed a first adjustment using Purchasing Power Parity (OECD), then we adjusted all values in Canadian dollars using the inflation rate (Bank of Canada).”

10. Line 367 (Table 2) – since the paper is about valuing ES. A table with information on Land use cover, ecosystem services and valuation will be more informative for the reader.

**Response:** To make Table 2 more informative, we modified it. It now provides the list of ES per Land use cover with the valuation method, and the number of ha per Land Use. When we used the benefit transfer with adjustment method, we also added the number of values used, the sources and the methods used in the primary studies.

11. **Results section** – all your ES estimates need a clear explanation of what the estimated values are. For example, Line 421 what exactly does disturbance or flood control mean, what parameters were used to arrive at $5030/ha/year, given that this is a benefit transfer exercise what is the original source of your estimate. This source must be acknowledged as a citation. This comment applies to results in the article.

**Response:** Thank you for your comment. To better acknowledge all of our sources, we added references to primary sources in the text, as well as in Table 2 and in Table S.1. We also added more information about the meaning of different ecosystem in the text.

12. Results section – for each of the ES quantified, how do your findings compare to previous studies?

**Response:**

Thank you for your suggestion. We decided to compare our results for values that did not come from the benefit transfer with adjustment method, as these already reflect the values generated from other studies. We added these comparison in the results section.

13. Line 596 – 598 – this is an important point. However, the study has not sufficiently supported the methods they used or provided enough information for their methods to be assessed in a meaningful way. This makes it difficult to accept that the study has satisfactorily contributed to the literate as they articulate in Line 596 – 598 (The originality of this study is to explore the value of a suite of regulating services while in many studies, these services are often left aside because of their difficult association with economic indicators.)

**Response:**

In line with your comment, we changed the formulation of these line to reflect the fact that we aimed to achieved this and that regulating services are important to take into account in ES valuation studies.

“The aim of this study was in part to explore the value of a suite of regulating services, since in many studies, these services are often left aside because of their difficult association with economic indicators.”

14. Figure 1: What do the different colors in this figure mean?

**Response**: The colors refer to land uses. We added a legend to this figure so that it becomes clear to the reader.

# **General comments:**

When we first performed the benefit transfer analysis with adjustment, we did not use the right reference year for values that came from the study by Dupras et Alam (2014). Although the values were presented in Canadian dollars from 2010, we used the year 2013 as a starting point to convert the values in 2015 $ CAD. As such, the pest management/biological control service urban forests and for prairie and grasslands were changed from 42$/ha/year to 45$/ha/year. For croplands, the erosion control service was changed from 106$/ha/year to 109$/ha/year, the nutrient cycling service changed from 174 to 184$/ha/year, and the recreation service changed from 88 to 94$/ha/year. Finally, for prairie and grasslands, we also adjusted the ES of habitat for biodiversity from 2324 to 2467$/ha/year. This change has an impact on the total economic value, but the biggest change to this value comes from the fact that we used a new data to estimate the recreational value, in line with Reviewer 1’s suggestion. This value was not initially available, since the publication of the report occurred after we were done with this initial study. However, it better represents the recreational value of ecosystems in the NCC Green network, which is why we believe it is interesting to change our initial value estimate to this one. In addition, some of the initial values have been changed when using the benefit transfer with adjustment method due to the rounding of values.

1. New sentences, in line with a comment from Reviewer 1.
