# Supplementary material for: The economic value of Canada’s National Capital Green Network
Source: PLoS One. 2021 Jan 19;16(1):e0245045. doi: 10.1371/journal.pone.0245045 (PMC7815161; doi:10.1371/journal.pone.0245045)
Supplement: Attachment — Submitted filename: Review comments PlosOne_5 octobre 2020.docx [file pone.0245045.s006.docx]

Review comments PlosOne

Please use the space provided to explain your answers to the questions above. You may also include additional comments for the author, including concerns about dual publication, research ethics, or publication ethics. (Please upload your review as an attachment if it exceeds 20,000 characters)

Reviewer #1: Additional comments

The authors have made substantial improvements to the manuscript. I just have a few more questions and requests that I would like the authors to respond to in order to clarify their methods and assumptions further.

1. Is it possible to draw (e.g. using dotted lines) where Gatineau Park and the Greenbelt are in Figure 1?

**Answer**: Unfortunately, we do not have the exact limits of the Gatineau Park, and this information is not available on the web in a format that we can use in ArcGIS. In that case, although we have the limits of the Greenbelt, it may be preferable not to include this information in the map.

2. Is Figure 2 mislabelled? I do not see any land use that is in gray.

**Answer**: No, the features in gray in Figure 2 represent land that we did not include in our analysis, for example land outside of the study area and urban lands. We included a caption in the legend to specify this in Figure 2.

3. The authors said that “ρ is the percent reduction in insect pollinators [53]. In our case, we assumed that the value of ρ is 1”. If you assumed in your formula Vpollination = ((P * Y – C)* D *ρ) * surfaceFPG that ρ=1, I just wanted to confirm whether ρ is equal to 1%, or is ρ equal to 100%”.

**Answer**: Thank you for mentioning this. We initially used a value of 100% for ρ, but in the formula this is expressed in decimals. However, considering the comments and suggestions of Reviewer 2 (#2 and 5), we have changed this value. There is now a rho value for specific crop types and we used the inverse of the value provided by Morse and Calderone [55]. We have also used half of these values in urban areas, to reflect the decrease in the number of native pollinators, following the results by Fetridge et al. (2008).

4. In your response to my question regarding the dependant variable of the explanatory variables described in Table 2 - you said the dependant variable is “the natural logarithm of the value of the stock of 1 ha of wetland ($/ha)”. However, in your manuscript, above Table 2, you wrote “The explanatory variables (value of the natural logarithm of the stock of natural capital”. I just wanted to confirm that this is correct because it means that you have an equation that has a natural log on both the LHS (i.e. the dependant variable) and the RHS (i.e. the explanatory variables) of the equation.

**Answer**: You are right, this is a mistake. Thank you for noticing. This is the text that should have been written in the first place. We also added the equation that is used to perform the analysis.

“The explanatory variables and their associated coefficients used as part of this meta-analysis are presented in Table 2. Equation (1) was used to calculate values of the ES. The values of the constant â and of the coefficients $\hat{b}var$ had been estimated by the meta-analysis [30].

$\hat{Y}j=\exp\left( \hat{a} \right)*\exp\left( \hat{b}servXservj \right)*\exp\left( \hat{b}wXwj \right)*\exp\left( \hat{b}geoXgeoj \right)*\exp\left( \hat{b}ecoXecoj \right)*exp(\hat{b}typeXtypej)$	(1)

In the equation, j refers to each wetland that was assessed. The dependent variable (Ŷ) is represented by the value of the natural logarithm of the stock of natural capital of 1 ha of wetland.”

5. I think its best if the authors formally explain the differences between the stock and flow of ES. The definition provided by Jones et al. (2016) is clear and accurate and perhaps you can use a definition along these lines. Jones et al. said “Natural capital has been variously defined as the stock of physical assets in the environment (water, trees, minerals, species, etc.), but also the processes (e.g. water purification, climate regulation) from which we obtain benefits (e.g. NCC, 2013).”

References

L. Jones, L. Norton, Z. Austin, A.L. Browne, D. Donovan, B.A. Emmett, Z.J Grabowski, D.C. Howard, J.P.G. Jones, J.O Kenter, W. Manley, C. Morris, D.A. Robinson, C. Short, G.M. Siriwardena, C.J. Stevens, J. Storkey, R.D. Waters, G.F. Willis. (2016). Stocks and flows of natural and human-derived capital in ecosystem services, Land Use Policy, 52 (March 2016): 151-162, <https://doi.org/10.1016/j.landusepol.2015.12.014>.

**Answer**: Thank you for this suggestion. We adjusted the text to attempt to make this distinction clearer.

“There is a distinction to be made between the evaluation of stocks of natural resources and of flow of ES. For Jones et al. [2016], natural capital encompasses the ‘stocks’, which represent assets found in the environment, and the processes through which humans perceive benefits, which can be regarded as ‘flows’ or as transformations or evolutions of the stocks [Jones et al. 2016: 154].”

Additional information for Reviewer 1: We went back with our initial estimate of the recreational ecosystem service. As pointed out by Reviewer 2 in its comment #1, using the value provided by the Environics report does not provide a marginal value for the recreational service. As a result, we decided to go back to our initial estimate, based only on access fees, even though we recognize it can only provide a partial value. Nevertheless, this value can be directly tied to recreational activities carried out in the Gatineau Park, which is why we believe it is more suitable in the context of this study.

Reviewer #2: This authors are attempting to publish a potentially very important paper on the non-market valuation of environmental assets. They do cover a broad range of ecosystem services. Unfortunately, some of the approaches used are either not well explained or potentially not appropriate.

For this paper to have a positive impact to readers, practitioners and/or researchers the authors should ensure that all their quantification approaches are suited to the benefits they are attempting to value. Where possible, equations and descriptions for all variables used in the estimation process should be well documented. Another important point is that at times the paper is written as if it must be read in conjunction with multiple previous studies. However, once the estimation approaches and variables are explained, this will not be a problem as the paper will be detailed and informative enough.

Below are my specific comments on the new version of the paper.

1. Page 4 of 147 point #4
The proposed method here is a cause of concern. While the money is spent by park visitors this money is not a reflection of the economic value of the park. The economic value of the park should be the main focus if we are seeking to put monetary values to ecosystem service. In the absence of the right data to perform a travel cost model type of analysis, a benefit transfer approach from a similar study will be your next best option for valuing the recreation ecosystem service/benefit. By using the money spent by visitors in the region, you are potentially overstating the economic value because (1) you are using gross expenditure values, (2) do not account for or attribute any of the expenditure to other nearby attractions/activities that people engage in and pay for but these are not necessarily park-based. I would suggest that you do a consumer surplus benefit transfer approach for recreation.

**Answer**: Taking your comment into account, we have decided to go back to our previous estimate of the value of the recreation service, as it relied only on money spent to perform some recreational activities in the Park.

“The economic value associated to recreational activities and tourism was estimated based on results from the NCC’s 2014-2015 Annual Report [89] which showed that the NCC collected $2.7 million in user fees, which is equivalent to $75/ha/year when the $2.7 million value is divided over the Gatineau Park’s entire spatial area (36,161 ha). These user fees correspond to annual passes used to access the park during the winter to perform cross-country skiing and snowshoeing, to camping fees and to parking fees within specific areas of the park. When we compare this value to a report by Environics [27], which showed that Gatineau park visitors (77% of residents and 23% of non-residents) spent $184 million in the region as part of their travel to the Gatineau Park in food, sport supply and other purchases such as gas, the $2.7 million in user fees seems quite low. As a result, the value of $75/ha/year is most likely insufficient to represent the real value of the recreational potential of the Gatineau Park. It is nevertheless the only value that we can directly tie to activities performed in the Park.”

2. P8 of 147 point #8
Your pollination ES quantification approach implies that without the park the nearby agricultural activities will not exist, that is there will be no production? Is this the right counterfactual for you case study areas, is it possible that agricultural production could still exist will lower native pollinators? In short, the agricultural value of production is potential erroneous without a clear and relevant counterfactual.

**Answer**: Using the production value method, the value of pollination is estimated with the assumption that there is no substitute pollinators, from honey bees or other pollinators in this case. However, it does not mean that there will be no production, for most crops, because we take into account the level of crop dependency on crop pollination (D). This level of dependency determines to what extent crops need pollinators. In the case of forage, the degree of dependency on pollinators is set at 1 (fully dependent on pollinators) (Morse and Calderone 55), which would mean that without pollinators, there would be no production. As for the other crops that depend on pollination, their level of dependence is less: 0.2 for strawberries, 0.1 for soy and 0.1 for beans.

Also, keeping in mind your comment #5, we have adapted the value of rho to reflect the share of pollinators that are wild pollinators. In the case of forage, even though the level of dependency is set at 1, the fact that we use a value that is less than 1 for rho indicates that, even in the absence of wild pollinators, there will still be production.

3. P28 of 147 Line 271
This approach provides the gross estimate. However, the correct values should be the marginal e.g. without this park nearby farmers may have to hire bees for pollination, they may have lower yields. In either case, you need to properly frame your counter-factual. Or is the counter-factual that these croplands will not produce anything without the park being located within 2km. It should be straight forward to account for high production costs of lower yields in your equation in line 281

**Answer**: To account for the fact that native pollinators do not represent all of the available pollinators, we adapted the value of rho to reflect the contribution of native pollinators to pollination, based on data from Morse and Calderone (2000). This yields a lower value for the pollination service (10$/ha/yr for rural forests, 14$/ha/yr for urban forests) than what we obtained initially, but it also represents only the pollination service provided by native pollinators.

4. P28 of 147 Line 278
Consider replacing counting be measuring?

**Answer**: Thank you for making this suggestion; we change the wording accordingly.

5. P28 of 147 Line 282
The assumption that urban and rural forests have the same pollination contribution is a bit of a weakness. One would have thought that rural forests will be a richer habitat for native bees than urban forests and thus rural forests will have higher populations of bee/insect pollinators?

Could rho (line 281) be used to account for lower native bee pollinators when the park is not there or when it is there but of lower quality compared to its current status.

Also, could rho be used to differentiate between urban and rural forests?

The authors do allude to this fact in line 407-408.

**Answer**: We went back into our GIS data to redo the analysis to differentiate between rural and urban areas, as we have found a study that calculated wild pollinator populations in gardens in urban (New York City) and suburban areas (Westchester County) and that found a reduction of about half of the population, most likely due to the quality of the nesting environment (Fetridge et al. 2008). Although the population density in the Ottawa-Gatineau region is significantly lower than it is in New York City, and even lower than the density in Westchester county in 2000 (Westchester county: 2091-2692 people/km2; Gatineau-Ottawa: 195.6 people/km2), we decided it was more prudent to reduce the value of rho by half to differentiate between rural and urban environments.

“In our case, we used the inverse of the values provided by Morse and Calderone [55]; their value reflected the proportion of pollinators that are honey bees. This value, which represent pollinators that are not honey bees, is 0.1 for berries, 0.5 for soybeans and beans, and 0.4 for forage in rural areas. We estimate that this value (ρ) is divided by half in urban areas, considering the quality of the nesting environment [Fetridge et al. 56]. We limited our estimation to crops that have a dependence on pollinators.”

As part of the GIS analysis, we created a 1km buffer around urban agricultural croplands that grow pollinator-dependent crops (beans, berries, pastures / forages, soybeans), and then did the same for crop that were labeled as rural agricultural in our database. There was some overlap between the two buffer layers, as some urban and rural crops were somewhat closely located. As a precaution, we clipped from the rural agricultural buffer all of the area that fell in the urban agricultural buffer. Doing this, however, we ended up with some rural forests in the urban agricultural buffer. This is why the area assigned to urban forests is larger in Table 5 than the total urban forests area.

6. P29 of 147 Line 294 - 296
Please explain why the Dupras et al values are most suited for your recreation estimates. A brief explanation here will be helpful for the reader.

**Answer**: In the Dupras et al. study, we used the data on user fees taken from a report by the NCC to estimate the recreational value of ES. The NCC collects user fees only for winter recreational activities, and in the summer, for camping and for parking near beaches and some hiking trails in the Gatineau Park. As a result, this value represents only a portion of the activities undertaken in the Gatineau Park, as there is no general fee to access the park in the summer and a lot of activities can be done for free (hiking, cycling). The collected fees, however, are directly tied to activities carried out in the park.

Since the other areas of the NCC’s Green Network are all free of access, we cannot compute a value for the recreational ES of the other areas (Urban green network and the Greenbelt). Considering that the activities undertaken in the NCC’s Green Network are similar across land uses, with the exception of croplands, we believe we may use the value obtained for the recreational ES for Forests and Woodlands, Wetlands, Prairies, pastures and grassland, and Freshwater.

We have reformulated this section to reflect the fact that we will not use the data from the Environics report (27) to estimate the recreational ES, to reflect your comment #1.

7. P29 of 147 Line 297 to 306
This section only covers the social cost of carbon, how were the biophysical parameters estimated (i.e. tonnes of carbon stored and sequestered)?

**Answer**: The biophysical parameters were estimated based on other studies. For instance, the carbon storage of forests was estimated using data from the study by Kurz and Apps (86), while the carbon storage of wetlands was estimated using data from Garneau and Van Bellen (98) and the carbon sequestration of wetlands was estimated using data from Lafleur et al. (99).

These studies are mentioned explicitly in the Results section when discussing specific ecosystems. To make sure this information is clearer for the readers, we added these sentences after lines 297 to 306:

“To estimate the value of the carbon stored and sequestered by specific features of ecosystems, we relied on estimates from the literature. These specific studies are mentioned in the Results section.”

8. P29 of 147 Line 297 to 305
What was the appraisal period for your discounted cash-flow?

**Answer**: The net present value of carbon stocks were discounted over 50 years at a 3% discount rate. To make this clearer in the methods section, we added the following sentence:

“To obtain the value of carbon storage annually, we calculated the present value of carbon stocks over 50 years.”

9. P37 of 147 Line 437 to 444
Why was this approach adopted? Both the quality of the environment asset and production value of crops affect the magnitude of the estimated benefit through pollination. Both are important in putting a dollar value on the pollination ecosystem service.

**Answer**: I am not sure this explanation is very clear in our text. What we did is to divide the production value of crops that are within 1 km of forested areas by the nesting area for pollinators (forests, grassland). This way, we could infer part of the value of production (the part dependent on wild pollinators) to the nesting areas. We modified the text to make it clearer and we added an equation:

“While calculating the value of the pollinating service, we took into account the quality of the nesting environment for wild pollinators, as it is preferable to take into account the quality of the nesting environment, as well as the quality of the agricultural environment [88]. This meant dividing the value of ρ by half for urban crops, according to insights about the quality of urban environments for wild pollinators (Fetridge et al. 2008). The values were obtained by using the sum of the value of pollination for crops grown in rural areas (from Equation 1), divided by the total area in the 1 km buffer around crops that can be considered as nesting habitats for wild pollinators and then multiplied by the share of rural forests in the 1 km buffer (equation 3). We then did the same for urban forests, rural and urban prairies and grasslands.

Pollination ES = (V_pollination_Rural Total / Area_Rural buffer_ ) * % rural forests in buffer	(3)”

10. P38 of 147 Line 464 “calculating” should this be calculated?

**Answer**: Yes, thank you for noticing this error.

11. P38 of 147 Line 471
Please name and describe the “three monetary estimates” here.

**Answer**: We created a table in the Supporting information where all of the values used as part of the benefit transfer are provided in more detail. Although it makes the information less easily accessible to readers, we believe that it lightens the text. In the S1 table, the references are also available (Name of authors, year and number of the reference in the text). To make sure that readers are aware of the existence of this information, we added the following sentence at the end of the subsection “Benefit transfer with adjustment” in the Methods section.

“All of the values used as part of the benefit transfer are described in detail in [S1] table, with the country where the valuation took place, the methodology, the value in $2015 CAD/ha/yr and the ecosystem service(s) studied.”

12. P38 of 147 Line 476
Given that these are benefit transfer values, please explain what they represent within your paper as well as having their source. Even in a benefit transfer paper, it is still very important for your readers to have an idea of what a $318/ha/year for waste treatment means without the need to look at other papers. This comment applies to all your benefit transfer estimates in the paper.

**Answer**: All of the sources used for the benefit transfer are summarized in Table 3, but they are quite detailed in the Supporting Information, with the name of the authors, the country where the valuation took place, the method used to perform the valuation, as well as the value in ($/ha/year). While putting the detailed information from these studies in the Supporting Information makes it somewhat harder for readers to access, we believe it significantly lightens the text, which is already quite long.

13. P40 of 147 Line 500
Can you please include an equation showing all variables used for this estimation. It will also be helpful to show the reader your input values for the calculations.

**Answer**: The table describing the variables is available in the Methods section, more specifically in the Benefit transfer with Meta-Analysis subsection. The equation used for the meta-analysis was not in this section, but we will include it there.

14. P40 of 147 Line 510-511
While you provide a citation. Please explain the basis for the value here.

**Answer**: The values described in this paragraph are used to show the difference between the values obtained using the meta-analysis approach and values obtained using other methods. This is why, in this paragraph, we do not explain in a lot of detail how the values were obtained, aside from mentioning their valuation approaches.

Since this may not be clear, we added a sentence at the beginning of this paragraph:

“As a way of comparison, we found a number of studies that have used other methods to estimate the value of wetlands.”

15. P40 of 147 Line 527
I am unclear on what approach was used for the Gatineau Park analysis to yield the $3,338/ha/yr.

Also, the $19/ha/year to $10,741/ha/year is a very wide range. Do both numbers represent the willingness to for pay recreation at a park similar to your study area?

**Answer**: We used the same value obtained for the recreational ES as the one described in the Forests and woodland section. You commented this method in comment 1. We will change this value to the old value that we originally obtained in Dupras et al. [S1].

As for the studies mentioned, we recognize that the value are not comparable given the activities undertaken in the wetland areas which included hunting.

We changed the text in this section to reflect the lack of comparability from other studies and the new value.

“The value of recreational services of $75/ha/year was estimated based on the Gatineau Park analysis. The use of this value is based on the fact that accessible wetlands and their surrounding areas in the NCC Green Network are mainly used for birdwatching, biking, hiking, snowshoeing and cross-country skiing. Shirley’s Bay, in the Ottawa Greenbelt, is the only place where ice fishing is allowed. These are activities that some people pay to access in the Gatineau Park. It is also difficult to estimate the value of the recreational service in wetland areas specifically, due to the limited number of studies and the fact that all of the activities in the Greenbelt, where the largest wetlands are located, are free. In addition, most studies that evaluate the recreational service of wetland include fishing and hunting as part of their activities. In this case, it would be inappropriate to compare our value to these, as these activities are generally not allowed in the study area.”

16. P42 of 147 Line 548
Is this a gross or net value of production?

**Answer**: Based on the information provided in the study by Dupras et Alam, this value represents the net value of production.

“For tourism and recreational activities in agricultural lands, we used the tourism benefits associated with rural tourism in the region. We used the income from the agro-tourism of 66 agro-businesses in the region in 2005 (…).[Dupras et Alam 2014:10]” The amount obtained was divided by the agricultural land use in the region and then corrected for inflation.

17. P42 of 147 Line 549
Please explain the adjustment entails.

**Answer**: The method used for the adjustment is explained in the methods section. The adjustment is made only to take into account the PPP and the inflation rate.

“The last step of this economic valuation method was to perform an adjustment to transform values in Canadian dollars of 2015. The first step was to convert the values in Canadian dollars using purchasing power parity conversion tables (OECD stats), a method more precise than simply using exchange rates, because it takes into account the purchasing power of each currency. Then, the values in Canadian dollars were transferred into 2015 dollars using the proper inflation rates.”

18. P43 of 147 Line 555
Should the total in the first row and total value column be 4.592 rather than 4592.0?

**Answer**: No, this is the sum of the ecosystem services for croplands.

19. P46 of 147 Line 621
Are the dollar values in a per ha basis

**Answer**: Yes. We mentioned it in the title (in text), but we will add it in the notation to make this clearer visually, as this is different from the other tables.

20. P46 of 147 Line 624
What is meant by K$ if thousands please specify fully, K$ often used to refer to constant dollars.

**Answer**: Yes, K$ represents the value in thousands. We will changed the notation in the table heading to **($ ‘000 CAD 2015).**

21. P48 of 147 Line 624
Should this line start with “66% of the estimated economic value ...”

**Answer**: This is a good suggestion, thank you. We changed the beginning of the sentence.
